# Reducing unemployment benefit duration to increase job finding rates: a systematic review

**DOI:** 10.4073/csr.2018.2

**Published:** 2018-02-28

**Authors:** Trine Filges, Anders Bruun Jonassen, Anne‐Marie Klint Jørgensen

## Abstract

This Campbell systematic review examines the impact of reducing the maximum duration of unemployment benefits on job‐finding rates. Seven studies were included in the review, all of which are from European countries.

Included studies had to examine the effect of a reduction in the maximum duration of entitlement of any kind of unemployment benefits on employment using a well‐defined control group.

Whilst 41 studies were identified, after allowing for study quality and data issues, only seven studies were included in the review. The included studies covered Austria (2 studies), France, Germany (3 studies) and Slovenia. Maximum entitlement ranged between 26 and 209 weeks. The studies analyzed reductions between 9 and 179 weeks, with an average of 43 weeks. The studies analyze data from 1,154,090 unemployment spells.

Reducing the duration of unemployment benefits increases the exit rate from unemployment. Data from seven studies show that the exit rate from unemployment for those with reduced duration of benefit entitlement on average is 10 per cent. This corresponds to a 52% chance that those with reduced duration will find a job before an unemployed person with the existing, longer duration (no effect corresponds to a 50% chance).

There is not enough evidence to determine effects on the exit rate from re‐employment or on the wage rate in the job found. There are insufficient high‐quality studies to allow an examination of variation of effects.

**Plain language summary:**

**Reducing the maximum duration of unemployment benefits increases the job finding rate of the unemployed**

Reducing the maximum duration of unemployment benefits is one strategy used to reduce unemployment. Evidence from seven studies confirms such an effect. However, the effect is small and more studies of higher quality are needed to give more detailed findings to inform policy.

**The review in brief**

Evidence from seven studies shows shortening the maximum duration of unemployment benefit entitlement has a small effect on the job finding rate of the unemployed.

**What is this review about?**

Policymakers may wish to reduce the generosity of the unemployment benefits system in order to reduce unemployment levels. Reducing benefit levels may be politically more difficult than shortening the length of the unemployment benefit eligibility period to create work incentives for the unemployed.

This review summarizes studies that measure the effects of shortening the maximum duration of unemployment benefit entitlement on job finding rates.

**What is the aim of this review?**

This Campbell systematic review examines the impact of reducing the maximum duration of unemployment benefits on job‐finding rates. Seven studies were included in the review, all of which are from European countries.

**What are the main findings of this review?**

**What studies are included?**

Included studies had to examine the effect of a reduction in the maximum duration of entitlement of any kind of unemployment benefits on employment using a well‐defined control group.

Whilst 41 studies were identified, after allowing for study quality and data issues, only seven studies were included in the review. The included studies covered Austria (2 studies), France, Germany (3 studies) and Slovenia. Maximum entitlement ranged between 26 and 209 weeks. The studies analyzed reductions between 9 and 179 weeks, with an average of 43 weeks. The studies analyze data from 1,154,090 unemployment spells.

**What are the main results of this review?**

Reducing the duration of unemployment benefits increases the exit rate from unemployment. Data from seven studies show that the exit rate from unemployment for those with reduced duration of benefit entitlement on average is 10 per cent. This corresponds to a 52% chance that those with reduced duration will find a job before an unemployed person with the existing, longer duration (no effect corresponds to a 50% chance).

There is not enough evidence to determine effects on the exit rate from re‐employment or on the wage rate in the job found. There are insufficient high‐quality studies to allow an examination of variation of effects.

**What do the findings of this review mean?**

On the basis of this limited number of studies, shortening the maximum duration of unemployment benefit entitlement has a small effect on the job finding rate of the unemployed. Whether unemployed workers responding to a shorter potential benefit entitlement may be worse off, in the sense that they accept “lower quality” jobs, has not yet been fully investigated.

But the review finds a surprisingly low number of studies with a sufficiently low risk of bias to be used for synthesis to determine the effect size of shortening the maximum duration of unemployment benefit entitlement. Many studies had to be excluded as they had a high risk of bias. This is a finding in its own right.

There is a need for future studies to more thoroughly discuss the assumptions of the study design and justify the choice of method by considering and reporting all relevant data and tests. Future studies should also use data with all relevant information, in particular, information on whether eligible individuals actually received unemployment benefits and information on individual maximum entitlement duration.

**How up‐to‐date is this review?**

The review authors searched for studies published up to December 2016. This Campbell Systematic Review was published in February 2018.

**Executive Summary/Abstract:**

**BACKGROUND**

Unemployment benefit programmes protect individuals against loss of income and provide unemployed individuals with the possibility of finding a better match between their qualifications and job vacancies. However, unemployment benefits may also distort incentives by subsidizing long and unproductive job searches. In order to reduce unemployment levels, policymakers may wish to reduce the generosity of the unemployment system. While it may be politically intractable to lower the monetary amount of unemployment benefits available, the length of the unemployment benefit eligibility period is often used as a political instrument to create work incentives for the unemployed. If a shorter benefit period results in a significantly increased incentive for finding work, shortening the benefit eligibility period may reduce the share of long and unproductive job searches and thereby decrease the overall unemployment level.

**OBJECTIVES**

The purpose of this review is to systematically uncover relevant studies in the literature that measure the effects of shortening the maximum duration of unemployment benefit entitlement on job finding rates, and to synthesize the effects in a transparent manner. As a secondary objective we will, where possible, investigate the extent to which the effects differ among different groups of unemployed people, such as those with high/low levels of education or men/women, and further explore from which point in the unemployment spell unemployed individuals react to the length of benefit entitlement.

**SEARCH METHODS**

The search was concluded in March 2016. Relevant studies were identified through electronic searches of bibliographic databases, government policy databanks, internet search engines and hand searching of core journals. We searched to identify both published and unpublished literature. The searches were international in scope. Reference lists of included studies and relevant reviews were also searched.

**SELECTION CRITERIA**

The intervention of interest was a reduction (change) in the maximum duration of entitlement of any kind of unemployment benefits. We included unemployed individuals who received any type of time‐limited benefit during their unemployment spell. All study designs that used a well‐defined control group were eligible for inclusion in this review. Studies that utilised qualitative approaches were not included in the review due to the absence of adequate control group conditions.

**DATA COLLECTION AND ANALYSIS**

Random effects models were used to pool data across the studies. We used the point estimate of the hazard ratio. Pooled estimates were weighted with inverse variance methods, and 95% confidence intervals were used. A sensitivity analysis was performed to evaluate whether the pooled effect sizes were robust across components of methodological quality, in relation to the quality of data and whether the study analysed an extension of entitlement duration.

**RESULTS**

The initial search for potentially relevant studies resulted in a total number of 34,930 hits. A total of 41 studies, consisting of 66 papers, from 15 different countries, met the inclusion criteria and were vetted by the review authors. Only 38 studies provided data that permitted the calculation of an effect size for the primary outcome. Of these 38 studies, 28 studies could not be used in the data synthesis due to a too high risk of bias. A further 3 studies could not be used in the data synthesis due to overlapping of data samples. As a result, only 7 studies were included in the data synthesis and one of these studies only provided results on the secondary outcome. In total, 6 studies provided data that permitted the calculation of an effect size for the primary outcome and 3 studies provided data that permitted the calculation of secondary outcome. The sample size used in the studies ranged from 5,017 spells of unemployment to 509,355 spells. The total number of unemployment spells was 1,154,090, implying an average sample size of 164,870 spells of unemployment per study.

The seven studies covered Austria, France, Germany and Slovenia. There was a high degree of variation in maximum entitlement, ranging between 26 and 209 weeks. On average the studies analysed a reduction of 43 weeks in maximum entitlement; the smallest being a reduction of 9 weeks and the largest a reduction of 179 weeks. Four studies restricted the analysis to a specific age group and three studies restricted the analysis to specific work experience levels. All studies used non‐randomised designs. In the majority of studies the risk of bias was high.

This review found a statistically significant effect of shortening the maximum duration of unemployment benefit entitlement. The overall impact of shortening the maximum duration of unemployment benefit entitlement, obtained using hazard ratios, was estimated at 1.10 (95% CI 1.03 to 1.17, p=0.0005), which translates into an increase of approximately 10% in the exit rate from unemployment into employment and corresponds to a 52% chance that a treated unemployed person will find a job before a non‐treated unemployed person.

Thus, although small, the available evidence associated with a sufficiently low risk of bias supports the hypothesis of an incentive effect of shortening the maximum duration of unemployment benefit entitlement. There was a lack of evidence to conclude that shortening the maximum duration of unemployment benefit entitlement has an impact on the quality of the job obtained.

Only three studies provided data on the exit rate from re‐employment and three studies provided data on the log wage ratio in the re‐employment job.

The overall impact of shortening the maximum duration of unemployment benefit entitlement on the exit rate from the re‐employment job, obtained using hazard ratios, was 0.99 (95% CI 0.97 to 1.02, p=0.64) and the overall wage ratio was 1.00 (95% CI 0.99 to 1.01, p=0.089).

We did not find any adverse effects.

Sensitivity analyses resulted in no appreciable change in effect size, suggesting that the results are robust. The limited number of studies used in the meta‐analysis should, however, be considered when interpreting the results.

Due to having an insufficient number of studies available for moderator analysis to be performed, it was not possible to examine whether the effect of reducing the maximum potential benefit duration on job‐finding differs for men and women, for particular age groups or educational groups, or if factors such as good or bad labour market conditions, the type of unemployment benefit, the availability of alternative benefits, or whether compulsory activation is part of the institutional system, have an impact on the effect.

**AUTHORS' CONCLUSIONS**

To the best of our knowledge, this is the first systematic review analysing the magnitude of the effect of shortening the maximum duration of unemployment benefit entitlement on the job finding rate. The review finds a surprisingly low number of studies with a sufficiently low risk of bias to enter a synthesis of the effect size of shortening the maximum duration of unemployment benefit entitlement. On the basis of this limited number of studies, shortening the maximum duration of unemployment benefit entitlement displays a limited potential for altering the employment prospects of the unemployed individuals. The available evidence does suggest an effect on the job finding rate of shortening the maximum duration of unemployment benefit entitlement, but the effect is small. Further, whether unemployed workers responding to a shorter potential benefit entitlement may be worse off, in the sense that they accept “lower quality”” jobs, has not yet been fully investigated.

The number of studies used in the data synthesis (7) is relatively low compared to the large number of studies (41) meeting the inclusion criteria for the review. The reduction in studies eligible for inclusion in the data synthesis was primarily caused by a judgment of too high a risk of bias. Thus, the process of excluding studies with too high risk of bias from the meta‐analysis applied in this review left us with only seven studies to synthesize. This is a finding in its own right, entailing important information for stakeholders on the degree of confidence to place on the expected gains from changing the maximum potential unemployment benefit duration; fewer studies with too high risk of bias would have provided a more robust literature on which to base conclusions. There is a need for future studies to more thoroughly discuss the identifying assumptions of the study design and justify the choice of method by considering and reporting all relevant data and tests. Further, future studies should rely on data where all relevant information is available, in particular information on whether eligible individuals actually received unemployment benefits and information on individual maximum entitlement duration.

## 1 Background

### 1.1 THE PROBLEM, CONDITION OR ISSUE

Benefit programmes protect individuals against loss of income and provide unemployed individuals with the possibility of finding a better match between their qualifications and job vacancies. This positive aspect of inducing workers to achieve better job matches has been shown, theoretically, to potentially increase economic efficiency (Acemoglu & Shimer, 1999; Marimon & Zilibotti, 1999).

However, unemployment benefits may also distort incentives by subsidizing long and unproductive job searches. In fact, the generosity of unemployment benefits is generally considered the main factor by which benefit systems affect unemployment. From a societal point of view, therefore, the optimal unemployment benefit system will balance considerations for protection with those for distortion (Feldstein, 2005; Mortensen, 1987).

Theory suggests that putting a limit on benefit duration will tend to accelerate job search from the beginning of the unemployment spell and thereby shorten unemployment duration (Pissarides, 2000). Thus, generosity of benefits is determined not only by the amount paid but also by the duration of benefit entitlement. In the US, replacement rates^1^ are low and duration is short compared to benefit systems in most European countries. In 2005, the maximum duration of unemployment insurance entitlement among OECD countries^2^ was shortest in the US at 6 months^3^ and longest in Denmark, Norway, Portugal, the Netherlands, France, Finland and Spain, varying between 23 and 48 months (OECD, 2007). At the same time, the gross initial replacement rate was around 50% in the US, while varying between 62% and 90% in the aforementioned European countries.

The lower level of generosity of benefits in the US compared to Europe is consistent with the observation of higher levels of active searches and a greater willingness to accept inferior jobs by unemployed workers in the US compared to Europe (Layard, Nickell & Jackman, 2005). As a consequence, European policy‐makers may consider reducing the generosity of unemployment systems in order to reduce high unemployment levels^4^. While lowering the replacement rate may be politically intractable (indeed, examples of reductions of benefit rates and amounts are rare), the length of the unemployment benefit entitlement period is often used as a political instrument to improve work incentives for the unemployed. In Spain, for example, the benefit period was altered in 1992, in Slovenia in 1998, in Norway in 1997, in the UK in 1996, in Denmark in 1996, 1998 and 1999, and, more recently, in the Czech Republic in 2004, in Hungary and Portugal in 2006, and in Denmark again in 2010.

A crucial public policy question is whether a more generous unemployment benefit system is causally related to higher unemployment rates. As pointed out in Card and Riddell (1993), there are several complementary potential explanations for differences in unemployment rates between countries, including differences in the overall distributions of working and nonworking time, and differences in the fraction of nonworking time that is reported as unemployment (particularly among individuals with very low levels of labour supply). Recent research on the effect of extended duration of unemployment insurance benefits in the US shows that benefit extensions raised the unemployment rate, but at least half of the effect is attributable to reduced labour force exit among the unemployed rather than to the changes in reemployment rates that are of greater policy concern (Rothstein, 2011).

This review focuses on the effect on job finding rates of reducing the maximum duration of entitlement of unemployment benefits, and secondarily on the effects on the quality of these re‐employment jobs.

### 1.2 THE INTERVENTION

The intervention of interest is reduction^5^ in the maximum duration of entitlement of any kind of unemployment benefit with a known expiration date. The benefits may be unemployment insurance (UI) benefits or unemployment assistance (UA)/social assistance (SA) benefits, as long as they have a known expiration date.

In the majority of OECD countries, the UI benefit has a time limit. In fact, only Belgium has an unlimited UI period. In other countries, the maximum duration varies between 6 months (as for example in the UK and the US) and 36 months (in Iceland).

In most OECD countries, a secondary benefit is available for those who have exhausted regular UI benefits. These are known as SA benefits. Unlike UI benefits, SA benefits are generally means‐tested without any necessary connection to past employment; they pay a lower level of benefit and are indefinite. We know of only one example of an SA benefit with a time limit: the Temporary Assistance to Needy Families (TANF) which is available in the US. The federal government requires states to impose between 2‐ or 5‐year limits on TANF (Gustafson & Levine, 1997). In a minority of OECD countries, UA benefits are paid after exhaustion of UI benefits. Like SA benefits, they are generally means‐tested, pay a lower level of benefits and, excepting Hungary, Portugal and Sweden, are indefinite.

Unemployment benefits with an indefinite time limit or non‐financial benefits are excluded from this review.

### 1.3 HOW THE INTERVENTION MIGHT WORK

Search theory offers an explanation for how reducing unemployment benefits duration might increase job finding rates. According to search theory, one can derive a relationship between the job finding rate and the parameters of the benefit system, in particular the maximum benefit duration and the replacement rate (Mortensen, 1977). This relationship is driven by adjustments in search effort and reservation wages. The reservation wage is the minimum wage at which the unemployed are willing to accept a job. Forward‐looking unemployed workers chose their current search effort and reservation wage in order to maximize the sum of the utility flow realized during the current period, plus the expected discounted future utility flow given that an optimal strategy will be pursued in every future period. The current search effort and reservation wage are thus affected by the future level of benefits. When the benefit period expires, the unemployed person experiences a potentially large drop in income. As the time of benefit exhaustion approaches, the value to that person of remaining unemployed falls, implying a higher search effort and/or a fall in the reservation wage, leading to a higher exit rate out of unemployment (Mortensen, 1977). This non‐stationarity implies that unemployed individuals with different lengths of benefit entitlement have different optimal paths of reservation wage and search effort over time (van den Berg 1990).

A shorter entitlement period gives the unemployed individual a stronger incentive to quickly gain employment in order to avoid the drop in income after the exhaustion date. How strong the incentive is depends on the magnitude of the income drop. If no secondary benefit is available for those who have exhausted their current benefit, the incentive to gain employment will be stronger. If an increased job finding rate is mainly driven by lowering the reservation wage, a lower job match quality is to be expected, for example, in the form of lower wages and/or lower re‐employment duration.

A number of factors may have an impact on the magnitude of the expected increase in the job finding rate. In general, the overall labour market conditions (i.e. the vacancy rate^6^ and, in particular, the unemployment rate) have an impact on the availability of and competition for jobs. If the vacancy rate is high (i.e. the number of vacancies is high in relation to job seekers) we would expect a bigger effect on job finding rates than if the vacancy rate is low. We would further expect a lower effect if the unemployment rate is high, regardless of the vacancy rate. If the vacancy rate is low coincident with a high unemployment rate, competition for available jobs is likely to be high. If the vacancy rate is high coincident with a high unemployment rate, it suggests mismatch in the labour market (i.e., the process by which vacant jobs and job seekers meet is not efficient) (Filges & Larsen, 2000; Pissarides, 2000).

Whether compulsory participation in active labour market programmes is part of the unemployment system may also have an impact on the effect of maximum duration of entitlement. The compulsory aspect of activation may provide an incentive for unemployed individuals to look for and return to work prior to programme participation; the so called threat effect. Filges and Hansen (2015) summarize the available evidence on the threat effect of active labour market programmes and report a significant threat effect of compulsory participation in active labour market programmes. Further, actual participation in active labour market programmes may improve some of the participants' qualifications, thus helping them to find a job. Alternatively, active labour market programmes may have negative stigmatization and signalling effects to employers. Programmes associated with participants having poor employment prospects may carry a stigma. Because of asymmetric information, employers do not know the productivity of new workers, some of whom they might hire from the pool of the unemployed. Prospective employers might then perceive participants in such programmes as low productivity workers or workers with tenuous labour market attachment (Kluve et al. 1999; Kluve et al., 2007).

A recent systematic review by Filges et al. (2015b) investigated the effect of participating in active labour market programmes and found that there is a significant positive effect, although small, of participating in active labour market programmes. The effect reported in Filges et al. (2015b) is, however, a pure post‐programme effect of active labour market programmes; it refers to the period after participation in a programme. The net effect of active labour market programme participation on job finding rates is, however, composed of two separate effects: a lock‐in effect and a post‐programme effect. The lock‐in effect refers to the period of participation in a programme. During this period, job‐search intensity may be lowered because there is less time to search for a job, and participants may want to complete an on‐going skill‐enhancing activity; hence the lock‐in effect. The combination of the two effects, lock‐in and post‐programme, consequently determines the net effects of active labour market programme participation on unemployment duration.

These additional effects on the search behaviour and employment prospects when compulsory participation in active labour market programmes is part of the unemployment system may dampen the observed effects of maximum duration of entitlement on job finding rates.

Finally, the type of unemployment benefit may have an impact on the effect on the job finding rate. As mentioned above, some countries employ two systems to provide benefits to unemployed individuals: an unemployment insurance system for individuals who typically have a strong labour market attachment (UI benefits) and a social welfare system for individuals who often have other problems in addition to unemployment (SA or UA benefits). The effect size in social welfare systems offering unemployment benefits with a known expiration date is, due to the participants' lower labour market attachment, expected to be less than the effect size in unemployment insurance systems with a known expiration date.

### 1.4 WHY IT IS IMPORTANT TO DO THE REVIEW

In order to reduce unemployment levels, policy‐makers may wish to reduce the generosity of the unemployment system either in amount (the replacement rate) or in maximum potential duration.

The positive correlation between unemployment duration and the replacement rate is well established at the empirical level (Layard et al., 2005). However, it may be politically intractable to lower the replacement rate, and there are indeed strong efficiency and equity arguments for having a reasonable value of unemployment benefits (Acemoglu & Shimer, 1999; Marimon & Zilibotti, 1999).

Search theory suggests that an increase maximum duration of benefit entitlement has a negative impact on the job search activities of the unemployed, thus increasing their unemployment duration. Indeed, although the effect is small, there is clear evidence that the prospect of exhausting benefits results in a significant increase in job finding (Filges et al., 2013).

Hence, shortening the benefit eligibility period may reduce the share of long and unproductive job searches somewhat. The conclusion in Filges et al. (2013), however, leaves unanswered the question of how much of a reduction in maximum unemployment benefit entitlement decreases unemployment duration.

There are many empirical papers on the effect of maximum benefit entitlement on unemployed individuals (Caliendo, Tatsiramos and Uhlendoff 2009; Bennmarker, Carling & Holmlund, 2007; Ham & Rea, 1987; Hunt, 1995; Katz & Meyer, 1990 and Lalive & Zweimüller, 2004), but the empirical research has not been summarized in a systematic review to obtain a clearer picture of the available evidence on the employment effect of reducing maximum duration of benefit entitlement.

Fredriksson and Holmlund (2006) provide a non‐systematic review of the literature on how incentives in unemployment insurance can be improved, but do not make the important distinction between exits to employment and exits to other destinations such as such as other kinds of benefits or out of the labour force. As shown in Card, Chetty and Weber (2007), the exit rate from registered unemployment can increase by more than 10 times than that of the rate of re‐employment at the expiration of benefits. The difference between the two measures arises because many individuals leave the unemployment register immediately after their benefits expire without returning to work.

There is a great deal of political interest in optimizing the unemployment benefit system to balance concerns for an adequate social safety net with concerns for the implicit distortionary effects of providing such safety net.

A less generous unemployment benefit system provides less protection from the consequences of involuntary unemployment, but is also expected to increase unemployment by diminishing efforts to gain re‐employment. Achieving the desired balance between the apparently conflicting goals of ensuring both a sufficiently high level of protection and a sufficiently low level of unemployment requires reliable information on the effect size of altering benefit generosity on job finding. To the best of our knowledge, no systematic review exists on the magnitude of this effect. This review provides the important contribution of synthesizing existing effect size estimates through a systematic review of the empirical literature on reducing the maximum duration of unemployment benefit entitlement on employment probabilities.

## 2 Objectives

The purpose of this review is to systematically uncover relevant studies in the literature that measure the effects of shortening the maximum duration of unemployment benefit entitlement on job finding rates, and to synthesize the effects in a transparent manner. As a secondary objective we will, where possible, investigate the extent to which the effects differ among different groups of unemployed such as those with high/low levels of education or men/women, and further explore from which point in the unemployment spell unemployed individuals react to the length of benefit entitlement.

## 3 Methods

### 3.1 TITLE REGISTRATION AND REVIEW PROTOCOL

The title for this systematic review was registered July, 2015. The systematic review protocol (Filges, Jonassen & Jørgensen, 2015a), was published November, 2015. Both the title registration and the protocol are available in the Campbell Library at: https://www.campbellcollaboration.org/library/reducing‐unemployment‐benefit‐duration‐to‐increase‐job‐finding‐rates.html

### 3.2 CRITERIA FOR CONSIDERING STUDIES FOR THIS REVIEW

#### 3.2.1 Types of studies

The study designs eligible for inclusion were:

- Controlled trials:
  ○ RCT ‐ randomized controlled trial
  ○ QRCT ‐ quasi‐randomized controlled trial (i.e., participants are allocated by means such as alternate allocation, person's birth date, the date of the week or month, or alphabetical order)
  ○ NRCT ‐ non‐randomized controlled trial (i.e. participants are allocated by other actions controlled by the researcher)
- Non‐randomized studies (NRS) where allocation is not controlled by the researcher and two or more groups of participants are compared. Participants are allocated by means such as time differences, location differences, decision‐makers, or policy rules.

Study designs that used a well‐defined control group were eligible. The main control or comparison condition was no change in maximum duration of benefit entitlement.

Non‐randomized studies, where the reduction in maximum duration of benefit entitlement has occurred in the course of usual decisions outside the researcher's control must have demonstrated pre‐treatment group equivalence via matching, statistical controls, or evidence of equivalence on key risk variables (e.g., labour market conditions) and participant characteristics. These factors are outlined in section 3.3.3 under the subheading of *Confounding*, and the methodological appropriateness of the included studies was assessed according to the risk of bias model outlined in section 3.3.3.

Studies of the effect of reducing unemployment benefit entitlement typically are estimated on data collected from administrative registers or by questionnaires. Studies that used different data sources for treatment and control groups were not eligible.

Only studies that used individual micro‐data were eligible. Studies that relied on regional or national time series data were not eligible, even though micro‐econometric estimates of individual effects merely provide partial information about the full impact of shortening the maximum duration of benefit entitlement (Calmfors, 1994; Calmfors, 1995).

We included studies irrespective of their publication status, and their electronic availability.

#### 3.2.2 Types of participants

We included unemployed individuals who received some sort of time limited benefit during their unemployment spell. The International Labour Office (ILO) definition of an unemployed individual is a person, male or female, aged 15‐74, without a job who is available for work and either has searched for work in the past four weeks or is available to start work within two weeks and/or is waiting to start a job already obtained (ILO, 1990); however, different countries may apply different definitions of an unemployed individual, see for example Statistics Denmark (2009). We included participants receiving all types of unemployment benefits with a known exhaustion date. The only restriction was that the benefits must be related to being unemployed. We therefore excluded individuals who only received other types of benefits not related to being unemployed. We included all unemployed participants regardless of age, gender, etc. who received some sort of time limited benefit during their unemployment spell.

#### 3.2.3 Types of interventions

The intervention was reduction^7^ in the maximum duration of entitlement of any kind of unemployment benefits. The benefits may be unemployment insurance (UI) benefits or they may be unemployment assistance (UA)/social assistance (SA). The only requirement was that the benefit must have a known expiration date. The UI benefit usually has a known time limit whereas UA and SA usually are indefinite. Unemployment benefits with an indefinite time limit or non‐financial benefits were excluded from this review.

#### 3.2.4 Types of outcome measures

The objective was to determine whether reducing the maximum entitlement to unemployment benefits motivates unemployed individuals to find a job more quickly. Distinguishing between destinations was therefore vital. The primary outcome was exits to employment. Studies only looking at exits to other destinations such as other types of social benefits or non‐employment and studies that do not distinguish between destinations were not eligible.

We considered secondary outcomes in terms of the impact that reducing the maximum duration of entitlement of benefit has on the duration of re‐employment and on income. This was done in order to obtain a clearer picture of the effect that reducing the maximum entitlement of unemployment benefit has on the quality of the job. If the duration of re‐employment or the wage is low, this could indicate that reducing entitlement forces unemployed individuals to find jobs that do not match their qualifications and therefore they may return to unemployment more quickly.

*Primary outcomes*

Primary outcomes we planned to include refer to employment status:

- exit rate, measured as a hazard rate, from unemployment to employment (= work with standard wages and which anyone can apply for)
- proportion employed (= proportion of participants who have obtained work with standard wages and which anyone can apply for)
- duration until employment (= work with standard wages and which anyone can apply for)

*Secondary outcomes*

Secondary outcomes we planned to include were:

- duration of first employment spell post‐intervention
- re‐employment wage

#### 3.2.5 Duration of follow‐up

Outcomes measured as hazard ratios were reported as an overall effect on the hazard rate, and in addition some were reported separately for different unemployment duration intervals. All time points reported were considered.

#### 3.2.6 Types of settings

All types of settings were eligible.

### 3.3 SEARCH METHODS FOR IDENTIFICATION OF STUDIES

Identification of studies were based on updated searches from an earlier similar Campbell review (Filges et al., 2013). The first part of the search period (from 1985 to March 2011) was covered by re‐examining results of the searches for that review, where an identical search strategy was used. The search documentation described in 3.3.1‐3.3.5 cover the two updates from 2011‐2015 and from 2015‐2016. The search was performed by one review author (AKJ) and one member of the review team (BVN)^8^.

#### 3.3.1 Electronic searches

Relevant studies were identified through electronic searches of bibliographic databases. The following bibliographic databases were searched:

- PsycInfo (searched through EBSCO) – Latest search performed 11/3‐2016.
- SocIndex (searched through EBSCO) – Latest search performed 11/3‐2016.
- Econlit (searched through EBSCO) – Latest search performed 26/2‐2016.
- Business Source Complete (searched through EBSCO) – Latest search performed 10/3‐2016.
- IBSS: International Bibliography of the Social Sciences (searched through ProQuest) – Search performed 10/3‐2016.
- ProQuest Dissertations and Theses (searched through ProQuest) – Latest search performed 10/3‐2016.
- SSCI: Social Science Citation Index & SCI: Science Citation Index (searched through Web of Science) – Latest search performed 10/3‐2016.

#### 3.3.2 Search terms

An example of the search string used to search SocIndex from the 2015‐2016 update is listed in section 12.1. The search string was modified in accordance to the different search terminology on the databases searched.

#### 3.3.3 Searching other resources

We examined the reference lists (snowballing/citation‐tracking) from relevant reviews and studies identified in the electronic searches, and from included primary studies for studies that potentially met inclusion criteria.

#### 3.3.4 Searching for Unpublished/Grey Literature

Searching for unpublished/grey literature was performed by searching governmental repositories, evidence‐based practice repositories (such as clearinghouses) and internet search engines. The search strategy for the grey literature search was based on the search string for the electronic database search. Due to the limited search capacity on grey literature information resources, web pages and search engines, a shortened search string was used. An example of the search strategies used to identify grey literature and google searches can be found in section 9.1. The most recent search for grey literature was performed between 26^th^ of February and 10^th^ of March 2016. The following websites, repositories and resources were searched for relevant grey literature:

- Cochrane Library (http://www.cochranelibrary.com/)
- Forskningsdatabasen ‐ The Danish National Research Database (http://www.forskningsdatabase.dk/)
- Social Care Online (http://www.scie‐socialcareonline.org.uk/)
- IBZ – De Gruyter (https://www.degruyter.com/view/db/ibz)
- SSRN: Social Science Research Network (https://www.ssrn.com/en/)
- IDEAS (https://ideas.repec.org/)
- OpenGrey (http://www.opengrey.eu/)
- IZA – Institute of the Study of Labor (www.iza.org)
- CEPR – Centre for Economic Policy Research (www.cepr.org)
- NBER – National Bureau of Economic Research (www.nber.org)
- MDRC – the Manpower Demonstration Research Corporation – (www.mdrc.org)
- Danish Economic Councils (www.dors.dk)
- OECD ‐ the Organisation for Economic Co‐operation and Development (www.oecd.org)
- IMF ‐ The International Monetary Fund (www.imf.org)
- AIECE ‐ Association of European Conjuncture Institutes (www.aiece.org)
- ESRC ‐ Economic Social Research Council (www.esrc.ac.uk)
- Copenhagen Economics (www.copenhageneconomics.com)
- Google Scholar (https://scholar.google.dk/)

Due to changes in access possibilities, Theses Canada was not searched after the 2011 update.

#### 3.3.5 Hand searching

Reference lists of included studies and reference lists of relevant reviews was searched. “The Journal of Labor Economics” and “Labour Economics” was searched for the years 2011‐2015 and the available issues of 2016 up to March:

- Labour Economics (issn: 0927‐5371) ‐ Latest search was performed 15/3‐2016.
- Journal of Labour Economics (issn: 0734‐306X) ‐ Latest search was performed 15/3‐2016.

### 3.4 DATA COLLECTION AND ANALYSIS

#### 3.4.1 Selection of studies

Under the supervision of review authors, two review team assistants first independently screened titles and abstracts to exclude studies that were clearly irrelevant. Studies considered eligible by at least one assistant or studies were there was insufficient information in the title and abstract to judge eligibility, were retrieved in full text. The full texts were then screened independently by two review team assistants under the supervision of the review authors. Any disagreement of eligibility was resolved by the review authors. Exclusion reasons for studies that otherwise might be expected to be eligible were documented and presented in Section 11.

The study inclusion criteria were piloted by the review authors (see Appendix 2.3). None of the review authors were blind to the authors, institutions, or the journals responsible for the publication of the articles.

#### 3.4.2 Data extraction and management

Two review authors independently coded and extracted data from included studies. A coding sheet was piloted on several studies (See Appendix 2.3 and 2.4). Disagreements were resolved by discussion.

Information was extracted on: available characteristics of participants, intervention characteristics, research design, sample size, time period, outcomes, and results. Extracted data were stored electronically. Analysis was conducted in RevMan 5.

#### 3.4.3 Assessment of risk of bias in included studies

Two review authors independently assessed the risk of bias for each included study. There were only minor disagreements and they were resolved by discussion.

We assessed the risk of bias using a model developed by Prof. Barnaby Reeves in association with the Cochrane Non‐Randomised Studies Methods Group (Reeves, Deeks, Higgins, & Wells, 2011).^9^ This model is an extension of the Cochrane Collaboration's risk of bias tool and covers risk of bias in non‐randomised studies that have a well‐defined control group.

The extended model is organised and follows the same steps as the risk of bias model according to the 2008 version of the Cochrane Handbook, chapter 8 (Higgins & Green, 2008). The extension to the model is explained in the three following points:

1) The extended model specifically incorporates a formalised and structured approach for the assessment of selection bias in non‐randomised studies by adding an explicit item that focuses on confounding^10^. This is based on a list of confounders considered important and defined in the protocol for the review. The assessment of confounding is made using a worksheet, which is marked for each confounder according to whether it was considered by the researchers, the precision with which it was measured, the imbalance between groups, and the care with which adjustment was carried out (see Appendix 2.5). This assessment informs the final risk of bias score for confounding.
2) Another feature of non‐randomised studies that make them at high risk of bias is that they need not have a protocol in advance of starting the recruitment process. The item concerning selective reporting therefore also requires assessment of the extent to which analyses (and potentially other choices) could have been manipulated to bias the findings reported, e.g., choice of method of model fitting, potential confounders considered/included. In addition, the model includes two separate yes/no items asking reviewers whether they think the researchers had a pre‐specified protocol and analysis plan.
3) Finally, the risk of bias assessment is refined, making it possible to discriminate between studies with varying degrees of risk. This refinement is achieved by the use of a 5‐point scale for certain items (see the following section *Risk of bias judgement items* for details).

The refined assessment is pertinent when considering data synthesis as it operationalizes the identification of those studies with a very high risk of bias (especially in relation to non‐randomised studies). The refinement increases transparency in assessment judgements and provides justification for excluding a study with a very high risk of bias from the data synthesis.

##### Risk of bias judgement items

The risk of bias model used in this review is based on 9 items (see Appendix 2.5).

The 9 items refer to:

- **sequence generation** (Judged on a low/high risk/unclear scale)
- **allocation concealment** (Judged on a low/high risk/unclear scale)
- **confounders** (Judged on a 5 point scale/unclear)
- **blinding** (Judged on a 5 point scale/unclear)
- **incomplete outcome data** (Judged on a 5 point scale/unclear)
- **selective outcome reporting** (Judged on a 5 point scale/unclear)
- **other potential threats to validity** (Judged on a 5 point scale/unclear)
- **a priori protocol** (Judged on a yes/no/unclear scale)
- **a priori analysis plan** (Judged on a yes/no/unclear scale)

In the 5‐point scale, 1 corresponds to Low risk of bias and 5 corresponds to High risk of bias. A score of 5 on any of the items assessed on the 5‐point scale translates to a risk of bias so high that the findings will not be considered in the data synthesis (because they are more likely to mislead than inform).

##### Confounding

An important part of the risk of bias assessment of non‐randomised studies is consideration of how the studies deal with confounding factors (see Appendix 212.5). Selection bias is understood as systematic baseline differences between groups which can therefore compromise comparability between groups. Baseline differences can be observable (e.g. age and gender) and unobservable to the researcher (e.g. motivation and ‘ability’). There is no single non‐randomised study design that always solves the selection problem. Different designs represent different approaches to dealing with selection problems under different assumptions, and consequently require different types of data. There can be particularly great variations in how different designs deal with selection on unobservables. The “adequate” method depends on the model generating participation, i.e. assumptions about the nature of the process by which participants are selected into a programme. A major difficulty in estimating causal effects of the maximum duration of benefit entitlement is the potential endogeneity of the change to benefit rules stemming from the policy process that leads to the change.

The determinants of the change are often labour market conditions and if not accounted for it yields biased estimates.

As there is no universal correct way to construct counterfactuals for non‐randomised designs, we looked for evidence that identification is achieved, and that the authors of the primary studies justified their choice of method in a convincing manner by discussing the assumption(s) leading to identification (the assumption(s) that make it possible to identify the counterfactual). Preferably the authors should make an effort to justify their choice of method and convince the reader that the only difference between an individual with a short maximum benefit period and an individual with a longer maximum benefit period is exactly the difference in length of maximum benefit period and that the source of difference between their entitlement status is not endogenous to the individuals' exit rate to employment. The judgement is reflected in the assessment of the confounder unobservables in the list of confounders considered important at the outset (see Appendix 2.5).

In addition to unobservables, we identified the following observable confounding factors to be most relevant: age, gender, education, ethnicity, labour market conditions, and unemployment duration. In each study, we assessed whether these factors had been considered, and in addition we assessed other factors likely to be a source of confounding within the individual included studies.

##### Importance of pre‐specified confounding factors

The motivation for focusing on age, gender, education and ethnicity is that they are the major determinants of the risk of being unemployed (Layard et al., 2005).

Another potential source of bias is differences in labour market conditions. If a study, for example, explores changes in the maximum duration of benefit entitlement over time or space as the source of variation, it is very important to control for changes in labour market conditions over time (as a consequence of the business cycle, for example) or over space as the exit rate to employment most certainly will depend on this factor.

Concerning unemployment duration, most studies find that the genuine duration dependence is negative, i.e. the longer the unemployment spell, the smaller the chance of finding a job^11^ (see Serneels, 2002, for an overview). If the study does not disentangle the effect of shortening the maximum benefit period from the negative duration dependence the effect will be biased.

#### 3.4.4 Measures of treatment effect

The treatment effect was measured as the impact on the exit rate from unemployment to employment (measured as a hazard ratio) in all studies except one where it was measured as the difference in mean duration (time to employment). Our main interest was to include studies in a meta‐analysis where hazard ratios and variances were either reported or were calculable from the available data.

The hazard ratio measures the proportional change in hazard rates between unemployed individuals who have a short maximum benefit period and unemployed individuals who have a longer maximum benefit period. The hazard rate is defined as the event rate (in the present context, the event is finding a job) at time *t* conditional on survival (staying unemployed) until time *t* or later. A hazard rate is constructed as follows:^12^

The length of an unemployment spell for an unemployed individual (in the present context the length of stay in the unemployment system until finding a job) is a realization of a continuous random variable *T*. In continuous time, the hazard rate λ(*t*) is defined as:

$$\theta (t)=\underset{\Delta t↓0}{\lim}\frac{\mathrm{Pr}(t\leq T<t+\Delta t|T\geq t)}{\Delta t}=\frac{f(t)}{S(t)}=\frac{f(t)}{1-F(t)},$$

where the cumulative distribution function of *T* is:

F(t)=Pr(T<t)

and the probability density function is:

$$f(t)=\underset{\Delta t↓0}{\lim}\frac{\mathrm{Pr}(t\leq T<t+\Delta t)}{\Delta t}=\frac{dF(t)}{d(t)}.$$

*F(t)* is also known in the survival analysis literature as the failure function and in the present context failure means finding a job. *S(t)* is the survivor function:

S(t)≡Pr(T≥t)=1−F(t);

*t* is the elapsed time since entry to the state (since the individual entered the unemployment system).

Introducing covariates the hazard rate becomes:

$$\theta (t|x(t,s))=\underset{\Delta t↓0}{\lim}\frac{\mathrm{Pr}(t\leq T<t+\Delta t|T\geq t,x(t,s))}{\Delta t},$$

where *x(t,s)* is a vector of personal characteristics that may vary with unemployment duration *(t)* or with calendar time (*s*).

A proportional hazard rate is given by:

$$\theta (t|x)=\theta_{0}(t)*\exp(x\prime \beta ),$$

where *λ_0_* (*t*) is the baseline hazard, exp(*x*′β) is a scale function of the vector *x* of personal characteristics (and a treatment indicator) and β is a vector of estimated parameters.

The baseline hazard is typically not completely specified; often the hazard function is modelled as piecewise constant. Thus, whether the shape of the hazard generally increases or decreases with survival time is left to be estimated from the data, rather than specified a priori.

In the description of the hazard rate it is, so far, implicitly assumed that all relevant differences between individuals can be summarized by observed explanatory variables. But if there are unobservable differences, e.g. motivation and ‘ability’ (in the literature termed unobserved heterogeneity) and these differences are ignored, the estimated parameters will be biased towards zero. It is therefore common to control for both observed factors given by the vector *x* as well as unobserved factors, i.e. unobserved heterogeneity. The hazard rate, including unobserved heterogeneity, is now given by:

$$\theta (t|x,v)=\theta_{0}(t)*\exp(x\prime \beta )v$$

where *v* represents factors unobserved to the researcher and independent of *x*. It is necessary to assume the distribution of *v* has a shape where the right‐hand tail of the distribution is not too fat and whose functional form is summarized in terms of only a few key parameters, in order to estimate those parameters with the data available. The unobserved components are typically assumed to follow a discrete distribution with two (or more) points of support.

The acceptable outcome measurement frequency for calculating hazard ratios in this review was three months or less. A study reporting only outcomes measured on time intervals of more than three months was not included in the meta‐analysis (on secondary outcomes, the study by Barbanchon (2016) provided data on the survival probability in re‐employment within 8 months which was not included in the data synthesis).

As stated in the protocol, Filges et al., 2015a, individual participant data was *not* requested to calculate log hazard ratios as this may introduce bias due to the time span of studies (the time span between the earliest we knew of and the latest is 30 years).

Studies providing estimates of hazard ratios and variances typically base the estimation on the maximum likelihood method^13^. The principle of maximum likelihood is relatively straightforward. The likelihood function, regarded as a function of the parameters of the model, is the joint density of the observations. The maximum likelihood estimator yields a choice of the estimator as the value for the parameter that makes the observed data most probable.

Ignoring unobserved heterogeneity, the contribution to the likelihood for complete observations is given by the conditional density function of t:

$$f(t|x)=\theta (t|x)\exp(-\int_{0}^{t}\theta (s|x)ds)$$

and for censored observations:

$$S(t|x)=\exp(-\int_{0}^{t}\theta (s|x)ds)$$

The likelihood function is:

$$L=f{\left(t|x\right)}^{d}S{\left(t|x\right)}^{1-d}$$

where d=1 for complete observations and d=0 for censored observations. Often it is convenient to maximise the logarithm of the likelihood function rather than the likelihood function and the same results are obtained since *logL* and *L* attain the maximum at the same point.

The log likelihood function to maximize with respect to the parameters of the model is:

$$\log L=d\log f(t|x)+(1-d)\log S(t|x)=d\log\theta (t|x)-\int_{0}^{t}\theta (s|x)ds$$

Introducing unobserved heterogeneity with the random components assumed to follow a discrete distribution with two points of support (*v*_1_, *v*_2_, Pr(*v*_1_)=π_1_, Pr(*v*_2_)=π_2_ the log likelihood function becomes:

$$\log L=\left(d\log\theta (t|x)-\int_{0}^{t}\theta (s|x)ds\right)\pi_{1}+\left(d\log\theta (t|x)-\int_{0}^{t}\theta (s|x)ds\right)\pi_{2}$$

For the continuous outcome mean duration, an effect size with 95% confidence intervals was calculated. Hedges' *g* was used for estimating the SMD and we applied the small *N* correction. Hedges' (adjusted) *g* and its standard error are calculated as (Lipsey & Wilson, 2001:47‐49):

$$g=\left(1-\frac{3}{4N-9}\right)\times \left(\frac{{\bar{X}}_{1}-{\bar{X}}_{2}}{S_{p}}\right),SE_{g}=\sqrt{\frac{N}{n_{1}n_{2}}+\frac{g^{2}}{2N}}$$

where *N*=*n*_1_+*n*_2_ is the total sample size, $\bar{X}$ denotes the (adjusted) mean of a group, and *s_p_* is the pooled standard deviation defined as

$$S_{p}=\sqrt{\frac{(n_{1}-1)s_{1}^{2}+(n_{2}-1)s_{2}^{2}}{\left(n_{1}-1\right)+\left(n_{2}-1\right)}}$$

Here, *s*_1_ and *s*_1_ denotes the standard deviation of the two groups.

Secondary outcomes were measured as the impact on the exit rate from the re‐employment job to unemployment (measured as hazard ratio) and wage ratio.

Software for storing data and statistical analyses were Excel, STATA and RevMan 5.0.

#### 3.4.5 Unit of analysis issues

To account for possible statistical dependencies, we examined a number of issues: whether individuals were randomised in groups (i.e. cluster randomised trials), whether individuals had undergone multiple interventions, whether there were multiple treatment groups, and whether several studies were based on the same data source.

##### Cluster randomised trials

No studies using cluster randomisation were found.

##### Multiple interventions groups and multiple interventions per individuals

Two studies reported separate effect estimates by gender. A synthetic (average) effect size was calculated and used in the analysis to avoid dependence problems. This method provides an unbiased estimate of the mean effect size parameter but overestimates the standard error. Random effects models applied when synthetic effect sizes are involved actually perform better in terms of standard errors than do fixed effects models (Hedges, 2007). However, tests of heterogeneity when synthetic effect sizes are included are rejected less often than nominal.

##### Multiple interventions per individual

There were no studies with multiple interventions per individual used in the analysis.

##### Multiple studies using the same sample of data

Two studies used the same sample of data from Germany and three studies used the same sample of data from Austria, i.e. the studies used administrative register data from the same country covering the same time period. We reviewed all studies, but in the meta‐analysis we only included one estimate of the effect from each sample of data in order to avoid dependencies between the “observations” (i.e. the estimates of the effect) in the meta‐analysis. The choice of which estimates to include was based on our risk of bias assessment of the studies. We chose the estimate from each sample of data from the study that we judged to have the least risk of bias due to confounding.

##### Multiple time points

All studies reported results as an overall effect, either measured as hazard ratios or mean difference in duration.

#### 3.4.6 Dealing with missing data

The reviewers assessed missing data rates in the included studies in accordance with the risk of bias tool used (see section 3.3.3). As stated in the protocol, Filges et al., 2015a, we did not request information from the principal investigators if not enough information was provided to calculate an effect size and standard error due to the time span of studies (the time span between the earliest we know of and the latest is 30 years).

#### 3.4.7 Assessment of heterogeneity

Heterogeneity among primary outcome studies was assessed with Chi‐squared (Q) test, and the I‐squared, and τ‐squared statistics (Higgins, Thompson, Deeks, & Altman, 2003). Any interpretation of the Chi‐squared test was made cautiously on account of its low statistical power. Values of τ‐squared were however, interpreted with caution. The DerSimonian and Laird estimate of τ‐squared is on average overestimated and when the number of studies is small the bias can be substantial (Borenstein, Hedges, Higgins & Rothstein, 2010).

#### 3.4.8 Data synthesis

We carried out our meta‐analysis using hazard ratios^14^. Hazard ratios were log transformed before being analysed. The reason is that ratio summary statistics all have the common features that the lowest value that they can take is 0, that the value 1 corresponds with no intervention effect, and the highest value that a hazard ratio can ever take is infinity. This number scale is not symmetric. The log transformation makes the scale symmetric: the log of 0 is minus infinity, the log of 1 is zero, and the log of infinity is infinity.

All analyses were inverse variance weighted using random effects statistical models that incorporate both the sampling variance and between study variance components into the study level weights. Random effects weighted mean effect sizes were calculated using 95% confidence intervals. Analysis was conducted in RevMan 5. Graphical displays (forest plots) for meta‐analysis performed on ratio scales sometimes use a log scale, as the confidence intervals then appear symmetric. This is however not the case for the software RevMan 5. The graphical displays using hazard ratios and the mean effect size were reported as a hazard ratio. Heterogeneity among primary outcome studies were assessed with Chi‐squared (Q) test, and the I‐squared, and τ‐squared statistics (Higgins, Thompson, Deeks, & Altman, 2003). Any interpretation of the Chi‐squared test was made cautiously on account of its low statistical power.

Studies that were coded with a very high risk of bias (scored 5 on the risk of bias scale) were not included in the data synthesis.

#### 3.4.9 Sensitivity analysis

Sensitivity analysis was used to evaluate whether the pooled effect sizes were robust across components of methodological quality. For methodological quality, we performed sensitivity analyses for the confounding, other bias, and selective reporting items of the risk of bias checklists, respectively. Sensitivity analysis was further used to examine the robustness of conclusions in relation to quality of data (outcome measures based on weekly or monthly data) and using an extension of maximum benefit entitlement to estimate the effect.

## 4 Results

### 4.1 DESCRIPTION OF STUDIES

#### 4.1.1 Results of the search

The search was performed between November 2010 and March 2016.

**Figure 2.1:**
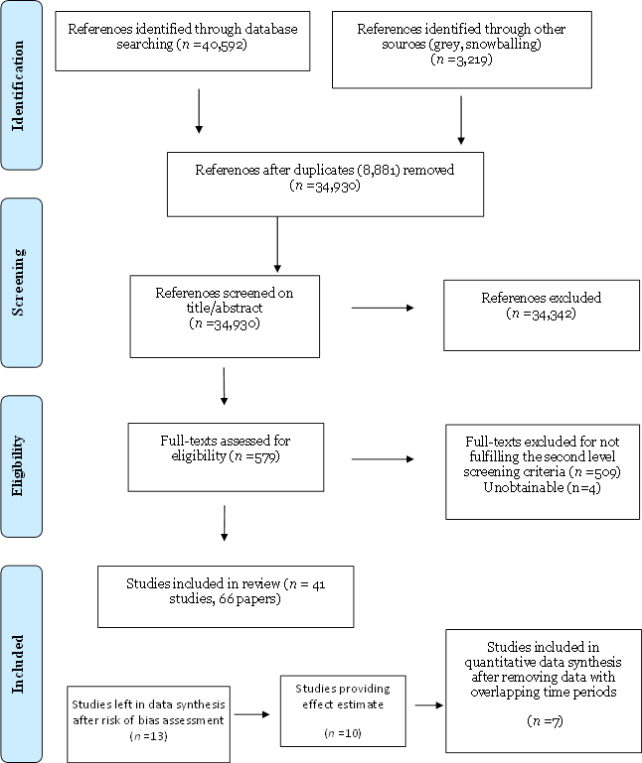


Results are summarised in Figure 2.1 in section 12.2. The total number of potential relevant records was 34,930 after excluding duplicates. All 34,930 records were screened based on title and abstract; 34,342 were excluded for not fulfilling the first level screening criteria and 579 records were ordered for retrieval and screened in full text. Of these, 509 did not fulfil the second level screening criteria and were excluded. Four records were unobtainable despite efforts to locate them through libraries and searches on the internet (see section 9.3). A total of 41 unique studies, reported in 66 papers, were included in the review.

#### 4.1.2 Included studies

The search resulted in a final selection of 41 studies (reported in 66 papers) that met the inclusion criteria for this review. In Table 4.1, we show the total number of studies that met the inclusion criteria for this review. The first column shows the total number of studies grouped by country. The second column shows the number of these studies that were coded with too high risk of bias to be included in the data synthesis. The third column gives the number of studies that did not provide enough data to calculate an effect estimate. The fourth column gives the number of studies that were excluded from the data synthesis due to overlapping samples. The last column gives the total number of studies used in the data synthesis, in total seven studies.

**Table 4.1:** Total number of studies by country

** **	** **	**Reduction due to**			** **
**Country**	**Total**	**Too high risk of bias**	**Do not provide effect estimate** ^1^	**Overlap of data samples** ^2^	**Used in data synthesis** ^3^
Austria	6	1		2	3
Canada	1	1			0
Chile	1	1			0
France	1	0			1
Germany	6	1	2	1	2
Hungary	2	1	1		0
Japan	1	1			0
Netherlands	1	1			0
Poland	1	1			0
Portugal	1	1			0
Slovakia	1	1			0
Slovenia	2	1			1
Spain	4	4			0
Uruguay	1	1			0
US	12	12			0
***Total***	41	28	3	3	7

*Note: The reduction due to too high risk of bias preceded the reduction due to overlap of data sample*.

1 *Or data that enable the calculation of an effect estimate*.

2 *The data samples used are representative for the same population at a given time (see* *Section 3.4.5* *for this methodological issue*).

3 *One study reported on secondary outcomes only*

Of the 41 studies that met the inclusion criteria, 3 did not provide data that permitted the calculation of an effect size (Arntz, Simon & Wilke, 2014; Lee & Wilke, 2009; Micklewright & Nagy, 1995). Two of the three studies, analysing data from Germany, provided a bounds analysis and presented results as figures only (Arntz et al., 2014; Lee & Wilke, 2009). The third study, analysing data from Hungary, also presented results as figures only (Micklewright & Nagy, 1995).

Of the remaining 38 studies, 28 studies were coded with a very high risk of bias (5 on the risk of bias scale) and were therefore not used in the data synthesis (Addison & Portugal, 2008; Ahn & Ugidos‐Olazabal, 1995; Amarante, Arim & Dean, 2013; Arranz, Bulló & Muro, 2008; Belzil, 1995; Bover, Arellano & Bentolila, 2002; Chang, 2010; de Groot & van der Klaauw, 2014; Engberg, 1990; Farber & Valletta, 2015; Farber, Rothstein & Valletta, 2015; Ferrada, 2011; Figura & Barnichon, 2014; Fitzenberger & Wilke, 2010; He, 2013; Hunter, 1990; Landais, 2015; Lubyova & van Ours, 1997; Machikita, Kohara & Sasaki, 2013; Newton & Rosen, 1979; Puhani, 2000; Rebollo‐Sanz & García‐Pérez, 2015; Rothstein, 2011; U.S. Department of Labor, 1995; Valletta, 2014; Van Ours & Vodopivec, 2006; Winter‐Ember, 1998; Wolff, 1997).

Three additional studies (Lalive, 2007; Lalive, Landais & Zweimuller, 2015; Schmieder, von Wachter, Bender, 2016) could not be used in the data synthesis due to overlapping data samples (i.e., the studies used administrative register data from the same country covering the same time period or overlapping time periods; see Section 3.4.5 for this methodological issue). These studies analysed unemployment benefits in Germany and Austria.

After these exclusions from the data synthesis, seven studies remained that could be used in the data synthesis (Barbanchon, 2016; Caliendo, Tatsiramos & Uhlendorff, 2013; Card, Chetty & Weber, 2007; Lalive & Zweimüller, 2004; Nekoi & Weber, 2015; Schmieder, von Wachter, Bender, 2012; Van Ours & Vodopivec, 2008). One of these seven studies, however, only reported on secondary outcomes (Van Ours & Vodopivec, 2008).

For studies with overlapping samples, 2 studies on German data and 3 studies on Austrian data, the choice of which study to use in the data synthesis was based on our risk of bias assessments. The citations for the ten studies that provided effect size estimates and could be used in the data synthesis can be found in Section 9.1.

One of the two German studies (Schmieder, von Wachter & Bender, 2012; Schmieder, von Wachter & Bender, 2016), using data representative of the same population of unemployed at the same time, was deselected as it was judged to have the higher risk of bias due to Other bias (Schmieder, von Wachter & Bender, 2016).

Five studies analysed unemployed in Austria spanning the time period 1981 to 2011^15^ (Card, Chetty & Weber, 2007; Lalive, 2007; Lalive & Zweimüller, 2004; Lalive, Landais & Zweimüller, 2015; Nekoei & Weber, 2015). Although the five studies analysed unemployed in Austria during the same time period (or time periods that overlapped) they did not all analyse the same population, but rather three different sub populations. Three studies could thus be included in the data synthesis. The study by Card et al. (2007) analysed unemployed workers 20‐50 years of age with 1‐5 years of work experience within the last 5 years preceding their unemployment spell and was included in the data synthesis. The study by Nekoei and Weber (2015) analysed workers 38‐42 years of age with more than 6 years of work experience within the last 10 years preceding their unemployment spell and was included in the data synthesis. Three remaining three studies (Lalive, 2007; Lalive & Zweimüller, 2004; Lalive et al., 2015) analysed unemployed workers 45‐54 years of age. Two of these studies further restricted the analysis to workers with a continuous work history during the 25 years preceding their unemployment spell (Lalive, 2007; Lalive & Zweimüller, 2004). Two of the studies (Lalive, 2007 and Lalive et al., 2015) were deselected as they were judged to have a higher risk of bias than Lalive and Zweimüller (2004) due to Confounding.

The detailed characteristics of all 41 studies are provided in Section 14.1.1. A summary of the characteristics of the seven studies that were used in the data syntheses are shown in Table 4.2. These seven studies are the ones on which the conclusions on the effect of maximum duration are based.

**Table 4.2:** Characteristics of studies used in the data synthesis

**Study characteristics**	**Number of studies**
**Country**	
Austria	3
Germany	2
France	1
Slovenia	1
**Analysis period**	
Before 2000	1
After 2000	2
Both before and after 2000	4
**Time interval the outcome measure is based on**	
Weekly	3
Monthly	4
**Type of unemployment benefit**	
Unemployment insurance benefits	7
**Availability of alternative benefits**	
Reported means tested social assistance	7
**Type of data**	
Administrative registers	7
Questionnaire	0
**Considered specific gender or separated by gender**	
Considered only males	1
Separated estimates by gender	2
**Compulsory activation a part of the system**	
Yes	1
Not reported	6
**Labor market conditions**	
Describe labour market conditions	0
**Considered specific age group or experience level**	
Specific age group	4
Specific work experience level	3
**Entitlement**	**Statistics**
Maximum entitlement	Range: 26‐209 weeks
	Average: 75 weeks (SD 58)
Range of individual variation within a study	Range: 9‐179 weeks
	Average: 43 weeks (SD 56)
**Sample size**	
Number of unemployment spells (unemployment periods)	Range: 5,017‐509,355
	Average: 164,870 (SD 184.523)
	Total: 1,154,090

All studies analysed variation in entitlement of unemployment insurance benefits in European countries. One study used data from the 1980s and 1990s. Two studies used data from the 2000s and four studies used data from the periods both before and after 2000. Data were drawn from administrative registers. The sample sizes were generally large; all of the studies used sample sizes of more than 5,000 and in total 1,154,090 spells were used. In three studies the time interval of the outcome measure was weekly and in four studies it was monthly.

One study included only males and two studies provided results separated by gender. Only one of the studies reported whether compulsory labour market activation was part of the unemployment system. All studies reported on the availability of alternative benefits, but only reported that means tested unemployment assistance was available. None of the studies reported the labour market conditions (unemployment rate, vacancy rate and/or labour market tightness^16^).

There was a high degree of variation in maximum entitlement, ranging between 26 and 209 weeks. On average the studies analysed a reduction of 43 weeks in maximum entitlement; the smallest being a reduction of 9 weeks and the largest a reduction of 179 weeks. Four studies were restricted to a specific age group and three studies were restricted to specific work experience levels.

As expected none of the studies were based on randomisation of participants. The central problem in studies without randomisation of participants is the identification of the causal effect of the intervention. The restrictions on age and work experience in the studies were a consequence of the limitations in possible identification strategies. The studies relied on age dependent and/or work experience dependent and/or region dependent variation in benefit rules. The restrictions on age and/or work experience and identification strategy used in the primary studies included in the data synthesis are shown in table 4.3.

**Table 4.3:** Restriction and methods used in the studies used in the data synthesis

**Study**	**Country**	**Range of benefit entitlement**	**Restrictions on age** ^1^	**Restrictions on work experience** ^1^	**Method**
Barbanchon, 2016	France	7‐15 months	50 + aged are excluded	6‐10 months of work	Regression discontinuity design; individual variation in entitlement due to work experience.
Caliendo, Tatsiramos & Uhlendorff, 2013	Germany	12‐18 months	44‐46 years of age.	At least 36 months during the past seven years	Individual variation in entitlement due to age. Regression discontinuity design; the discontinuity is 45 years of age.
Card, Chetty & Weber, 2007	Austria	20‐30 weeks	20‐50 years of age.	Between 1 and 5 years in the past 5 years.	Regression discontinuity design; individual variation in entitlement due to work experience.
Lalive & Zweimüller, 2004	Austria	30‐209 weeks	45‐54 years of age	Work more than 15 years the last 25 years	Difference‐in‐difference‐in‐difference design (age‐ and region‐specific and other time trends). Legislative changes (extension for 50+ aged workers living in regions with a large steel sector)
Nekoei & Weber, 2015	Austria	30‐39 weeks	38‐42 years of age	Work at least 6 years during the last 10 years	Regression discontinuity design. Individual variation in entitlement due to age; the discontinuity is 40 years of age.
Schmieder, von Wachter & Bender, 2012	Germany	12‐18 months	Around age threshold at 42 years of age.	Worked at least 52 months in the last 7 years	Regression discontinuity design. Individual variation in entitlement due to age, threshold at 42 years of age.
Van Ours & Vodopivec, 2008	Slovenia	3‐6 months	None	Between 1 and 5 years of work the last 5 years	Difference‐in‐difference design. Legislative change (reduction) and individual variation in entitlement due to work experience.

1: Only the restrictions relevant for the effect estimate used in the data synthesis is shown

As a consequence of the identification strategies used, none of the studies analysed a representative sample of unemployed workers.

Two studies relied on legislative changes of the maximum entitlement period for specific age groups, work experience levels or regions. One of the two studies analysed an extension of maximum benefit entitlement in Austria (Lalive and Zweimüller, 2004), the other analysed a reduction of maximum benefit entitlement in Slovenia (van Ours and Vodopivec, 2008).

The reform of extended benefit entitlement in Austria, studied in in Lalive and Zweimüller (2004), was enacted to mitigate labour market problems in certain regions and for certain subgroups of workers. The extension was limited to job seekers aged 50 or more, living in certain regions and in effect for a limited time period only (the extension was rolled back after a few years). The specific implementation of the extension ensured that several groups of workers who were not entitled to the extension ‐ yet quite similar to entitled individuals ‐ could be used as control groups. The identification strategy used in Lalive and Zweimüller (2004) accounted for time trends using a difference‐in‐differences‐in‐difference strategy. The authors argue that treated individuals were not subject to idiosyncratic shocks during the observation period. Thus, the policy of extended benefit entitlement could be considered ‘exogenous’^17^ and used to identify the causal effect of the intervention (see Lalive & Zweimüller, 2004, for further details).

The study by van Ours and Vodopivec (2008) relied on a reform of the Slovenian unemployment insurance system in 1998 for identification. Maximum entitlement depended on work experience and the reform reduced the maximum duration of benefits by roughly half for most groups of recipients (except the group with the lowest level of work experience). The reform introduced different variations in potential benefit duration for different groups of unemployed, which, according to the authors, speaks to the credibility of the applied identification strategy. To identify the effect of reducing maximum entitlement, the authors adopted a difference‐in‐difference strategy and compared the probability of entering employment before and after the reform, for those affected by the reform and for those not affected. The reform affected inflows to unemployment around the time of its introduction, increasing inflows just before the introduction and reducing inflows just after. To avoid bias the authors do not consider data covering the 2 months before the introduction of the reform and the 2 months after.

In Caliendo, Tatsiramos & Uhlendorff (2013) and Schmieder, von Wachter & Bender (2012) analyses on German data were based on regression discontinuity designs. The identification strategy used in both studies relied on a sharp discontinuity in the maximum duration of unemployment benefits in Germany; at the age of 45 (used in Caliendo, et al. (2013)) and at the age of 42 (used in Schmieder et al. (2012)). Comparing newly unemployed individuals who were just below the age threshold with newly unemployed individuals just above the age threshold, and accounting for age trends, gives a measure of the effect of maximum duration of benefits on job finding at the cut‐off.

In Nekoei and Weber (2015) and Card et al. (2007) the analyses, relying on Austrian data, were also based on regression discontinuity designs. The identification strategy used in Nekoei and Weber (2015) relied on a sharp discontinuity in the maximum duration of unemployment benefits in Austria at the age of 40. The identification strategy used in Card et al. (2007) relied on a sharp discontinuity in the maximum duration of unemployment benefits in Austria at 36 months of work within the past 5 years before the start of the unemployment spell.

Finally, Barbanchon (2016) relied on a sharp discontinuity in the maximum duration of unemployment benefits in France, where the discontinuity occurred at 8 months of work during the past 5 years before the start of the unemployment spell.

Section 144 provides a further description of all the individual studies (including those without effect estimate and those with very high risk of bias) and a more detailed description of how the maximum durations of unemployment benefits vary.

#### 4.1.3 Excluded studies

In addition to the 41 studies that met the inclusion criteria for this review, 38 studies (reported in 49 papers) appeared relevant but did not meet our inclusion criteria. These studies and the reasons for exclusion are given in Section 9.2 and Section 11.

#### 4.1.4 Studies awaiting classification

Four references were not obtained in full text despite repeated attempts to locate them (see Section 9.3).

### 4.2 RISK OF BIAS IN INCLUDED STUDIES

The detailed risk of bias coding for each of the 41 included studies is shown in Section 14.2. A summary of the risk of bias rating is shown in Table 4.4. Because all included studies used non‐randomised designs, they were all judged to have a high risk of bias on the Sequence generation item and the Allocation Concealment item. The treated group has to know they are treated in order to react to it; therefore, it is not relevant to consider blinding of the participants. Furthermore, the nature of the outcome, exit into employment, is objective and obtained from administrative registers or questionnaires, which were not collected with the aim of analysing changes in the maximum benefit duration. We therefore rated all the studies 4 on the Blinding item. Two studies were rated 5 on the Incomplete outcome data item, both of these studies were also rated 5 on either the Other bias or the Confounding item. Approximately half of the studies did not provide enough information for the Incomplete outcome data item to be rated. No study was rated 5 on the Selective reporting item and almost all were free of any risk of bias on this item. Only five studies had serious issues on the Selective reporting item, leading to a rating of 4 (a detailed description of the reasons can be found in Section 14.2). Ten studies were rated 5 on the Other bias item, nine of these were also rated 5 on the Confounding item. In total 27 studies were rated 5 on the Confounding item.

**Table 4.4:** Risk of bias ‐ distribution of the 41 studies

	Judgment										
**Risk of bias item**	High	Low	1	2	3	4	5	Unclear	Yes	No	Number of studies
Sequence generation	41	0	‐	‐	‐	‐	‐	0	‐	‐	41
Allocation concealment	41	0	‐	‐	‐	‐	‐	0	‐	‐	41
Blinding^1^	‐	‐	0	0	0	41	0	0	‐	‐	41
Incomplete outcome data^1^	‐	‐	3	7	5	2	2	22	‐	‐	41
Free of selective reporting^1^	‐	‐	34	1	1	5	0	0	‐	‐	41
Free of other bias^1^	‐	‐	15	2	6	5	10	3	‐	‐	41
A priori protocol	‐	‐	‐	‐	‐	‐	‐	0	0	41	41
A priori analysis plan	‐	‐	‐	‐	‐	‐	‐	0	0	41	41
Confounding^1^	‐	‐	0	2	2	10	27	0	‐	‐	41

*Notes: 1 The judgment is based on a 5‐point scale, where 1 indicates low risk of bias and 5 indicates high risk of bias.*

Some of the studies judged to be at very high risk of bias on the Confounding item, based the analysis on sharp discontinuities in treatment status at a certain age level and/or work experience level but failed to deliver sufficiently credible arguments in favour of the validity of the identification strategies. The authors did not convincingly argue that the variation used for identification was not subject to selection around the cut off, i.e. insufficient arguments, or no arguments, concerning agents' inability to precisely control the assignment variable near the known cut off were provided.

In general, there was a lack of test of smoothness and density tests reported in the studies to support the validity of the designs chosen. Figures or tests addressing the central assumption behind the validity of the research design, such as of smoothness, should be provided; in the case of the regression discontinuity design, as evidence to support the assumption that all relevant factors (other than treatment) are evolving smoothly with respect to the assignment variable. Likewise figures or tests of density should be provided to assure that the density of the assignment variable is continuous at the discontinuity threshold (the cut off). For further information on smoothness and density tests, see Lee & Lemieux, 2010.

In a number of studies, the main concern was a lack of information in the data sets used. Especially studies using data from the US lacked information on whom among those eligible for unemployment insurance (UI) actually received UI (for example, the UI take up rate is around 50% according to Farber and Valetta (2015)). Further, in most of the studies using data from the US, entitlement (to ordinary UI) was set to the maximum (26 weeks) for all, regardless of the individuals' actual entitlement, as the data set used by the researchers did not include information on individual entitlement.

For details concerning confounding in the individual studies, see Section 14.2.

None of the studies had an *a priori* protocol or an *a priori* analysis plan.

In total 28 studies were given a score of 5 on at least one of the risk of bias items and were therefore not included in the data synthesis.

### 4.3 SYNTHESIS OF RESULTS

The majority of studies reported hazard ratios and variances. One study reported mean difference in duration of non‐employment and one study reported only on secondary outcomes. Two studies reported effect measures separately for men and women. For these two studies, the average effect size was calculated and used in the meta‐analysis to avoid dependence problems. One study analysed an extension of maximum entitlement to unemployment benefits.

#### 4.3.1 Primary outcomes result

Five studies provided effect estimates measured as hazard ratios. A hazard ratio greater than 1 indicates that the treated (with reduced maximum entitlement to unemployment benefits) is favoured. That is, the conditional exit rate from unemployment into employment is higher for persons who have a lower maximum entitlement to unemployment benefits. All reported results indicated a positive effect favouring the treated. The total number of spells used was 1,091,088.

**Figure 4.1:**
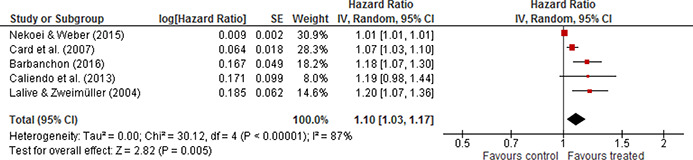
Forest plot, exit to employment, hazard ratio

The weighted average was positive and statistically significant. The random effects weighted mean hazard ratio was 1.10 (95% CI 1.03 to 1.17, p=0.0005). The forest plot is displayed in Figure 4.1. There is some heterogeneity between the studies; the estimated τ^2^ is 0.00 (the more exact value of the estimated τ^2^ is 0.003, Q= 30.12, df=4, p<0.00001) and I^2^ is 87% as displayed in figure 4.1.

The *I^2^* indicates a high degree of heterogeneity; however this appears to be mainly due to the sensitivity of the *I^2^* statistic to the precision of the primary studies effect sizes (Rücker, Schwarzer, Carpenter, & Schumacher, 2008). The value of I^2^ is sensitive to the precision of the primary studies effect sizes, in the sense that the more precisely the primary studies effect sizes are estimated, the higher the values of I^2^, all else equal (Rücker et al., 2008). In this case the estimated between study variance is more informative about the consistency of the evidence. There is some degree of heterogeneity, but as indicated by the value of τ^2^, it may not be of high practical importance.

One study (Schmieder et al., 2012) provided data on the mean difference (and standard deviation) in non‐employment duration. The number of spells used was 45,301. The standardized mean difference in non‐employment duration is ‐0.053 [95% CI ‐0.060, ‐0.046].

Overall, the data synthesis for the effect on the exit rate to work revealed a small and statistically significant effect. The effect favoured the treated in the sense that reducing the maximum entitlement to unemployment benefits increases the exit rate to work. The one study that reported mean difference in non‐employment duration supported this result. Workers experienced shorter periods of non‐employment when the maximum entitlement to unemployment benefits decreased.

#### 4.3.2 Secondary outcomes result

In addition to the primary outcome, we considered secondary outcomes that were relevant to the impact that reducing the maximum entitlement to unemployment benefits can have on re‐employment. Results on the exit rate from re‐employment and wage ratio were provided.

**Figure 4.2:**
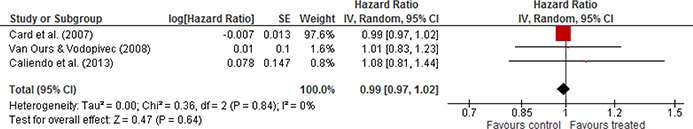
Forest plot, exit from re‐employment, hazard ratio

Three studies (Caliendo et al., 2013; Card et al., 2007 and van Ours & Vodopivec, 2008), provided data on the exit rate from re‐employment. A hazard ratio of less than 1 indicates that the treated (with reduced maximum entitlement to unemployment benefits) is favoured. That is, the conditional exit rate from re‐employment into unemployment is lower for persons who had a lower maximum entitlement to unemployment benefits at the time the job was found. The evidence is inconclusive; one study reported results indicating a negative effect and two studies reported results indicating a positive effect. The total number of spells used was 532,073. Pooled results showed a negative non‐significant effect. The random effects weighted hazard ratio was 0.99 (95% CI 0.97 to 1.02, p=0.64). There were no statistically significant heterogeneity of effects among studies (τ^2^=0.00, Q= 0.36, df=2, p=0.84). Although the p‐value of the Q‐statistic is notoriously underpowered to detect heterogeneity in small meta‐analyses, the estimated τ^2^ is 0.00 and I^2^ is 0%, implying that heterogeneity among these three studies is not present. The forest plot is displayed in Figure 4.2.

Three studies provided data on the log wage ratio in the re‐employment job (Barbanchon, 2016; Card et al., 2007 and Nekoei & Weber, 2015).

The evidence is inconclusive; one study reported results indicating a positive effect and two studies reported results indicating a negative effect.

**Figure 4.3:**
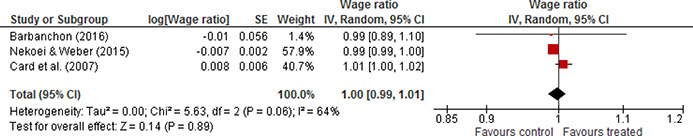
Forest plot, wage ratio in re‐employment job

The weighted average was a wage ratio of 1, indicating no difference between treated and control. The random effects weighted mean wage ratio was 1.00 (95% CI 0.99 to 1.01, p=0.089). The forest plot is displayed in Figure 4.3. There is some heterogeneity between the studies; the estimated τ^2^ is 0.00 (the more exact value of the estimated τ^2^ is 0.0001) and I^2^ is 64% as displayed in figure 4.3.

The *I^2^* indicates some degree of heterogeneity; however, this appears to be mainly due to the sensitivity of the *I^2^* statistic to the precision of the primary studies effect sizes (Rücker, Schwarzer, Carpenter, & Schumacher, 2008). As indicated by the value of τ^2^, it is probably ignorable.

#### 4.3.3 Sensitivity analysis

Sensitivity analyses were planned to evaluate whether the pooled effect sizes were robust across study design and components of methodological quality. Due to the fact that we found no randomised controlled trials, we could not evaluate the impact of study design. For methodological quality, we carried out sensitivity analyses for the Confounding, Other bias, and Selective reporting components of the risk of bias checklists, respectively. We examined the robustness of conclusions when we excluded studies with risk of bias scores of 4 on Confounding, Other bias, and Selective reporting. Sensitivity analysis was further used to examine the robustness of conclusions in relation to the quality of data (outcome measures based on weekly or monthly data collection). Finally, sensitivity analyses were used to examine robustness of conclusion when we removed the study analysing an extension of the maximum entitlement to unemployment benefits.

The results are provided in Table 4.5 and displayed in a forest plot in Section 13.1.

**Table 4.5:** Sensitivity analysis ‐ results

** **	**HR [CI 95%] (Number of studies)**
All studies	1.10 [1.03, 1.17] (5)
*Characteristics of studies* **removed** *from the analysis:*	ES and confidence interval with studies removed
Confounding score of 4	1.12 [1.03, 1.23] (3)
Other bias score of 4	1.14 [1.05, 1.23] (4)
Selective reporting score of 4	1.20 [1.08, 1.33] (2)
Based on monthly data	1.09 [1.02, 1.16] (4)
Analysing an extension	1.08 [1.01, 1.14] (4)

There were no appreciable changes in the results either due to exclusion of studies with scores of 4 on the Confounding, Other bias, or Selective reporting components of the risk of bias checklists. Further, there were no appreciable changes in the results following removal of the study based on monthly data or the study analysing an extension of the maximum entitlement to unemployment benefits.

The overall conclusion that the hazard rate significantly increases when reducing the maximum entitlement to unemployment benefits does not change.

## 5 Discussion

### 5.1 SUMMARY OF MAIN RESULTS

This review focused on the effect of reducing the maximum entitlement to unemployment benefits. The available evidence does suggest that there is an effect, although the effect is small. We found a statistically significant effect of reducing the maximum entitlement to unemployment benefits. The effects were measured by hazard ratios in five studies and one study reported mean difference in non‐employment duration. In the context of hazard ratios (the ratio of two hazard rates), the hazard is the rate within a short time interval at which the unemployed individual finds a job, conditional on staying unemployed. In other words, the probability of finding a job in that short time interval is the hazard rate. The weighted average effect (using the five studies reporting hazard ratios) measured as a hazard ratio is 1.10, which translates into an increase of approximately 10% in the exit rate from unemployment into employment. The effect thus favoured the treated in the sense that reducing the maximum entitlement to unemployment benefits increases the exit rate to work. The one study that reported mean difference in non‐employment duration supported this result.

Interpretation of the result of a 10% increase in the exit rate from unemployment into employment would ideally involve a measure of the average hazard rates for the comparison. However, none of the five studies reported such rates. Some of the studies displayed figures of the average hazard rates over the entire unemployment period. Using these figures, we were able to estimate that the relevant hazard rates (depending on the elapsed duration, all papers reported decreasing hazard rates over the spell) lie in the interval 0.02‐0.14, i.e., the conditional probability of finding a job in a short time interval (a week or a month depending on the unit of analysis in the primary studies) lies between 2% and 14%. Thus, the hazard rates have increased with 10% to the interval 0.022‐0.154, i.e., the conditional probability of finding a job in a short time interval has increased to 2.2‐15.4% solely due to a shorter benefit entitlement period.

The interpretation of a hazard ratio greater than one is that a treated unemployed person who has not yet found a job by a certain time has a higher chance of finding a job at the next point in time compared to someone in the control group.

There is an alternative interpretation of the hazard ratio that may be intuitively easier to understand. The hazard ratio is equivalent to the odds that an individual in the group with the higher hazard reaches the endpoint (finds a job) first.

Stated another way, for any pair of unemployed people, one from the treatment group and one from the control group, the hazard ratio is the odds that the time to find a job is less in the unemployed from the treatment group than in the unemployed from the control group. The probability of finding a job first (P) can easily be derived from the odds or hazard ratio (HR) of finding a job first, which is the probability of finding a job first divided by the probability of not finding a job first: HR=odds= *P*/(1‐ *P*); *P=* HR/(1+ HR) (Spotswood et al. 2004). A hazard ratio of 1.10 therefore corresponds to a 52% chance of the treated unemployed person finding a job first. The lower and upper 95% confidence interval corresponds to a 51‐ 54% chance of the treated unemployed person finding a job first.

Concerning secondary outcomes, we analysed the effect of reducing the maximum entitlement to unemployment benefits on the subsequent exit rate from the re‐employment job and the wage ratio in the re‐employment job. Only three studies could be used in each of these analyses. The overall impact on the exit rate from the re‐employment job of shortening the maximum duration of unemployment benefit entitlement, obtained using hazard ratios, was 0.99 and the overall wage ratio was 1.00. There is a lack of evidence to support the hypothesis that reducing the maximum entitlement to unemployment benefits has an impact on the quality of the job, measured as the exit rate of re‐employment and wage ratio.

### 5.2 OVERALL COMPLETENESS AND APPLICABILITY OF EVIDENCE

In this review, we included in total seven studies in the data synthesis. This number is relatively low compared to the large number of studies (41) meeting the inclusion criteria. The reduction was caused by three different factors. Three of the 41 studies did not report effect estimates or provide data that would allow the calculation of an effect size. Twenty‐eight studies were judged to have a very high risk of bias (5 on the scale) and, in accordance with the protocol, we excluded these from the data synthesis on the basis that they would be more likely to mislead than inform on the size of the effect of the intervention. Three further studies were excluded because of overlapping data samples.

If all the 41 studies had provided an effect estimate with lower risk of bias, the final list of useable studies in the data synthesis would have been larger^18^ which, in turn, would have provided a more robust literature on which to base conclusions.

In total, 15 countries were represented by the 41 studies meeting the inclusion criteria. The seven studies used in the data synthesis covered Austria, France, Germany and Slovenia (four countries, all European).

The geographical coverage thus became narrower as studies from the US, Netherlands, Canada, Portugal, Spain, Chile, Hungary, Japan, Uruguay, Slovakia and Poland could not be used in the data synthesis. Furthermore, all the studies used in the data synthesis were restricted to a specific (narrow) age group and/or were restricted to specific (narrow) work experience levels. These restrictions of representativeness constitute a clear limitation in terms of the generalizability of the results of the review.

The narrow geographical coverage and the restrictions of participants (to specific age and/or work experience levels) may limit the applicability of the evidence and it may be difficult to translate results to expected effects in other settings. The applicability of the evidence would be greater if an effect was found across more different labour markets, such as the US labour market (liberalistic) and e.g. the Scandinavian labour markets (comprehensive welfare state institutions) and across different characteristics of the of unemployed.

It was not possible to examine the impact of a reduction of the maximum entitlement to unemployment benefits of the following moderators: gender, age, education, type of unemployment benefit, whether alternative benefits were available, and if compulsory activation was part of the system or labour market conditions. These factors are all potential moderators of the effect that policy‐makers must take into account in accordance with the country's specific institutional setting in order to assess the possibility of generalizing the synthesized result to the specific population for whom a reform of existing entitlement rules is under consideration.

In attempt to obtain a clearer picture of the effect of a reduction of the maximum entitlement to unemployment benefits on the quality of the job obtained, we analysed the subsequent exit rate from re‐employment and the re‐employment wage ratio as secondary outcomes. Only three studies were eligible for analysis of each of these outcomes. The small number of studies reporting these outcomes makes us reluctant to draw a conclusion.

### 5.3 QUALITY OF THE EVIDENCE

All studies used non‐randomised designs. Overall the risk of bias in the majority of included studies was high. Twenty‐eight studies were judged to be at very high risk of bias.

Some of the studies judged to be at very high risk of bias based the analysis on a regression discontinuity design relying on sharp discontinuities in age and/or work experience level. In general, these studies did not provide arguments concerning agents' inability to precisely control the assignment variable near the known cut off and there was a lack of test of smoothness and density tests reported in the studies to support the designs chosen. Another issue, especially concerning US studies was the lack of relevant information in the data sets used. The data sets used in many US studies did not have information on whom among the UI eligible actually received UI and who were entitled to maximum duration.

The risk of bias was examined using a tool for assessing risk of bias incorporating non‐randomised studies. We attempted to enhance the quality of the evidence in this review by excluding studies judged to be at very high risk of bias from the data synthesis, using this tool. We believe this process excluded from the data synthesis those studies that were more likely to mislead than inform.

Furthermore, we performed a number of sensitivity analyses to check whether the obtained result is robust across methodological quality, data quality and direction of change to entitlement. The overall conclusion did not change.

To check the robustness across methodological quality, the studies with relatively high risk of bias (score of 4) in Confounding, Other bias, and Selective reporting, respectively, were excluded from the analysis. To check the robustness across data quality, the one study with an estimate on monthly data was removed. In addition, the one study analysing an extension of maximum unemployment benefit entitlement was removed.

The overall conclusion that the hazard rate significantly increases when the maximum entitlement to unemployment benefits is reduced did not change. Due to the low number of studies, it was not possible to perform sensitivity analyses for secondary outcomes.

There was overall consistency in the direction of effects on the exit rate to employment in that all effects favoured the unemployed with the shortest maximum entitlement to unemployment benefits in finding a job. There was some degree of heterogeneity between studies, but as indicated by the very low value of the estimated between study variance (τ^2^), it may not be of high practical importance.

### 5.4 LIMITATIONS AND POTENTIAL BIASES IN THE REVIEW PROCESS

We believe that all the publicly available studies on the effect of reducing the maximum entitlement to unemployment benefits on employment up to the censor date were identified during the review process. However, four references were not obtained in full text.

We were unable to comment on the possibility of publication bias because there were insufficient studies included in the meta‐analysis for the construction of funnel plots. Thus, it may be possible there are still some missing studies.

We believe that there are no other potential biases in the review process as two members of the review team^19^ (JKS, UHP) independently coded the included studies. Any disagreements were resolved by discussion. Further, decisions about inclusion of studies and assessment of study quality were made by two review authors (ABJ, TF) independently and minor disagreements resolved by discussion. Numeric data extraction was made by one review author (TF) and was checked by a second review author (ABJ).

### 5.5 AGREEMENTS AND DISAGREEMENTS WITH OTHER STUDIES OR REVIEWS

As this is the first systematic review of the literature on the effects on job finding rates of changing the maximum unemployment benefit duration no directly comparable literature exists.

An early related contribution is Krueger & Meyer (2002) summarising evidence on the labour supply effects of social insurance programmes, including unemployment insurance benefits. The authors, however, do not specifically draw conclusions regarding the size of effects of changes to the maximum unemployment benefit duration, but merely state that “the programs tend to increase the length of time employees spend out of work” (p. 2327).

Closest to our work are two recent contributions by Tatsiramos & van Ours (2014) and by Schmieder & von Wachter (2016). However, none of these reviews are systematic in their search of relevant literature and neither do any of these reviews distinguish between destinations (employment or out of unemployment). Tatsiramos & van Ours (2014) presents an overview of the results from six recent studies on the effects of the potential benefit duration, while Schmieder & von Wachter (2016) present a selected sample of five US studies and eight European studies. Different from our approach, the overviews in both Tatsiramos & van Ours (2014) and Schmieder & von Wachter include estimates of the effect of the potential benefit duration on unemployment duration, which does not imply a similar effect on job finding. The effect of the potential benefit duration on e.g. non‐employment duration need not be the same as the effect on unemployment duration (see e.g. Schmieder & von Wachter (2016) for further discussion). In fact, studies reporting both estimates, typically report substantially larger estimates of the effect on unemployment duration than of the effect on non‐employment duration (see e.g. Lalive (2007), Schmieder et al. (2012) and Barbanchon (2016)).

Importantly, our systematic review also differs from the reviews by Tatsiramos & van Ours (2014) and Schmieder & von Wachter (2016) by not including in the data synthesis estimates from studies that were judged (transparently) to have too high risk of bias and more likely to mislead than inform on the effect size of the intervention.

Besides not relying on a systematic search approach, the contributions by Tatsiramos & van Ours (2014) and by Schmieder & von Wachter (2016), thus, are not directly comparable to our review in two important respects concerning the studies forming the basis of the conclusions. Tatsiramos & van Ours (2014) and Schmieder & von Wachter (2016) include in their data synthesizes studies, which we have excluded from our review due to not considering the effect on job finding rates. Both studies also include studies which we have excluded from our data synthesis due to too high risk of bias.

In addition, although both reviews present estimates from all the included studies, it is not fully transparent how they synthesized them in order to reach the conclusions.

Schmieder & von Wachter (2016) report the median marginal effect on duration, the range and the mean marginal effect on duration (after two outliers at the top and bottom have been removed, p. 15) and conclude that recent studies (US and European) point to an only moderate negative labor supply effect of the potential benefit duration (p. 14).

Tatsiramos & van Ours (2014) state that: “An extension of potential benefit duration leads to an increase in actual unemployment duration of about 20% of the original benefit duration extension” (p. 299) and conclude that the effects on unemployment duration of changes to potential benefit duration are substantial. Note that this conclusion is based on an extension of potential benefit duration and it is not evident that they should be symmetric to effects of reductions.

The available evidence analysed in our systematic review also suggests an effect of changing the maximum entitlement to unemployment benefits on job finding rates, although the size of the effect is small. As such, the conclusion from this systematic review regarding the magnitude of the effect of the intervention may seem inconsistent with the conclusion in the review in Tatsiramos & van Ours (2014). However, it should be kept in mind that the apparently different conclusions concerning the magnitude of the effects are obtained based on very different inclusion criteria concerning outcomes and substantially different approaches and statistical methods.

## 6 Authors' conclusions

### 6.1 IMPLICATIONS FOR PRACTICE AND POLICY

Search theory suggests that shortening the benefit eligibility period may reduce the share of long and unproductive job searches as a shorter maximum eligibility period will tend to accelerate job searching from the beginning of the unemployment spell.

In this review, we have found evidence that a reduction in the maximum entitlement to unemployment benefits results in an increase in job finding. Thus, the theoretical suggestion of an effect of a shorter benefit period on accelerated job finding rates has been confirmed empirically, although the impact is small.

The effect of shortening the maximum entitlement to unemployment benefits was measured by hazard ratios. The overall impact of shortening the maximum entitlement to unemployment benefits corresponds to a 52% chance of the treated unemployed person finding a job first.

Overall, shortening the maximum entitlement to unemployment benefits displays a limited potential to alter the job finding rates of the affected unemployed individuals.

Whether the increased job finding rate implies a decrease in the overall unemployment level depends on whether it is caused mostly by an increase in search intensity or a decrease in reservation wages. If increases in the job finding rates are explained by decreases in reservation wages, those who have a shorter entitlement to unemployment benefits might accept jobs that do not match their qualifications, and from which they are more likely to quit in the future. If the increased job finding rates are explained by increases in the search effort, there is no reason to expect shortening the entitlement period of unemployment benefits forces unemployed individuals to find jobs that do not match their qualifications.

We found three studies that could be used for analysis of the exit rate from the re‐employment job and three studies that could be used for analysis of the wage ratio in the re‐employment job. Based on this low number of studies, we found no evidence to support the hypothesis that shortening the maximum entitlement to unemployment benefits has an impact on the quality of the job in terms of the exit rate from the re‐employment job or the wage in the re‐employment job. Whether the unemployed workers who are affected may actually be worse off, in the sense that they accept “worse” jobs, has not yet been fully investigated.

It was not possible to examine a number of factors which we have reasons to expect have an impact on the magnitude of the effect. Knowledge of whether the effect depends on labour market conditions and benefit system factors such as availability of alternative benefits, and compulsory activation may be crucial to policy‐makers. The factors are all potential moderators of the effect that policy‐makers need to attempt to assess in relation to the context of their country. These factors could possibly have been investigated with more studies from different labour markets and with a sufficiently low risk of bias. The results of this review, however, conclude that across a (small) number of countries there is a small positive effect of shortening the maximum potential benefit period on job finding rates.

### 6.2 IMPLICATIONS FOR RESEARCH

In this review we found evidence that reducing the maximum entitlement to unemployment benefits results in an increased probability of finding work faster, although the impact is small.

By excluding from the data synthesis studies judged to be at very high risk of bias, this review aimed to enhance the quality of the evidence on the effects of reducing unemployment benefit duration. We believe this process excluded those studies that are more likely to mislead than inform on the true effect sizes. Overall the risk of bias in the studies included in the review was high. Many of the available studies were judged to be at very high risk of bias. Twenty‐five studies were given a score of 5 on the Confounding item, corresponding to a risk of bias so high that the findings should not be considered in the data synthesis. Of the remaining 13 studies, three were given a score of 5 on the Other bias item, corresponding to a risk of bias so high that the findings should not be considered in the data synthesis.

Some of the studies were judged to be at very high risk of bias, based the analysis of a regression discontinuity design relying on sharp discontinuities in age and/or work experience level in the Netherlands, Canada, Portugal, Spain, Chile, Hungary, Japan, Uruguay, Slovakia and Poland (and some studies in addition accounted for time trends using a difference‐in‐difference approach). These studies, however, failed to deliver convincing arguments that the identification strategies were not subject to too high risk of selection around cut off. In general, the authors using regression discontinuity designs did not provide arguments concerning agents' inability to precisely control the assignment variable near the known cut off and there was a lack of test of smoothness and density tests reported in the studies to support the designs chosen. Figures or tests of smoothness should be provided to support the assumption that all relevant factors (other than the treatment) are evolving smoothly with respect to the assignment variable and, likewise, figures or tests of density should be provided to assure that the density of the assignment variable is continuous at the discontinuity threshold.

Further, the main concern in a number of studies was the lack of information in the data sets used. In particular, studies using data from the US lacked information on whom among those eligible for unemployment insurance (UI) actually received UI (for example, the UI take up rate is around 50% according to for example Farber and Valetta (2015)). Further, in most of the studies using data from the US, entitlement (to ordinary UI) was set to the maximum (26 weeks) regardless of working history, as the data set used by the researchers did not include information on work history.

As studies from the US, the Netherlands, Canada, Portugal, Spain, Chile, Hungary, Japan, Uruguay, Slovakia and Poland could not be used in the data synthesis, the geographical coverage of the evidence of the effects of reducing the unemployment benefit duration became rather narrow, covering only four countries, all of which were European.

The planned examination of potential moderators of the effect, such as gender, age and labour market conditions, was not possible due to the low number of studies in the data synthesis. If effect sizes from all the countries represented in the review had been useable in the data synthesis, additional valuable information about the heterogeneous effects of reducing the maximum duration of entitlement to unemployment benefits may have resulted.

These considerations point to the need for future studies that more thoroughly discuss the identifying assumptions and justify their choice of method by considering and reporting all relevant data and tests. Further, future studies should rely on data where all relevant information is available, in particular rely on data were there is no lack of information on who among the unemployment benefit eligible individuals actually received unemployment benefits, and who among the unemployment benefit eligible individuals were entitled to the maximum duration.

The quality of the jobs obtained, in terms of duration and income, could not be fully investigated due to limitations in the number of studies reporting such outcomes.

Further research should be directed at the possible side effects, in particular whether the unemployed leave unemployment due to a higher acceptance of low‐paid employment and, in particular, whether job transitions are caused by increased search effort.

## 7 Acknowledgements

We thank members of the review team at SFI Campbell, the research assistants Julie Kaas Seerup, Ulrik Højmark Pedersen and Bjørn Christian Viinholt Nielsen, for their invaluable help.

We would like to thank Dr. B. C. Reeves from the Cochrane Non‐Randomised Studies Methods Group for materials and training regarding the assessment of risk of bias.

The review authors are responsible for any remaining errors.

## 8 Methods not implemented

### 8.1.1 Assessment of reporting bias

We were unable to comment on the possibility of publication bias because there were insufficient studies for the construction of funnel plots.

### 8.1.2 Moderator analysis and investigation of heterogeneity

We planned to investigate the following factors with the aim of explaining observed heterogeneity: Study‐level summaries of participant characteristics (e.g. studies considering a specific age group, gender or educational level or studies where separate effects for men/women, young/old or low/high educational level are available), labour market conditions (good/bad), type of unemployment benefit (UI or SA/UA), whether alternative benefits are available, and if compulsory activation is part of the system.

There were, however, insufficient studies for moderator analysis to be performed.

## 10 Information about this review

### 10.1 REVIEW AUTHORS

**Lead review author:**

The lead author is the person who develops and co‐ordinates the review team, discusses and assigns roles for individual members of the review team, liaises with the editorial base and takes responsibility for the on‐going updates of the review.

**Table:** 

Name:	Trine Filges
Title:	Senior Researcher
Affiliation:	SFI‐Campbell
Address:	Herluf Trollesgade 11
City, State, Province or County:	Copenhagen
Postal Code:	1052
Country:	Denmark
Phone:	45 33480926
Email:	tif@vive.dk
**Co‐author(s):**	** **
Name:	Anders Bruun Jonassen
Title:	Researcher
Affiliation:	The ROCKWOOL Foundation
Address:	Sølvgade 10, 2. tv.
City, State, Province or County:	Copenhagen
Postal Code:	1307
Country:	Denmark
Phone:	45 33 34 48 00
Email:	abj@rff.dk
Name:	Anne Marie Klint Jørgensen
Title:	Librarian/Information Specialist
Affiliation:	SFI‐Campbell
Address:	Herluf Trollesgade 11
City, State, Province or County:	Copenhagen
Postal Code:	1052
Country:	Denmark
Phone:	45 33480868
Email:	amk@sfi.dk

### 10.2 ROLES AND RESPONSIBILITIES

Please give brief description of content and methodological expertise within the review team. The recommended optimal review team composition includes at least one person on the review team who has content expertise, at least one person who has methodological expertise and at least one person who has statistical expertise. It is also recommended to have one person with information retrieval expertise.

Who is responsible for the below areas? Please list their names:

- Content: Trine Filges and Anders Bruun Jonassen
- Systematic review methods: Trine Filges
- Statistical analysis: Trine Filges and Anders Bruun Jonassen
- Information retrieval: Anne Marie Klint Jørgensen

### 10.3 SOURCES OF SUPPORT

**Internal funding:** SFI‐Campbell

**External funding:** None

### 10.4 DECLARATIONS OF INTEREST

None known

### 10.5 PLANS FOR UPDATING THE REVIEW

Trine Filges will be responsible for updating the review every second year when completed.

### 10.6 AUTHOR DECLARATION

**Authors' responsibilities**

By completing this form, you accept responsibility for maintaining the review in light of new evidence, comments and criticisms, and other developments, and updating the review at least once every five years, or, if requested, transferring responsibility for maintaining the review to others as agreed with the Coordinating Group. If an update is not submitted according to agreed plans, or if we are unable to contact you for an extended period, the relevant Coordinating Group has the right to propose the update to alternative authors.

**Publication in the Campbell Library**

The Campbell Collaboration places no restrictions on publication of the findings of a Campbell systematic review in a more abbreviated form as a journal article either before or after the publication of the monograph version in *Campbell Systematic Reviews*. Some journals, however, have restrictions that preclude publication of findings that have been, or will be, reported elsewhere, and authors considering publication in such a journal should be aware of possible conflict with publication of the monograph version in *Campbell Systematic Reviews*. Publication in a journal after publication or in press status in *Campbell Systematic Reviews* should acknowledge the Campbell version and include a citation to it. Note that systematic reviews published in *Campbell Systematic Reviews* and co‐registered with the Cochrane Collaboration may have additional requirements or restrictions for co‐publication. Review authors accept responsibility for meeting any co‐publication requirements.

**I understand the commitment required to update a Campbell review, and agree to publish in the Campbell Library. Signed on behalf of the authors**:

**Table:** 

**Form completed by: Trine Filges**	**Date:** January 23, 2018

## 11 Excluded studies

**Table:** 

**Study**	**Reason for exclusion**
Alba‐Ramírez (1998)	Does not analyse maximum duration entitlement. Only considers receiving benefits or not.
Arranz & Muro (2004)	Does not consider changes to maximum duration entitlement, only time to exhaustion.
van Audenrode & Storer (1992)	Only considers differences between eligible receivers/non receivers of unemployment benefits.
Barron & Wesley (1981)	Does not analyse maximum duration entitlement. Only considers receiving benefits or not.
Bennmarker, Carling & Holmlund (2007)	Only changes to replacement rates analysed.
Bennmarker, Skans & Vikman (2013)	Soft restriction, reduction of duration of passive benefits, participation in active programs (with unchanged benefit level) qualifies for another period of passive benefits.
Burgess & Kingston (1981)	Analyses effect on compensated unemployment duration only and not total time spend in unemployment.
Centeno & Novo (2014)	Does not separate exits to employment and exits to other destinations than employment.
Chetty (2008)	Considers variation in benefit levels between US states, not variation in potential unemployment benefit duration.
Fujita (2011)	No effect of changes in maximum duration estimated, only differences in exit rates between a period where extended benefits were available/not available and further separated in duration intervals (monthly).
Gaure, Røed & Westlie (2008)	Analyses a reform that is not primarily a change in the overall length of the maximum UI duration, but rather a soft duration constraint and a mix of other changes.
Gritz & MaCurdy (1992)	Do not separate exhaustion effects from max entitlement effects.
Grossman (1989)	Data on the number of weeks of unemployment compensation received, not on the number of weeks unemployed.
Ham & Rea (1987)	Analyses effect of remaining entitlement.
Holen (1977)	Data on the number of weeks of unemployment compensation received, not on the number of weeks unemployed.
Hunt (1995)	Does not consider individual differences in maximum duration but differences between (large) age groups: Control group (16‐41 years of age) has maximum benefit duration between 4‐12 months dependent on age and tenure, treated (44‐48 years of age) has maximum benefit duration between 4‐22 months dependent on tenure.
Lalive (2008)	Results for job finding only reported in footnote (and with no standard errors) Two working papers do not consider job finding either, only all destinations after exit from unemployment.
Lalive, van Ours & Zweimuller (2011)	Does not consider, or separate transitions to job from other transitions.
Lalive, van Ours & Zweimüller (2006)	14 percent of spells end in non‐job destinations and transitions to job not analysed separately.
Lauringson (2011)	Estimates effect of covariates separately for those with short and long entitlement and shows baseline separately too.
Leigh (1086)	Analyses replacement rate (not including zero as only unemployment insurance benefit recipients included) effects only
Lindner (2015)	Analyses changes to replacement rate; from flat rate to a decrease after 90 days. Maximum duration is unchanged.
Gritz & MaCurdy (1992)	Does not separate exhaustion effects from max entitlement effects.
Meyer (1990)	Does not separate exits to employment from exits to other destinations than employment.
Moffitt & Nicholson (1982)	Only estimates effect of extended benefits on exhaustees.
Maani (1989)	No limit on benefit eligibility duration.
Narendranathan, Nickell & Stern (1985)	Analyses effect of income while unemployed, not entitlement and duration.
Pérez (2003)	Does not consider changes to entitlement, only benefits or not and do not mention a time limit on benefits.
Poterba et. Al (1995)	Only replacement rate effects are analysed.
Roed & Westlie (2012)	Analyses a reform that is not primarily a change in the overall length of the maximum UI duration. It is a soft duration constraint and a mix of other changes.
Rogers (1998)	Analyses extended benefits in the week the extension becomes available.
Schmieder, von Wachter & Bender (2012)	Analyses the sum of unemployment spells over five years (recurrent unemployment spells over five years).
Steiner (2001)	About negative duration dependence and not changes in benefit duration.
Moffitt (1995)	Data set 1) No exit to job, only exit from UI. Data set 3) Unemployment period not defined in a proper way (p. 75). Data set 4) Dependent variable is duration of receiving UI.
Vodopivec, Laporsek, Dolenc & Vodopivec (20)15	Does not analyse potential unemployment benefit duration
Wunnava & Henley (1987)	Does not consider changes to entitlement, only ‘exhaustion effect’ on average duration.
Wunnava & Mehdi (1994)	Unclear if exit is to job. Examines insured unemployment rates and the duration of this unemployment period, suggesting not only exits to job.

## 12 Appendices

### 12.1 SEARCH DOCUMENTATION

**SocIndex.**

Search string from 2015‐2016 update. Same search string has been used from original search (2011).

**Table:** 

**Search**	**Terms**	**Results**
S10	S5 AND S6 AND S7 AND S8	103
S8	S3 OR S4	465,602
S7	S1 OR S2	97,246
S6	TI ((Expiration* OR Lapse* OR Expiry OR Termination* OR Duration* OR Generosity OR Change OR Entitlement* OR Length* OR Extend* OR Extension OR Exhaust* OR exit*)) OR AB ((Expiration* OR Lapse* OR Expiry OR Termination* OR Duration* OR Generosity OR Change OR Entitlement* OR Length* OR Extend* OR Extension OR Exhaust* OR exit*))	259,662
S5	TI ((effect* OR threat* OR incentive* OR disincentive* OR impact* OR motivat*)) OR AB ((effect* OR threat* OR incentive* OR disincentive* OR impact* OR motivat*))	449,101
S4	TI (Employ* OR Job* OR work* OR (un‐employ* or unemploy*) OR (re‐employ* or reemploy*)) OR AB (Employ* OR Job* OR work* OR (un‐employ* or unemploy*) OR (re‐employ* or reemploy*))	465,564
S3	(DE “EMPLOYABILITY”)	365
S2	TI ((welfare N1 payment*) OR (welfare N1 recipient*) OR (welfare N1 support*) OR (economic N1 support*) OR (Social N1 support) OR (public N1 assistance*) OR (public N1 support*) OR (financial N1 support*) OR (welfare N1 service*) OR (direct* N1 payment*) OR (general N1 assistance) OR (cash N1 assistance) OR (income N1 assistance) OR benefit* OR (social N1 assistance*) OR (social N1 securit*) OR (social N1 welfare) OR (social N1 allowance*) OR (insurance N1 benefit*) OR (social N1 benefit*) OR (welfare N1 benefit*) OR TANF OR insurance)) OR AB ((welfare N1 payment*) OR (welfare N1 recipient*) OR (welfare N1 support*) OR (economic N1 support*) OR (Social N1 support) OR (public N1 assistance*) OR (public N1 support*) OR (financial N1 support*) OR (welfare N1 service*) OR (direct* N1 payment*) OR (general N1 assistance) OR (cash N1 assistance) OR (income N1 assistance) OR benefit* OR (social N1 assistance*) OR (social N1 securit*) OR (social N1 welfare) OR (social N1 allowance*) OR (insurance N1 benefit*) OR (social N1 benefit*) OR (welfare N1 benefit*) OR TANF OR insurance))	95,112
S1	((DE “Social Security”) OR (DE “WELFARE recipients”)	8,384

**PsycInfo.**

Search string from 2015‐2016 update. Same search string has been used from original search (2011).

**Table:** 

**#**	**Query**	**Results**
**S10**	S5 AND S6 AND S7 AND S8	418
**S9**	S5 AND S6 AND S7 AND S8	4,048
**S8**	S3 OR S4	740,705
**S7**	S1 OR S2	194,157
**S6**	TI ((Expiration* OR Lapse* OR Expiry OR Termination* OR Duration* OR Generosity OR Change OR Entitlement* OR Length* OR Extend* OR Extension OR Exhaust* OR exit*)) OR AB ((Expiration* OR Lapse* OR Expiry OR Termination* OR Duration* OR Generosity OR Change OR Entitlement* OR Length* OR Extend* OR Extension OR Exhaust* OR exit*))	541,763
**S5**	TI (effect* OR threat* OR incentive* OR disincentive* OR impact* OR motivat*)) OR AB (effect* OR threat* OR incentive* OR disincentive* OR impact* OR motivat*))	1,133,790
**S4**	TI ((Employ* OR Job* OR work* OR (un‐employ* or unemploy*) OR(re‐employ* or reemploy*)) OR AB ((Employ* OR Job* OR work* OR (un‐employ* or unemploy*) OR(re‐employ* or reemploy*))	740,643
**S3**	DE “EMPLOYABILITY”	1,116
**S2**	TI ((welfare N1 payment*) OR (welfare N1 recipient*) OR (welfare N1 support*) OR (economic N1 support*) OR (Social N1 support) OR (public N1 assistance*) OR (public N1 support*) OR (financial N1 support*) OR (welfare N1 service*) OR (direct* N1 payment*) OR (general N1 assistance) OR (cash N1 assistance) OR (income N1 assistance) OR benefit* OR (social N1 assistance*) OR (social N1 securit*) OR (social N1 welfare) OR (social N1 allowance*) OR (insurance N1 benefit*) OR (social N1 benefit*) OR (welfare N1 benefit*) OR TANF OR insurance)) OR AB ((welfare N1 payment*) OR (welfare N1 recipient*) OR (welfare N1 support*) OR (economic N1 support*) OR (Social N1 support) OR (public N1 assistance*) OR (public N1 support*) OR (financial N1 support*) OR (welfare N1 service*) OR (direct* N1 payment*) OR (general N1 assistance) OR (cash N1 assistance) OR (income N1 assistance) OR benefit* OR (social N1 assistance*) OR (social N1 securit*) OR (social N1 welfare) OR (social N1 allowance*) OR (insurance N1 benefit*) OR (social N1 benefit*) OR (welfare N1 benefit*) OR TANF OR insurance))	193,972
**S1**	((DE “Social Security”) OR (DE “WELFARE recipients”)	826

**Business Source Complete.**

Search string from 2015‐2016 update. Same search string has been used from original search (2011).

**Table:** 

**Search**	**Search Terms**	**Results**
S10	S5 AND S6 AND S7 AND S8	(88)
S9	S5 AND S6 AND S7 AND S8	(1,231)
S8	S3 OR S4	(1,818,954)
S7	S1 OR S2	(945,014)
S6	TI ((Expir*) OR (Lapse) OR (Terminat*) OR (Duration) OR (Generosity) OR (Change OR changes) OR (Entitlement) OR (Length) OR (Extend*) OR (Extension) OR (Exhaust*) OR (exit))	(180,509)
S5	TI (((effect*) OR (threat*) OR (incentive*) OR (disincentive*) OR (impact*) OR (motivat*))) OR AB (((effect*) OR (threat*) OR (incentive*) OR (disincentive*) OR (impact*) OR (motivat*)))	(1,625,402)
S4	TI ((Employ*) OR (Job*) OR (work*) OR (un‐employ* or unemploy*) OR (re‐employ* or reemploy*)) OR AB ((Employ*) OR (Job*) OR (work*) OR (un‐employ* or unemploy*) OR (re‐employ* or reemploy*))	(1,818,863)
S3	(DE “EMPLOYABILITY”)	(970)
S2	TI ((welfare w1 recipient*) OR (welfare w1 support*) OR (economic w1 support*) OR (public w1 assistance*) OR (welfare w1 payment*) OR (public w1 support*) OR (financial w1 support*) OR (welfare w1 service*) OR (direct* w1 payment*) OR (general w1 assistance) OR (Social w1 Support) OR (cash w1 assistance) OR (income w1 assistance) OR (benefit*) OR (social w1 assistance*) OR (social w1 securit*) OR (social w1 welfare) OR (social w1 allowance*) OR (insurance w1 benefit*) OR (social w1 benefit*)) OR AB (welfare w1 recipient*) OR (welfare w1 support*) OR (economic w1 support*) OR (public w1 assistance*) OR (welfare w1 payment*) OR (public w1 support*) OR (financial w1 support*) OR (welfare w1 service*) OR (direct* w1 payment*) OR (general w1 assistance) OR (Social w1 Support) OR (cash w1 assistance) OR (income w1 assistance) OR (benefit*) OR (social w1 assistance*) OR (social w1 securit*) OR (social w1 welfare) OR (social w1 allowance*) OR (insurance w1 benefit*) OR (social w1 benefit*) OR SU (welfare w1 recipient*) OR (welfare w1 support*) OR (economic w1 support*) OR (public w1 assistance*) OR (welfare w1 payment*) OR (public w1 support*) OR (financial w1 support*) OR (welfare w1 service*) OR (direct* w1 payment*) OR (general w1 assistance) OR (Social w1 Support) OR (cash w1 assistance) OR (income w1 assistance) OR (benefit*) OR (social w1 assistance*) OR (social w1 securit*) OR (social w1 welfare) OR (social w1 allowance*) OR (insurance w1 benefit*) OR (social w1 benefit*))	(944,317)
S1	(DE “Social Security”) OR (DE “WELFARE recipients”)	(14,701)

**Web of Science (SSCI + SCI).**

Search string from 2015‐2016 update. Same search string has been used from original search (2011).

**Table:** 

# 14	**5**	#11 AND #8 AND #5 AND #2 *Indexes=SSCI Timespan=2015‐2016*
# 13	**1,092**	#12 AND #9 AND #6 AND #3 *Indexes=SSCI Timespan=2015‐2016*
# 12	**400,859**	#11 OR #10 *Indexes=SCI‐EXPANDED, SSCI Timespan=2015‐2016*
# 11	**47,556**	TI=(Expiration OR Lapse OR Expiry OR Termination OR Duration OR Generosity OR Change OR Entitlement OR Length OR Extend* OR Extension OR Exhaust* OR exit) *Indexes=SCI‐EXPANDED, SSCI Timespan=2015‐2016*
# 10	**400,859**	TS=(Expiration OR Lapse OR Expiry OR Termination OR Duration OR Generosity OR Change OR Entitlement OR Length OR Extend* OR Extension OR Exhaust* OR exit) *Indexes=SCI‐EXPANDED, SSCI Timespan=2015‐2016*
# 9	**688,424**	#8 OR #7 *Indexes=SCI‐EXPANDED, SSCI Timespan=2015‐2016*
# 8	**173,852**	TI=(effect* OR threat* OR incentive* OR disincentive* OR impact* OR motivat*) *Indexes=SCI‐EXPANDED, SSCI Timespan=2015‐2016*
# 7	**688,424**	TS=(effect* OR threat* OR incentive* OR disincentive* OR impact* OR motivat*) *Indexes=SCI‐EXPANDED, SSCI Timespan=2015‐2016*
# 6	**254,682**	#5 OR #4 *Indexes=SCI‐EXPANDED, SSCI Timespan=2015‐2016*
# 5	**19,834**	TI=(Employ* OR Job* OR work* OR (un‐employ* or unemploy*) OR (re‐employ* or reemploy*)) *Indexes=SCI‐EXPANDED, SSCI Timespan=2015‐2016*
# 4	**254,682**	TS=(Employ* OR Job* OR work* OR (un‐employ* or unemploy*) OR (re‐employ* or reemploy*)) *Indexes=SCI‐EXPANDED, SSCI Timespan=2015‐2016*
# 3	**69,098**	#2 OR #1 *Indexes=SCI‐EXPANDED, SSCI Timespan=2015‐2016*
# 2	**6,945**	TI=(“Welfare recipient*” OR “welfare payment*” OR “welfare support*” OR “economic support*” OR “public assistance*” OR “public support*” OR “financial support*” OR “welfare service*” OR “direct* payment*” OR “general assistance” OR “Social Support” OR “cash assistance” OR “income assistance” OR “benefit*” OR “social assistance*” OR “social securit*” OR “social welfare” OR “social allowance*” OR “insurance benefit*” OR “social benefit*” OR “welfare benefit*” OR TANF OR insurance) *Indexes=SCI‐EXPANDED, SSCI Timespan=2015‐2016*
# 1	**69,098**	TS=(“Welfare recipient*” OR “welfare payment*” OR “welfare support*” OR “economic support*” OR “public assistance*” OR “public support*” OR “financial support*” OR “welfare service*” OR “direct* payment*” OR “general assistance” OR “Social Support” OR “cash assistance” OR “income assistance” OR “benefit*” OR “social assistance*” OR “social securit*” OR “social welfare” OR “social allowance*” OR “insurance benefit*” OR “social benefit*” OR “welfare benefit*” OR TANF OR insurance) *Indexes=SCI‐EXPANDED, SSCI Timespan=2015‐2016*

**ProQuest Dissertations & Thesis A&I.**

Search string from 2015‐2016 update. Same search string has been used from original search (2011).

Set#: S1

Searched for: AB,TI(benefit* OR insurance*)

Databases: ProQuest Dissertations & Theses A&I

Results: 130575°

Set#: S2

Searched for: AB,Ti(employ OR job* OR work)

Databases: ProQuest Dissertations & Theses A&I

Results: 622080°

Set#: S3

Searched for: AB,TI(effect* OR incentive* OR impact*)

Databases: ProQuest Dissertations & Theses A&I

Results: 1189593°

Set#: S4

Searched for: AB,TI(expiration* OR termination* OR duration* OR exhaust* OR exit*)

Databases: ProQuest Dissertations & Theses A&I

Results: 76119°

Set#: S5

Searched for: AB,TI(benefit* OR insurance*) AND AB,Ti(employ OR job* OR work) AND AB,TI(effect* OR incentive* OR impact*) AND AB,TI(expiration* OR termination* OR duration* OR exhaust* OR exit*)

Databases: ProQuest Dissertations & Theses A&I

Results: 45°

**International Bibliography of the Social Sciences.**

Search string from 2015‐2016 update. Same search string has been used from original search (2011).

Set#: S1

Searched for: (DE “social security” OR (Welfare adj recipient[*1]) OR (welfare adj payment[*1]) OR (welfare adj support[*1]) OR (economic adj support[*1]) OR (public adj assistance[*1]) OR (public adj support[*1]) OR (financial adj support[*1]) OR (welfare adj service[*1]) OR (direct[*1] adj payment[*1]) OR (general adj assistance) OR (Social adj Support) OR (cash adj assistance) OR (income adj assistance) OR (benefit[*1]) OR (social adj assistance[*1]) OR (social adj securit) OR (social adj welfare) OR (social adj allowance[*1]) OR (insurance adj benefit[*1]) OR (social adj benefit[*1]) OR (welfare adj benefit[*1]) OR TANF OR insurance)

Databases: International Bibliography of the Social Sciences (IBSS)

Results: 65467°

Set#: S2

Searched for: ab(Employ* OR Job* OR work OR un‐employ* OR unemploy* OR re‐employ* OR reemploy*) OR ti(Employ* OR Job* OR work OR un‐employ* OR unemploy* OR re‐employ* OR reemploy*)

Databases: International Bibliography of the Social Sciences (IBSS)

Results: 203853°

Set#: S3

Searched for: ab(effect[*1] OR threat[*1] OR incentive[*1] OR disincentive[*1] OR impact[*1] OR motivat[*1]) OR ti(effect[*1] OR threat[*1] OR incentive[*1] OR disincentive[*1] OR impact[*1] OR motivat[*1])

Databases: International Bibliography of the Social Sciences (IBSS)

Results: 245954°

Set#: S4

Searched for: ab(Expiration[*1] OR Lapse[*1] OR Expiry OR Termination OR Duration[*1] OR Generosity OR Change[*1] OR Entitlement OR Length[*1] OR Extend[*1] OR Extension[*1] OR Exhaust[*1] OR exit[*1]) OR ti(Expiration[*1] OR Lapse[*1] OR Expiry OR Termination OR Duration[*1] OR Generosity OR Change[*1] OR Entitlement OR Length[*1] OR Extend[*1] OR Extension[*1] OR Exhaust[*1] OR exit[*1])

Databases: International Bibliography of the Social Sciences (IBSS)

Results: 185992°

Set#: S5

Searched for: (DE “social security” OR (Welfare adj recipient[*1]) OR (welfare adj payment[*1]) OR (welfare adj support[*1]) OR (economic adj support[*1]) OR (public adj assistance[*1]) OR (public adj support[*1]) OR (financial adj support[*1]) OR (welfare adj service[*1]) OR (direct[*1] adj payment[*1]) OR (general adj assistance) OR (Social adj Support) OR (cash adj assistance) OR (income adj assistance) OR (benefit[*1]) OR (social adj assistance[*1]) OR (social adj securit) OR (social adj welfare) OR (social adj allowance[*1]) OR (insurance adj benefit[*1]) OR (social adj benefit[*1]) OR (welfare adj benefit[*1]) OR TANF OR insurance) AND (ab(Employ* OR Job* OR work OR un‐employ* OR unemploy* OR re‐employ* OR reemploy*) OR ti(Employ* OR Job* OR work OR un‐employ* OR unemploy* OR re‐employ* OR reemploy*)) AND (ab(effect[*1] OR threat[*1] OR incentive[*1] OR disincentive[*1] OR impact[*1] OR motivat[*1]) OR ti(effect[*1] OR threat[*1] OR incentive[*1] OR disincentive[*1] OR impact[*1] OR motivat[*1])) AND (ab(Expiration[*1] OR Lapse[*1] OR Expiry OR Termination OR Duration[*1] OR Generosity OR Change[*1] OR Entitlement OR Length[*1] OR Extend[*1] OR Extension[*1] OR Exhaust[*1] OR exit[*1]) OR ti(Expiration[*1] OR Lapse[*1] OR Expiry OR Termination OR Duration[*1] OR Generosity OR Change[*1] OR Entitlement OR Length[*1] OR Extend[*1] OR Extension[*1] OR Exhaust[*1] OR exit[*1]))

Databases: International Bibliography of the Social Sciences (IBSS)

These databases are searched for part of your query.

Results: 1199°

Set#: S6

Searched for: (DE “social security” OR (Welfare adj recipient[*1]) OR (welfare adj payment[*1]) OR (welfare adj support[*1]) OR (economic adj support[*1]) OR (public adj assistance[*1]) OR (public adj support[*1]) OR (financial adj support[*1]) OR (welfare adj service[*1]) OR (direct[*1] adj payment[*1]) OR (general adj assistance) OR (Social adj Support) OR (cash adj assistance) OR (income adj assistance) OR (benefit[*1]) OR (social adj assistance[*1]) OR (social adj securit) OR (social adj welfare) OR (social adj allowance[*1]) OR (insurance adj benefit[*1]) OR (social adj benefit[*1]) OR (welfare adj benefit[*1]) OR TANF OR insurance) AND (ab(Employ* OR Job* OR work OR un‐employ* OR unemploy* OR re‐employ* OR reemploy*) OR ti(Employ* OR Job* OR work OR un‐employ* OR unemploy* OR re‐employ* OR reemploy*)) AND (ab(effect[*1] OR threat[*1] OR incentive[*1] OR disincentive[*1] OR impact[*1] OR motivat[*1]) OR ti(effect[*1] OR threat[*1] OR incentive[*1] OR disincentive[*1] OR impact[*1] OR motivat[*1])) AND (ab(Expiration[*1] OR Lapse[*1] OR Expiry OR Termination OR Duration[*1] OR Generosity OR Change[*1] OR Entitlement OR Length[*1] OR Extend[*1] OR Extension[*1] OR Exhaust[*1] OR exit[*1]) OR ti(Expiration[*1] OR Lapse[*1] OR Expiry OR Termination OR Duration[*1] OR Generosity OR Change[*1] OR Entitlement OR Length[*1] OR Extend[*1] OR Extension[*1] OR Exhaust[*1] OR exit[*1])) AND pd(20150101‐20160301)

Databases: International Bibliography of the Social Sciences (IBSS)

These databases are searched for part of your query.

Results: 0°

**Econlit.**

Search string from 2015‐2016 update. Same search string has been used from original search (2011).

**Table:** 

**#**	**Query**	**Limiters/Expanders**	**Results**
**S7**	S3 AND S4 AND S5 AND S6	Limiters‐ Published Date: 20150601‐20160231 Search modes‐ Boolean/Phrase	88
**S6**	S1 OR S2	Limiters‐ Published Date: 20150601‐20160231 Search modes‐ Boolean/Phrase	185,032
**S5**	AB (((Expir*) or (Lapse) or (Terminat*) or (Duration*) or (Generosity) or (Change) or (Entitlement*) or (Length*) or (Extend*) or (Exhaust*) OR Exit*)) OR TI (((Expir*) or (Lapse) or (Terminat*) or (Duration*) or (Generosity) or (Change) or (Entitlement*) or (Length*) or (Extend*) or (Exhaust*) OR Exit*))	Limiters‐ Published Date: 20150601‐20160231 Search modes‐ Boolean/Phrase	171,799
**S4**	AB ((Effect* OR Threat* OR Incentive* OR Disincentive* OR Impact* OR Motivat*)) OR TI ((Effect* OR Threat* OR Incentive* OR Disincentive* OR Impact* OR Motivat*))	Limiters‐ Published Date: 20150601‐20160231 Search modes‐ Boolean/Phrase	344,887
**S3**	AB ((Employ* OR Job* OR Work* OR (Un‐employ* OR Unemploy*) OR (Re‐employ* OR Reemploy*))) OR TI ((Employ* OR Job* OR Work* OR (Un‐employ* OR Unemploy*) OR (Re‐employ* OR Reemploy*)))	Limiters‐ Published Date: 20150601‐20160231 Search modes‐ Boolean/Phrase	196,144
**S2**	AB ((social N1 securit*) OR (social N1 support) OR (social N1 welfare) OR (Welfare N1 recipient*) OR (Welfare N1 service*) OR (support) OR (assistance) OR (aid) OR (Relief) OR (Benefit*) OR (Allowance*) OR (Payment*) OR (Securit*) OR (TANF) OR insurance*)) OR TI ((social N1 securit*) OR (social N1 support) OR (social N1 welfare) OR (Welfare N1 recipient*) OR (Welfare N1 service*) OR (support) OR (assistance) OR (aid) OR (Relief) OR (Benefit*) OR (Allowance*) OR (Payment*) OR (Securit*) OR (TANF) OR insurance*))	Limiters‐ Published Date: 20150601‐20160231 Search modes‐ Boolean/Phrase	181,032
**S1**	SU social security	Limiters‐ Published Date: 20150601‐20160231 Search modes‐ Boolean/Phrase	10,296

**Cochrane Library.**

Search string from 2015‐2016 update. Same search string has been used from original search (2011).

**Table:** 

**#**	**Query**	**Hits**
**5**	#1 AND #2 AND #3 AND #4:ti,ab,kw Online Publication Date from Jun 2015 to Feb 2016 (Word variations have been searched)	49
**4**	((Expir) or (Lapse) or (Terminat) or (Duration) or (Generosity) or (Change or changes) or (Entitlement) or (Length) or (Extend) or (Extension) or (Exhaust) or (exit)):ti,ab,kw Online Publication Date from Jun 2015 to Feb 2016 (Word variations have been searched)	406
**3**	((effect) or (threat) or (incentive) or (disincentive) or (impact) or (motivat)):ti,ab,kw Online Publication Date from Jun 2015 to Feb 2016 (Word variations have been searched)	827
**2**	((Employ) or (Job) or (work) or (unemploy) or (reemploy)): ti,ab,kw Online Publication Date from Jun 2015 to Feb 2016 (Word variations have been searched)	162
**1**	((“Social Security”) or (Welfare next recipient) or (welfare next payment) or (welfare next recipient) or (welfare next support) or (economic next support) or (public next assistance) or (welfare next payment) or (public next support) or (financial next support) or (welfare next service) or (direct next payment) or (general next assistance) or (Social next Support) or (cash next assistance) or (income next assistance) or (benefit) or (social next assistance) or (social next securit) or (social next welfare) or (social next allowance) or (insurance next benefit) or (social next benefit) or (welfare next benefit) or (TANF) or insurance): ti,ab,kw Online Publication Date from Jun 2015 to Feb 2016 (Word variations have been searched)	410

**Social Care Online.**

Records from 2015‐2016 (11 records) was manually exported.

**Table:** 

**Search**	**Query**	**Hits**
1	freetext="social security” or topic="benefits” or freetext="benefit*” or freetext="support” or freetext="insurance”	**43551**
2	(topic="unemployment” or freetext="unemployment” or freetext="work” or topic="employment” or freetext="employment” or freetext="job*”)	**69650**
3	(freetext="effect” or freetext="impact*” or freetext="incentive*” or freetext=motivation)	**36043**
4	(freetext="duration” or freetext="exit*” or freetext="exhaust*” or freetext=entitl*)	**1133**
5	((freetext="social security” or topic="benefits” or freetext="benefit*” or freetext="support” or freetext="insurance”) AND (topic="unemployment” or freetext="unemployment” or freetext="work” or topic="employment” or freetext="employment” or freetext="job*”) AND (freetext="effect” or freetext="impact*” or freetext="incentive*” or freetext=motivation) AND (freetext="duration” or freetext="exit*” or freetext="exhaust*” or freetext=entitl*))	**109 (11)**

**Forskningsdatabasen (The Danish National Research database).**

**Table:** 

**Search**	**Query**	**Hits**
**1**	kw:support* OR benefit* OR welfare OR insurance*	2034
**2**	kw:emplo* OR job* OR work	3781
**3**	kw:effect* OR incentive* OR impact* OR motivat*	2818
**4**	kw:expiration* OR termination* OR duration* OR change* OR exhaust* OR exit*	222
**5**	((kw:support* OR benefit* OR welfare OR insurance*) AND (kw:emplo* OR job* OR work) AND (kw:effect* OR incentive* OR impact* OR motivat*) AND (kw:expiration* OR termination* OR duration* OR change* OR exhaust* OR exit*))	2

**Search for grey literature**

The complexity of the search string was reduced in order to search for grey literature on the internet resources described in the search chapter. In general, a two‐term search strategy was used: “Benefit* AND unemployment”. When possible, this two‐term strategy was enhanced to “Benefit* AND unemployment AND effect* AND duration*”. The enhanced search string was used to search Google Scholar, where “Benefit unemployment effect duration” was searched in the field “all of the words”.

### 12.2 FLOW CHART FOR LITERATURE SEARCH

### 12.3 FIRST AND SECOND LEVEL SCREENING

First level screening is on the basis of titles and abstracts. Second level is on the basis of full text

Reference id. No. :

Study id. No.:

Reviewer's initials:

Source:

Year of publication:

Duration of study:

Country/countries of origin

Author

The study will be excluded if one or more of the answers to question 1‐3 are ‘No’. If the answers to question 1 to 3 are ‘Yes’ or ‘Uncertain’, then the full text of the study will be retrieved for second level eligibility. All unanswered questions need to be posed again on the basis of the full text. If not enough information is available, or if the study is unclear, the author of the study will be contacted if possible.

**First level screening questions are based on titles and abstracts**

1. Are the participants' unemployed individuals receiving some kind of benefit during their unemployment?
  Yes ‐ include
  No – if no then stop here and exclude
  Uncertain ‐ include

Question 1 guidance:

This includes all types of unemployment benefits both unemployment insurance benefits, unemployment assistance benefits and social assistance benefits.

2. Does the study focus on time limits in the unemployment benefit eligibility period or exhaustion of unemployment benefits or entitlement to unemployment benefits or maximum duration of unemployment benefits etc.?
  Yes ‐ include
  No – if no then stop here and exclude
  Uncertain ‐ include

Question 2 guidance:

The intervention is a change in the maximum duration of any kind of unemployment benefit with a known expiration date. This intervention can be referred to in different ways.

3. Is this study a primary quantitative study?
  Yes ‐ include
  No – if no then stop here and exclude
  Uncertain ‐ include

Question 3 guidance:

We are only interested in primary quantitative studies, where the authors have analyzed the data. We are not interested in theoretical papers on the topic or surveys/reviews of studies of the topic. (This question may be difficult to answer on the base of titles and abstracts alone.)

**Second level screening questions based on full text**

4. Does the study estimate an effect, using a control group or using an estimated counterfactual?
  Yes ‐ include
  No – if no then stop here and exclude
  Uncertain ‐ include

Question 4 guidance

E.g. 1) Randomised controlled trials including cluster randomisation and quasi randomised controlled study designs (i.e. participants are allocated by means such as alternate allocation, person's birth date, the date of the week or month, case number or alphabetical order), 2) non randomised controlled study designs (i.e. quasi‐experimental designs) such as controlled two group study designs or 3) study designs based on observational data, where the effect is estimated by statistical methods.

5. Does the study examine exits to employment?
  Yes – include
  No – if no then stop here and exclude
  Uncertain – include

Question 5 guidance:

The primary outcome is exits to employment. Studies only looking at exits to other destinations (such as other kinds of benefits or out of the labour force) or studies who do not distinguish between destinations will not be included.

### 12.4 CODING FORM

**Table:** 

**Language**
**Journal**
**Year**
**Country**
**Time period covered by data**
**Type of unemployment scheme (UI, social benefit other (specify))**
**Participation characteristics (age, gender, education, ethnicity, eligibility requirements for benefits)**
**Benefit level/replacement rate**
**Labour market conditions (unemployment rate and/or vacancy rate)**
**Benefit level/replacement rate available after exhaustion if any**
**Is compulsory activation part of the system? If yes, describe the elements of the programme (education, work, training, self‐employment, job search assistance)**
**Maximum duration of unemployment benefits**
**Type of data used (register, questionnaire, other (specify))**
**Sampling frequency**
**Time interval the outcome measure is based on (if different from sampling frequency)**
**Is there correction for unobserved heterogeneity? If yes, how?**
**Sample size (Treatment/control)**
**Is there correction for censoring (yes/no)**

### 12.5 ASSESSMENT OF RISK OF BIAS IN INCLUDED STUDIES

**Risk of bias table**

**Table:** 

**Item**	**Judgement** ^a^	**Description** (quote from paper, or describe key information)
1. Sequence generation		
2. Allocation concealment		
3. Confounding^b^, ^c^		
4. Blinding?^b^		
5. Incomplete outcome data addressed?^b^		
6. Free of selective reporting?^b^		
7. Free of other bias?		
*8. A priori* protocol?^d^		
*9. A priori* analysis plan?^e^		

a Some items on low/high risk/unclear scale (double‐line border), some on 5 point scale/unclear (single line border), some on yes/no/unclear scale (dashed border). For all items, record “unclear” if inadequate reporting prevents a judgement being made.

b For each outcome in the study.

c This item is only used for NRCTs and NRSs. It is based on list of confounders considered important at the outset and defined in the protocol for the review (*assessment against worksheet*).

d Did the researchers write a protocol defining the study population, intervention and comparator, primary and other outcomes, data collection methods, etc. in advance of starting the study?

e Did the researchers have an analysis plan defining the primary and other outcomes, statistical methods, subgroup analyses, etc. in advance of starting the study?

**Risk of bias tool**

**Studies for which RoB tool is intended**

The risk of bias model was developed by Prof. Barnaby Reeves in association with the Cochrane Non‐Randomised Studies Methods Group.^20^ This model, an extension of the Cochrane Collaboration's risk of bias tool, covers risk of bias in both randomised controlled trials (RCTs and QRCTs) and in non‐randomised studies (NRCTs and NRSs).

The point of departure for the risk of bias model is the Cochrane Handbook for Systematic Reviews of interventions (Higgins & Green, 2008). The existing Cochrane risk of bias tool needs elaboration when assessing non‐randomised studies because, for non‐randomised studies, particular attention should be paid to selection bias / risk of confounding. Additional item on confounding is used only for non‐randomised studies (NRCTs and NRSs) and is not used for randomised controlled trials (RCTs and QRCTs).

**Assessment of risk of bias**

Issues when using modified RoB tool to assess included non‐randomised studies:

- Use existing principle: score judgment and provide information (preferably direct quote) to support judgment
- Additional item on confounding used only for non‐randomised studies (NRCTs and NRSs).
- 5‐point scale for some items (distinguish “unclear” from intermediate risk of bias).
- Keep in mind the general philosophy – assessment is not about whether researchers could have done better but about risk of bias; the assessment tool must be used in a standard way whatever the difficulty / circumstances of investigating the research question of interest and whatever the study design used.
- Anchors: “1/No/low risk” of bias should correspond to a high quality RCT. “5/high risk” of bias should correspond to a risk of bias that means the findings should not be considered (too risky, too much bias, more likely to mislead than inform)

1. Sequence generation
  - Low/high/unclear RoB item
  - Always high RoB (not random) for a non‐randomised study
  - Might argue that this item redundant for NRS since always high – but important to include in RoB table (‘level playing field’ argument)
2. Allocation concealment
  - Low/high/unclear RoB item
  - Potentially low RoB for a non‐randomised study, e.g. quasi‐randomised (so high RoB to sequence generation) but concealed (reviewer judges that the people making decisions about including participants didn't know how allocation was being done, e.g. odd/even date of birth/hospital number)
3. RoB from confounding (additional item for NRCT and NRS; assess for each outcome)
  - Assumes a pre‐specified list of potential confounders defined in the protocol
  - Low(1) / 2 / 3 / 4 / high(5) / unclear RoB item
  - Judgment needs to factor in:
    ○ proportion of confounders (from pre‐specified list) that were considered
    ○ whether most important confounders (from pre‐specified list) were considered
    ○ resolution/precision with which confounders were measured
    ○ extent of imbalance between groups at baseline
    ○ care with which adjustment was done (typically a judgment about the statistical modeling carried out by authors)
  - Low RoB requires that all important confounders are balanced at baseline (not primarily/not only a statistical judgment OR measured ‘well’ and ‘carefully’ controlled for in the analysis.
  Assess against pre‐specified worksheet. Reviewers will make a RoB judgment about each factor first and then ‘eyeball’ these for the judgment RoB table.
4. RoB from lack of blinding (assess for each outcome, as per existing RoB tool)
  - Low(1) / 2 / 3 / 4 / high(5) / unclear RoB item
  - Judgment needs to factor in:
    ○ nature of outcome (subjective / objective; source of information)
    ○ who was / was not blinded and the risk that those who were not blinded could introduce performance or detection bias
    ○ see Ch.8
5. RoB from incomplete outcome data (assess for each outcome, as per existing RoB tool)
  - Low(1) / 2 / 3 / 4 / high(5) / unclear RoB item
  - Judgment needs to factor in:
    ○ reasons for missing data
    ○ whether amount of missing data balanced across groups, with similar reasons
    ○ whether censoring is less than or equal to 25% and taken into account
    ○ see Ch.8
6. RoB from selective reporting (assess for each outcome, NB different to existing Ch.8 recommendation)
  - Low(1) / 2 / 3 / 4 / high(5) /unclear RoB item
  - Judgment needs to factor in:
    ○ existing RoB guidance on selective outcome reporting (see Ch.8)
    ○ also, extent to which analyses (and potentially other choices) could have been manipulated to bias the findings reported, e.g. choice of method of model fitting, potential confounders considered / included
    ○ look for evidence that there was a protocol in advance of doing any analysis / obtaining the data (difficult unless explicitly reported); NRS very different from RCTs. RCTs must have a protocol in advance of starting to recruit (for REC/IRB/other regulatory approval); NRS need not (especially older studies)
    ○ Hence, separate yes/no items asking reviewers whether they think the researchers had a pre‐specified protocol and analysis plan.
7. RoB from other bias (assess for each outcome, NB different to existing Ch.8 recommendation)
  - Low(1) / 2 / 3 / 4 / high(5) /unclear RoB item
  - Judgment needs to factor in:
    ○ existing RoB guidance on other potential threats to validity (see Ch.8)
    ○ also, assess whether suitable cluster analysis is used (e.g. cluster summary statistics, robust standard errors, the use of the design effect to adjust standard errors, multilevel models and mixture models), if assignment of units to treatment is clustered

**Confounding worksheet**

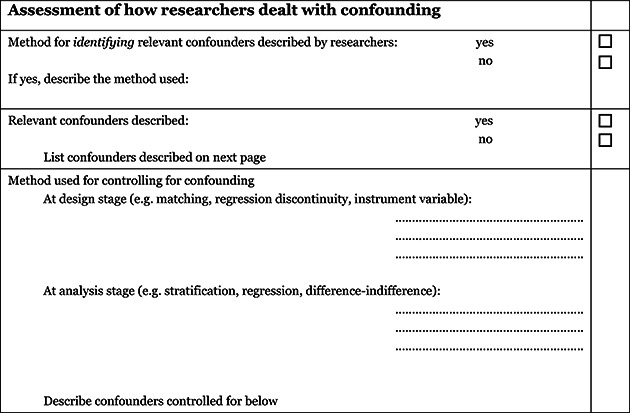

**Confounders described by researchers**

Tick (yes[0]/no[1] judgment) if confounder considered by the researchers [Cons'd?]

Score (1[good precision] to 5[poor precision]) precision with which confounder measured

Score (1[balanced] to 5[major imbalance]) imbalance between groups

Score (1[very careful] to 5[not at all careful]) care with which adjustment for confounder was carried out

**Table:** 

**Confounder**	Considered	Precision	Imbalance	Adjustment
Gender	□	□	□	□
Age	□	□	□	□
Education	□	□	□	□
Ethnicity	□	□	□	□
Labour market conditions	□	□	□	□
Unemployment duration	□	□	□	□
Censoring	□	□	□	□
Unobservables^21^	□	Irrelevant	□	□
Other:	□	□	□	□

**User guide for unobservables**

Selection bias is understood as systematic baseline differences between groups and can therefore compromise comparability between groups. Baseline differences can be observable (e.g. age and gender) and unobservable (to the researcher; e.g. motivation and ‘ability’). There is no single non‐randomised study design that always solves the selection problem. Different designs solve the selection problem under different assumptions and require different types of data. Especially how different designs deal with selection on unobservables varies. The “right” method depends on the model generating participation, i.e. assumptions about the nature of the process by which participants are selected into a programme.

As there is no universal correct way to construct counterfactuals we will assess the extent to which the identifying assumptions (the assumption that makes it possible to identify the counterfactual) are explained and discussed (preferably the authors should make an effort to justify their choice of method). We will look for evidence that authors using e.g. (this is NOT an exhaustive list):

**Natural experiments:**

Discuss whether they face a truly random allocation of participants and that there is no change of behavior in anticipation of e.g. policy rules.

**Instrument variable (IV):**

Explain and discuss the assumption that the instrument variable does not affect outcomes other than through their effect on participation.

**Matching (including propensity scores):**

Explain and discuss the assumption that there is no selection on unobservables, only selection on observables.

**(Multivariate, multiple) Regression:**

Explain and discuss the assumption that there is no selection on unobservables, only selection on observables. Further discuss the extent to which they compare comparable people.

**Regression Discontinuity (RD):**

Explain and discuss the assumption that there is a (strict!) RD treatment rule. It must not be changeable by the agent in an effort to obtain or avoid treatment. Continuity in the expected impact at the discontinuity is required.

**Difference‐in‐difference (Treatment‐control‐before‐after):**

Explain and discuss the assumption that outcomes of participants and nonparticipants evolve over time in the same way.

## 13 Analysis

### 13.1 SENSITIVITY ANALYSIS

**Figure 133.1:**
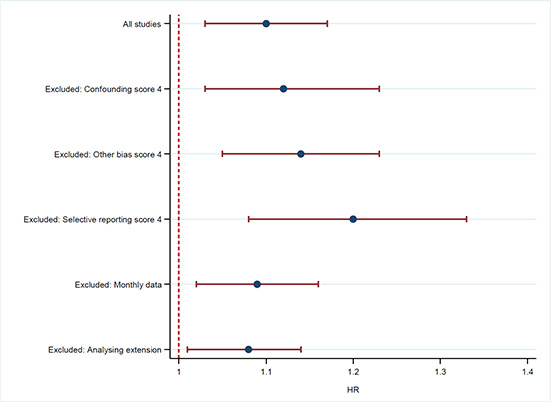
Forest plot, hazard ratio

## 14 Data appendices

### 14.1 DATA EXTRACTION

#### 14.1.1 Descriptive data

**Table:** 

**Author**	**Addison & Portugal**	**Ahn & Ugidos‐Olazabal**	**Amarante, Arim & Dean**
Title	How do different entitlements to unemployment benefits affect the transitions from unemployment into employment?	Duration of unemployment in Spain: Relative effects of unemployment benefit and family characteristics	Unemployment Insurance Design and Its Effects: Evidence for Uruguay
Year	2008	1995	2013
Country	Portugal	Spain	Uruguay
Journal	Economic Letters	Oxford Bulletin of Economics and Statistics	Desarrollo y Sociedad
Time period covered by data	1992‐1997 (p. 207)	1985	15 months before and 15 months after modification of programme (February 2009) (p.17). (From November 2007 to April 2010)
Type of unemployment scheme	Unemployment insurance	Unemployment insurance	Unemployment insurance
Type of data used	Portuguese Quarterly Employment Survey (p.207)	Survey Data (p.250)	Administrative records from Unemployment insurance program and sample of longitudinal data on social security records (p. 17). Household surveys (p. 15)
Target group	Eligible unemployed individuals, whose benefits differ by three (two for those aged 24 to 25) months (Regression Discontinuity design). Target group comprises pair of individuals aged: 24 to 25 years, 29 to 30 years, 34 to 35 years, 39 to 40 years, 44 to 45 years, 49 to 50 years and 54 to 55 years. (p.207). Males only.	Individuals aged 16‐64 at the time of survey. Last or current unemployment spell examined (p.251)	Unemployed in insurance scheme. Effects of extensions for older workers (i.e. 50 years or older): treatment group aged 50‐53, control group aged 46‐49 (p. 25)
Target group ‐ eligibility, requirements for benefits	Not specified.	Must be enrolled in social security program (p.256)	3 possible reasons for entering program: job loss, job suspension, job reduction. Old regime: workers should have worked at least 6 months in the previous year and be involuntarily unemployed. Workers cannot re‐enter program until a year has passed since last benefit (p. 11)
Benefit level/replacement rate	Replacement rate is 65% of previous earnings (p.207)	Not mentioned	Old Regime: subsidy 50% of the average wage of the last 6 months or monthly subsidy equivalent to 12 working days. Amount could never be less than half the minimum wage (p. 11‐13). New Regime: decreasing scheme for benefits (p. 12‐13)
Benefit level/replacement rate available after exhaustion	Not reported	Not mentioned.	Not reported
Is compulsory activation part of the system?	Not reported	Not reported	Not reported
Maximum duration of unemployment benefits	Maximum duration is dependent on age: Under 25 years: 10 months 25‐29 years: 12 months 29‐54 years: Increases with three months intervals every five years 55 years and over: 30 months (p.207)	Not mentioned	Old Regime: 6 months (equivalent to 72 days of labour). New regime: Job loss/reduction: 6 months (72 days of labour), job suspension: 4 months (p. 11‐13). Workers over 50 in new regime: extension up to a year (instead of just 6 months)
Destination	Employment (p.209)	Any exit Employment Non‐participation	Not reported
Sampling frequency	Monthly (p.207)	Not reported	Monthly
Time interval the outcome measure is based on	Not reported	Discrete time periods are constructed (p.250)	Monthly (p. 22)
Sample size	12.913 (table 2, p. 208)	Men: 2877 Women: 1262 Total: 4139 (p.251)	N of treated obs. before: 16,355; N of treated obs. After: 23,568; N of control obs. Before: 8,862; N of control obs. After: 8,126 (p. 24)
Are the labour market conditions described?	Conditions are briefly compared to US (p.207)	Not reported	Development of Unemployment insurance beneficiaries from 1992‐2009 described on p. 16
Censoring level	Transitions from unemployment to employment are traced for up to five quarters (p.207). Levels not mentioned.	Spells censored at the end of the 81st month (p.250)	Not reported
Is there correction for censoring? (Yes/No, ref)	Yes	Yes (p.253)	Not reported

**Table:** 

**Author**	**Arntz, Simon & Wilke**	**Arranz, Bulló & Muro**	**Barbanchon**
Title	Bounds Analysis of Competing Risks: A Non‐parametric Evaluation of the Effect of Unemployment Benefits on Migration	DO UNEMPLOYMENT BENEFIT LEGISLATIVE CHANGES AFFECT JOB FINDING? EVIDENCE FROM THE SPANISH 1992 UI REFORM ACT	The Effect of the Potential Duration of Unemployment Benefits on Unemployment Exits to Work and Match Quality in France
Year	2014	2008	2016
Country	Germany	Spain	France
Journal	Empirical Economics	Instituto de Estudios Fiscales	Labour Economics
Time period covered by data	Unemployment spells starting in 1995‐1996 (pre‐reform) and 1999‐2000 (post‐reform) (p. 205)	1987‐1997	2000‐2002 (P. 6)
Type of unemployment scheme	Unemployment insurance (UB)	Unemployment insurance	Unemployment insurance (UB)
Type of data used	Administrative data (p. 200). Sample drawn from Employee and Benefit Recipient History (V6.0) of the Institute of Employment Research (IAB) ‐ which comprises 50 % of the male working population. (p. 205)	Administrative registers	Matched dataset of French employment/unemployment registers (p. 6 and Appendix A)
Target group	Unemployed men, with previous full‐time job and job located in western Germany. Treatment group: 42‐44 years / Control group: 36‐41 years. (p. 204‐05)	18‐59 years‐old who started receiving Unemployment insurance in 1991 and in 1993 (p. 15) + sample characteristics in table 3 (p. 16)	New UB claimants (without any residual benefits from former unemployment spell) with short and extended potential benefit duration (PBD). Unemployed over 50 at registration are excluded. Jobseekers subject to very specific Unemployment insurance rules (recurrent temporary workers, artists, technicians in culture sector) excluded (p. 6‐7)
Target group ‐ eligibility, requirements for benefits	Potential UB benefit and duration are determined by age of claimant and creditable working months (p.202)	Eligible for Unemployment insurance are workers whose unemployment situation is recognized according to law by the labour authority; i.e., the job was lost involuntarily, including end of a fixed‐term contract. Before the reform: Social Security contributions for a minimum of six months during the four years preceding unemployment. After the reform: contributions for a minimum of twelve months during the six years preceding unemployment. (p. 10)	Depends on past employment duration over a reference period ‐ the reference period in this study is one year before job separation. (p. 4). For the group with short PBD the past employment duration is between 6 and 8 months ‐ they are entitled to 7 months of UB. For the group with extended PBD the past employment duration exceeds 8 months ‐ they are entitled to 15 months of UB (p. 4‐5)
Benefit level/replacement rate	Fixed ratio of former wage income: 68% with dependent children / 63% without dependent children (p.202)	Before the reform: 80% the first 6 months, 70% from 7th to 12th month, 60% from 13th onwards. After the reform: 70% the first 6 months, 60% the rest of the period (p. 12)	Replacement rate decreases with the “reference wage” (the average wage over the year preceding job loss). Reference wage at minimum wage receives 66% replacement. Reference wage twice as large as minimum wage receives 57.4%. UB level capped at 5400 Euros per month. Replacement rate constant during whole PBD since 2001 (rate was decreasing before 2001). (p. 5)
Benefit level/replacement rate available after exhaustion	Unemployment Assistance ‐ fixed ratio of former income (57% of former income with dependent children / 53 % of former income without depended children) (p.202)	75% of statutory minimum wage (p.47)	Means‐tested social assistance, Revenu Minium d'Insertion. Amount received depends on family composition and earnings (p. 6)
Is compulsory activation part of the system?	Not reported	Not reported	ALMP is available, and have become more frequent since 2001 (p. 6)
Maximum duration of unemployment benefits	Before 1997‐reform: Depending on age and work history an individual can get up till 22 months of UB After 1997‐reform: Potential benefit duration (PBD) shortened for several groups, see Table 1 (p.203). After 1997 the reform shortened the PBD for those aged 42 and above, while the wage replacement ratio of UB remained unchanged. The reduction in PBD depends on the age when initially claiming unemployment and the employment history. Individuals below age 42 receive up to 12 months of PBD depending on their CWM within the extended claim period prior to claiming UB. An individual aged 42 with a length of 28 or more working months within the claim period, however, received an extended PBD of up to 18 months before the reform, but was eligible for a maximum of only 12 months after the reform. The shortening of PBD was even stronger for those aged 44 since the maximum PBD before the reform was 22 months compared to l 2 months after the reform (p. 203)	Before the reform: 6‐12 months contribution=3 months, 13‐18 months contribution=6 months ‐> 48+ contributions=24 months. After the reform: 12‐17 months contribution=4 months, 18‐23 months contribution=6 months ‐> 72+ contributions=24 months. (p. 10 + table 1, p. 11)	Past employment duration between 6‐8 months: entitled to 7 months UB; past employment duration exceeding 8 months: 15 months UB (p. 4‐5)
Destination	Distant and local employment (p.217)	Employment	Employment
Sampling frequency	Daily (p. 200)	Monthly	Monthly
Time interval the outcome measure is based on	Daily	Monthly	Monthly
Sample size	Treatment pre‐1997: 39,434 Treatment post‐1997: 36,104 Control pre‐1997: 104,069 Control post‐1997: 94,309 (p. 207)	1991: 42,029 individuals, 1993: 35,845 individuals (p. 18)	All: 16,692; Bandwidth around threshold: 4 months (8352), 2 months (3837), 1 month (1817) (p. 29‐30)
Are the labour market conditions described?	Not reported	Unemployment rate above 15% after the 1992 crisis (p. 7)	Not reported
Censoring level	Treatment pre‐1997: 13,3 % Treatment post‐1997: 15,7% Control pre‐1997: 10,7% Control post‐1997: 13,7% (p.211)	77.5% in the 1991‐sample, 73% in the 1993‐sample (p. 18)	Spells are censored in December 2004 (p.32). 14% of new job spells are censored (p.9)
Is there correction for censoring? (Yes/No, ref)	Yes (p.211‐12)	Yes (p. 29)	Yes (p.18). It is stated in a footnote that “We can ignore censoring issues: there are virtually no employment spells censored before 8 months”
**Author**	**Belzil**	**Bover, Arellano & Bentolila**	**Caliendo, Tatsiramos & Uhlendorff**
Title	Unemployment Insurance and Unemployment Over time: An analysis with Event History Data	Unemployment Duration, Benefit Duration and the Business Cycle	Benefit duration, unemployment duration and job match quality: a regression‐discontinuity approach
Year	1995	2002	2013
Country	Canada	Spain	Germany
Journal	The Review of Economics and Statistics	The Economic Journal	*Journal of applied econometrics*
Time period covered by data	1972‐1984	1987‐1994	2001‐2003 (p.610)
Type of unemployment scheme	Unemployment insurance	Unemployment insurance	Unemployment insurance
Type of data used	Administrative data (p.118)	Survey data ‐ Spanish Labour Force Survey (p.229)	Register data ‐ Random inflow sample (p. 610)
Target group	Unemployment insurance claimants. (Unemployment insurance claims that overlap the research design time periods (before/after September 1977) are excluded in order to create a control and comparison group) (p.118).	Men aged 20‐64 (p.230,259)	Sample restricted to individuals from West Germany, who have been employed for at least 36 months the last seven years and have been working in regular employment for 12 months in the last year prior to unemployment. Further restrictions: Men aged 44‐46 years Women aged 43,5‐46,5 years (p.610)
Target group ‐ eligibility, requirements for benefits	Eligibility criteria does not require involuntary unemployment, as in many other Unemployment insurance systems (p.118)	Contributions to program, while employed, must be made. Dismissal from job, which have been held for at least a year (p.228)	Individuals must have been employed at least 12 months within previous three years before unemployment (p.608)
Benefit level/replacement rate	67% of insurable earnings (p.118)	First 6 months: 70% replacement rate after the first six months: 60 % replacement rate. Subject to a floor of minimum 75% of minimum wage, and ceiling related to number of dependants. (p.228)	Depends on family status and previous earnings. People with at least one child are entitled to 67%, others are entitled to 60% of former average net daily income (p.609)
Benefit level/replacement rate available after exhaustion	Not reported	Unemployment assistance System: 75% of minimum wage up to 2 years. (p.228)	UA: Means‐tested unemployment assistance, 57%/53% (with or without children) earnings‐related replacement rate. SA: Means‐tested, flat‐rate (p.609)
Is compulsory activation part of the system?	Not reported	Not reported	Not reported
Maximum duration of unemployment benefits	Before September 1997: Based on difference between regional and national unemployment rate and on weeks worked. After September 1977: Based on previous weeks worked and the absolute level of the regional aggregate unemployment rate. (p.118)	Duration is equal to one‐third of the accumulated job tenure, with a maximum of 2 years (p.228)	Restricts to those who have worked at least 36 months during the past seven years (so all entitled to max benefit duration given age) and 44‐46 years of age. Below 45 years of age are entitled to 12 months and above 45 years of age are entitled to 18 months
Destination	Employment (p.118)	Employment (p. 231)	Employment (p.610)
Sampling frequency	Daily (p.118)	Survey is conducted every quarter (p.229,260)	Daily (p. 610)
Time interval the outcome measure is based on	Weekly (p.118)	Monthly (p.225)	Monthly (p.610)
Sample size	4850 (p.118)	27.006 spells of unemployment (p.259‐261)	Males: 2241; Females: 2776. (p.610)
Are the labour market conditions described?	Not reported	Yes (p.224,228‐29)	No
Censoring level	Not reported	54,15 % (p.261)	25‐27% right censored (p.610)
Is there correction for censoring? (Yes/No, ref)	Not reported	Yes (p.233)	Not reported

**Table:** 

**Author**	**Card, Chetty & Weber**	**Chang**
Title	CASH‐ON‐HAND AND COMPETING MODELS OF INTERTEMPORAL BEHAVIOR: NEW EVIDENCE FROM THE LABOR MARKET	Essays on network effects, unobserved heterogeneity, and job search
Year	2007	2010
Country	Austria	USA
Journal	The Quarterly Journal of Economics	Dissertation, The Johns Hopkins University
Time period covered by data	1981‐2001	2000‐2001 (p.107‐8)
Type of unemployment scheme	Unemployment insurance	Unemployment insurance
Type of data used	Administrative data from the Austrian Social Security Registry (p.1522)	Individual level survey data from The Study of Unemployment Insurance Exhaustees (p.106)
Target group	Individuals ‐ who have lost their job involuntary ‐ aged 20‐50 at time of job separation, and who have worked between 1‐5 years in previous job. Construction workers and individuals who experience recall are excluded from sample (p.1523)	Sample restricted to individuals, who are entitled to at least 26 weeks of Unemployment insurance. Individuals over 55 years or with a definite job recall are excluded. (p.109)
Target group ‐ eligibility, requirements for benefits	Individuals must have worked for 12 months in the two years prior to spell (p.1522)	To qualify for Unemployment insurance applications, the claimants must possess legal working status and minimum earnings requirements (the minimum earnings requirements for eligibility ranges from $130 to $3400 between different states, with a typical state requirement for previous earnings over $1500)
Benefit level/replacement rate	Replacement rate approximately 55% of previous net wage (p. 1552)	In sample: Average $184,4 per week (min:$31,2/max:$328) (p. 110)
Benefit level/replacement rate available after exhaustion	Unemployment assistance (UA): average replacement rate is 38% of Unemployment insurance benefit level	Not reported
Is compulsory activation part of the system?	Not reported	Not reported
Maximum duration of unemployment benefits	Maximum duration depends on number of months in employment the past five years prior to spell: Under 36 months: 20 weeks 36 months or more: 30 weeks (p.1522)	In sample: Longest potential duration is 62 weeks (mean: 26,25 weeks) (p.110)
Destination	Employment	Reemployment (p.115)
Sampling frequency	Daily (p.1522)	Weekly (p.108).
Time interval the outcome measure is based on	Weekly (p.1537pp)	Weekly (p.141pp)
Sample size	650.922 unemployment spells (p.1523)	1.391 (p.108)
Are the labour market conditions described?	Institutional background briefly described on p. 1521	No
Censoring level	Specific levels not mentioned.	Not reported (p.104, 107)
Is there correction for censoring? (Yes/No, ref)	Yes (p.1530)	Not reported

**Table:** 

**Author**	**de Groot & van der Klaauw**	**Engberg**	**Farber & Valletta**
Title	The Effects of Reducing the Entitlement Period to Unemployment Insurance Benefits	Structural estimation of the impact of unemployment benefits on job search	Do Extended Unemployment Benefits Lengthen Unemployment Spells?
Year	2014	1990	2015
Country	Netherlands	USA	USA
Journal	IZA DP	Dissertation, University of Wisconsin‐Madison	*Journal of Human Resources*
Time period covered by data	July 2004 to December 2008	Middle 1970's (p.49)	2000‐2012
Type of unemployment scheme	Unemployment insurance	Unemployment insurance / Trade Adjustment Assistance Program (TAA)	Unemployment insurance
Type of data used	Administrative registers	Survey data (p.49)	Microdata (individual level) from monthly survey of household and individuals ‐ The Current Population Survey CPS (p.874). CPS data from January 2000 to February 2005 and from January 2007 to October 2012
Target group	Individuals with certain work histories	Workers who are eligible for unemployment benefits through TAA program or Unemployment insurance program. Recalled individuals or individuals who were out of the labour force at the end of the sample frame are excluded. (p.17,49)	Unemployed job losers ages 20‐64 (p.884+887)
Target group ‐ eligibility, requirements for benefits	Restrict to individuals entering unemployment between July 2004 and December 2008 who had worked at least 4 of the last 5 years and at least 26 weeks of the last 36 weeks. Exclude workers with an employment history of 10, 11 and 12 years because of additional reform changes for these groups (p. 8)	In order to be eligible for TAA, workers must be laid off by firms which are certified by the federal government to be adversely affected by increased import competition (p.7)	Eligible if persons have lost their job through no fault of their own and if they meet state specific requirements regarding work history and wage during a 12‐15 month period preceding their job loss. Availability for work and active job search are typically required, but vary across states (p.876)
Benefit level/replacement rate	Before the reform: 70 per cent of the minimum wage or 70 per cent of the last wage, whichever was lower.; after the reform: During the first two months of Unemployment insurance a worker receives 75 per cent of the past wage and afterwards 70 per cent (again capped at the maximum)	TAA+Unemployment insurance: 70% of previous wage ‐ but not to exceed average manufacturing wage (p.7). TAA benefits are, on average, almost 50% higher than Unemployment insurance benefits (p.15)	State dependent ‐ In most states replacement rate is about half of the claimant's pre separation weekly wage (p.876)
Benefit level/replacement rate available after exhaustion	Welfare is means tested and complements the household income to 50 per cent of the minimum wage for a unlimited time period.	Not reported	Not reported
Is compulsory activation part of the system?	Not reported	Not reported	Not reported
Maximum duration of unemployment benefits	Varies with work history, maximum is 60 months before reform, reduction range is ‐2 to +22 months	TAA: minimum of one year of income assistance payments (p.7). Last, on average, 5 weeks longer than Unemployment insurance (p.15)	Normally 26 weeks. This duration is supplemented and extended in periods of economic distress. Most recent extension happened in 2008 (up to 99 weeks in many states) (p. 876)
Destination	Employment	Not reported	Exits to employment Exits out of the labour force (p.891)
Sampling frequency	Monthly	Not reported	Monthly (p.874)
Time interval the outcome measure is based on	Monthly	0,1 = 2 weeks (p.110)	Monthly (p.890)
Sample size	Over 2.3 million spells	Entire sample, N=344 (p.110) TAA: 123 (p.111) Unemployment insurance: 221 (p.111)	January 2000 to February 2005: 75.089 monthly observations on 54.678 spells of unemployment January 2007 to October 2012: 126.062 monthly observations on 86.869 spells of unemployment (p. 887‐88)
Are the labour market conditions described?	Not reported	Not reported	Yes. Variation in total number of available Unemployment insurance weeks and unemployment insurance claims in the 2000's (p. 878‐79)
Censoring level	Not reported	Not reported	January 2000 to February 2005: 26.578 January 2007 to February 2012: 50.255 (p.888)
Is there correction for censoring? (Yes/No, ref)	Yes	Not reported	Yes (p.887)
**Author**	**Farber, Rothstein & Valletta**	**Ferrada**	**Figura & Barnichon**
Title	The Effect of Extended Unemployment Insurance Benefits: Evidence from the 2012‐2013 Phase‐Out	Introduction of Unemployment Insurance Savings Accounts in Chile: Job Search, Moral Hazard and Income Effects	The Effects of Unemployment Benefits on Unemployment and Labour Force Participation: Evidence from 35 Years of Benefits Extensions
Year	2015	2011	2014
Country	USA	Chile	USA
Journal	American Economic Review	Dissertation, University of Chicago	Board of Governors of the Federal Reserve System (U.S.). *Report*.
Time period covered by data	2008:1‐2014:6 (p.173)	1985‐2002	1976‐2012 (p.4)
Type of unemployment scheme	Unemployment insurance (+ EUC and EB)	Unemployment insurance (+ Unemployment insurance savings accounts) (p.14)	Unemployment insurance + EEB (p. 2)
Type of data used	Micro data from Current Population Survey (CPS) (p.172)	Matched data that combines survey (social Protection survey) and administrative data (Unemployment insurance office) (p.4, 14‐15)	Records from Employment and Training Administration(ETA) + CPS data (p.3‐4)
Target group	Individuals who have been unemployed for at least 3 full months (because of job loss) ages 18‐69 (p.172‐73)	Unemployed workers in the private sector over 18 years old. (p.17)	Permanent job losers over 20 years old (p.6)
Target group ‐ eligibility, requirements for benefits	Loss of job. Further requirements not described. (p. 173)	1985‐2002 (old system): involuntary dismissal, employed for 12 out of the last 24 months, offer labour to county, accept any job offered (p.7) 2002 (new system): registered in municipal employment office, employed for at least 12 months since affiliation to system for persons with indefinite contracts (6 months for temporary contracts). Complementary benefit from government only available for persons with indefinite contracts, who are separated involuntary from employment and have a low balance in individual savings account (p.8)	Unemployment insurance: Not described EEB: Unemployment groups to be affected by EEB are primarily people with greater than or equal to 18 weeks of unemployment (less than maximum allowable duration of regular Unemployment insurance benefits) and People with greater than the maximum allowable duration of regular Unemployment insurance benefits but less than 60 weeks of unemployment.
Benefit level/replacement rate	Not described.	Old system: Benefit independent of previous wage, decreasing replacement rate. 1985‐95: months 1‐3: $6000 (US$39,4); 4‐6: 2/3 of initial level; 7‐12: 1/3 of initial level 1995‐02: months 1‐3: $17,338 (US$42,2); 4‐6: 2/3 of initial level; 7‐12: 1/2 of initial level New system: Indefinite contracts before unemployment: The number of contributions made to individual savings account prior to unemployment determines the rate of benefits. For example between 12 and 18 contributions to savings account gives one benefit grant equal to the accumulated balance. More than 60 contributions results in 5 benefits, where the first one is equal to one fourth of the accumulated balance, the second one (third, fourth and fifth) equal to 90 % (80 %, 70% and 60 %) of the first benefit. (p.10). Temporary contracts before unemployment: One payment, which is equal to accumulated balance in individual savings account (p.12). (p.61)	Unemployment insurance: not described EEB: not described
Benefit level/replacement rate available after exhaustion	Not reported	Not reported	Individuals eligible for three months extra benefits, they haven't reached maximum weeks of federal benefits (p.38)
Is compulsory activation part of the system?	Not reported	No	Not reported
Maximum duration of unemployment benefits	From 26 weeks to 99 weeks. Regular benefits: 26 weeks. Emergency Unemployment. Compensation (EUC): 53 weeks. Extended benefits (EB): 20 weeks. State dependent (p. 172)	Old system: 12 months (p.7) New system: 5 months (persons with indefinite contracts) or 1 month (temporary contracts) (p.39)	Unemployment insurance: 26 weeks; EEB (extended and emergency): Extended benefits: 13 weeks; Emergency benefits (: tied to conditions of state ‐ typically 2‐3 years; see p. 33‐38
Destination	Exit to employment Exit from the labour force (p. 173)	Employment	Employment Non‐participation (p.19)
Sampling frequency	Monthly (p.173)	Monthly (p.16)	Monthly (p.4)
Time interval the outcome measure is based on	Monthly	Monthly (p.26)	Monthly
Sample size	56.491 monthly observations on 37.059 spells 2008‐2011: 25.021 spells; 2012‐2014: 12.324 spells. (p. 173)	Old system (Unemployment insurance): 6827 New system: 1685 (p.26)	Data divided into 5 groups (no EEB/EEB): (p. 28) Group 1: 106.972/101.652; Group 2: 48.724/59.376; Group 3: 22.531/32.967 Group 4: 29.828/54.144; Group 5: 10.945/46.011 Total: 513.150 (219.000/294.150)
Are the labour market conditions described?	Yes (median weeks Unemployment insurance available + Unemployed per vacancy) (p. 172)	Not reported	History of recession and benefit legislation described p. 33‐38
Censoring level	Not reported	Not reported	Not reported
Is there correction for censoring? (Yes/No, ref)	Not reported	Yes (p. 19pp)	Not reported

**Table:** 

**Author**	**Fitzenberger & Wilke**	**He**	**Hunter**
Title	New Insights into Unemployment Duration and Post Unemployment Earnings in Germany	Effects of unemployment insurance extensions on job search outcomes	An empirical investigation of the impact of unemployment insurance on unemployment duration and wages following unemployment
Year	2010	2013	1990
Country	Germany	USA	USA
Journal	Oxford Bulletin of Economics & Statistics	Dissertation, Clemson University	Dissertation, University of Wisconsin‐Madison
Time period covered by data	1975‐2001 (p.803)	2002‐2012	1979‐1980 (p.62)
Type of unemployment scheme	Unemployment insurance (UB)	Unemployment insurance	Unemployment insurance
Type of data used	Administrative data (p. 803)	Current Population Survey (CPS) (p. 9)	Survey data ‐ event history from the Job Search Assistance Research Program (p.62)
Target group	Restricted to West Germany, with spells beginning in 1996‐97. Sample restricted to men, age 26‐49. Further restrictions: Includes only unemployment spells which begin with UB and where there is no foregoing unemployment spell the year before the start of the current spell. (p. 805)	Individuals age 16‐65, not full‐time enrolled in school and actively looking for job the past 4 weeks (p.11)	Adult men aged 18‐65 (p.65). Persons who are self‐employed, institutionalized or away at schooled, are excluded (p.63)
Target group ‐ eligibility, requirements for benefits	Persons are eligible, if they have worked for at least 12 months in the past 3‐7 years (p.800)	Involuntary job loss Minimum working requirements are state dependent (p.7)	Must not have left previous job voluntarily. Participants must be available and actively seeking for a job (p.7)
Benefit level/replacement rate	63% without children 67% with children (p. 800)	Replacement rate varies by state (p.7)	Depends on previous work experience and earnings (p.9). Replacement rate frequently 1/26th of the highest quarter of earnings in the last five quarters ‐ benefits usually replace a quarter to half of weekly wage (p.8)
Benefit level/replacement rate available after exhaustion	UA (53 or 57 % replacement rate) or SB (replacement rate dependent on household composition). Both tax funded (p. 800‐801)	Not reported	Not reported
Is compulsory activation part of the system?	Not reported	Not reported	Unemployed are expected to be available and actively seeking for a job (p.7)
Maximum duration of unemployment benefits	Entitlement period depends on duration of socially insured employment during past 7 years and on the persons age: 42 years: up to 12 months; 42 years: up to 32 months. (p.800)	Regular Unemployment insurance: 26 weeks. Extended benefit: additional 13 weeks. Temporary extended unemployment compensation: 13 weeks (26 weeks in states with high employment). Emergency unemployment compensation: dependent on states. 14 weeks available for all states (p.7‐8)	Depends on previous work experience and earnings. Maximum duration is typically 26 weeks. Extended Benefit: extends duration by 50%, up to a maximum of 3 weeks. Federal Supplemental Benefit Program: increases eligibility up to 65 weeks (p.9)
Destination	Employment	Re‐employment (p.3)	Not reported
Sampling frequency	daily (p.803)	Monthly (p.9)	Semi‐monthly (two week periods) (p.63)
Time interval the outcome measure is based on	Daily (p. 814)	Monthly (p.8)	Semi‐months / weekly (p.99)
Sample size	UPIT (Unemployment with Permanent Income Transfers): 10.295; NE (Non‐Employment): 12.165 (p. 807). Total: 22.460	52.791 individuals (844.656 monthly observations) (p.11, 27)	Duration sample: 1.816 (p.64)
Are the labour market conditions described?	Not reported	Not reported	Not reported
Censoring level	UPIT: 23%; NE: 13% (p.807)	Not specified	Not reported
Is there correction for censoring? (Yes/No, ref)	Censored Box‐Cot Quantile Regression (p.794)	Yes (p. 10)	Yes. Avoidance of left censoring (p.66)
**Author**	**Lalive**	**Lalive & Zweimüller**	**Lalive, Landais & Zweimuller**
Title	Unemployment Benefits, Unemployment Duration and Post‐Unemployment Job. A Regression Discontinuity Approach	Benefit entitlement and unemployment duration: The role of policy endogeneity	Market externalities of large unemployment insurance extension programs
Year	2007	2004	2015
Country	Austria	Austria	Austria
Journal	AEA Papers and Proceedings	Journal of Public Economics	LSE Research Online Documents on Economics
Time period covered by data	1989‐1991	1986‐1998	1980‐2009
Type of unemployment scheme	Unemployment insurance	Unemployment insurance / REBP	Unemployment insurance (UB) + REBP (Regional Extended Benefit Program)
Type of data used	Administrative data (p.109)	Administrative data ‐ longitudinal individual data from 1) Austrian Social Security database 2) Austrian Unemployment Register (p.2593)	Administrative data + Austrian Social Security Database (ASSD) (p.9)
Target group	Individuals aged 46‐53 years at the beginning of spell, with a continuous work history (career with a ratio of actual to potential work experience of at least 0,7), and coming from the non‐steel sector (p.109)	Male workers aged 45‐54 at beginning of spell, who have a continuous work history since 1972 (p.2593).	All unemployed men aged 50‐54, at the start of the spell (p.9)
Target group ‐ eligibility, requirements for benefits	Work experience requirement (p.109)	Unemployment Insurance contributions must be paid, while employed (p.2590) REBP: a) Individuals must be 50 years or older, b) Have a continuous work history (780 employed weeks in the 25 years prior to spell), c) Residing in one of 28 selected areas at least 6 months prior to claim, d) start unemployment after or have an unemployment spell in progress in June 1988 (p.2592)	UB: Involuntary job loss (voluntary job loss or job loss because of misconduct have a 4 week quarantine period before being eligible) (p.7) REBP: 50 years or older, continuous work story (780 weeks employment last 25 years prior to current spell), residence in specific labour market districts at least 6 months prior to claim, start of unemployment/spell in progress after June 1988 (p.8)
Benefit level/replacement rate	Not mentioned.	Amount depends on previous earnings; family allowances are paid. In 1990 the replacement ratios were: Low income earner: 48,2%; Middle income earner: 40,4%; High income earner: 29,6%. (p.2591)	Depends on previous earnings 1990: median income earner: 40,4 % Low income earner: 48,2 % High income earner: 29,6% (p.7)
Benefit level/replacement rate available after exhaustion	Not reported	Notstandhilfe/”Transfer payments for those in need”: means‐tested benefit (individuals are only eligible if in trouble). Replacement rate can at most be 92% of UB payment (p.2591)	Post‐UB transfer: means‐tested. Maximum 92% replacement rate of UB 1990: median post‐UB transfer was about 70% of median UB (p.7)
Is compulsory activation part of the system?	Not reported	Not reported	Requirement: Searching actively for a job within qualification scope (p.7)
Maximum duration of unemployment benefits	Dependent on work experience and age (p.109) 40‐49: 39 weeks (if they have worked for 312 weeks within last 10 years prior to spell) 50‐59: 52 weeks (if they have worked for 468 weeks within last 15 years prior to spell) Regional Extended Benefit Program (REBP): 209 weeks (if they are aged 50 or older, have worked 78o weeks in the last 25 years, live in selected labour market districts, and unemployment spell has started after June 1988). (p.109)	Before August 1989: 30 weeks (if contributions have been made for 156 weeks out of the last 5 years) After August 1989: Potential duration becomes dependant on age as well. Age group 40‐49: maximum 39 weeks. Age group 50 and above: maximum of 52 weeks. REBP: Maximum of 209 weeks (p.2590)	Depends on work history and age and region, restrict to age 50 and older: treated regions 209 weeks and non‐treated regions 52 weeks (p.7)
Destination	Transition to regular job (81%) Long term sickness (5%) Early retirement programs (8.5%) (p.109)	Employment/regular jobs (p.2595, 2600)	Employment (p.3)
Sampling frequency	Daily (p.109)	Daily (p.2593)	Daily (specific dates of entry and exit observed) (p.9)
Time interval the outcome measure is based on	Weeks (p.110)	Weekly (p.2608)	Weekly (Table 4–5)
Sample size	40,028 individuals	Treated spells: 7,431 steel workers + 14,479 non‐steel workers; non‐treated spells: 23,774 steel workers and 266,392 non‐steel workers. Strategy 3 (the one used in analysis): 233,223 spells	106.164 spells
Are the labour market conditions described?	Not reported	Not reported	Structure and history of labour market described (p.7‐9)
Censoring level	1% censored at the end of observation window 4% censored due to break in unemployment spell exceeding 4 weeks (p.109)	1.9% are censored (p.2594) Spells ending with transition to retirement (2.2%), long‐term sickness (5.1%), and out of labour force (7.7%) are also treated as right censored (p.2600). Total: 16.9%	Not reported
Is there correction for censoring? (Yes/No, ref)	Not reported	Yes (p.2006p)	Not reported

**Table:** 

**Author**	**Landais**	**Lee & Wilke**	**Lubyova & van Ours**
Title	Assessing the Welfare Effects of Unemployment Benefits Using the Regression Kink Design	Reform of Unemployment Compensation in Germany: A Nonparametric Bounds Analysis Using Register Data	Unemployment dynamics and the restructuring of the Slovak unemployment benefit system
Year	2015	2009	1997
Country	USA	Germany	Slovakia
Journal	American Economic Journal: Economic Policy	ZEW (2005) + Journal of business and economic statistic (2009)	European Economic Review
Time period covered by data	1978‐1984 (p.19)	1975‐1997	1991/92, 1994/95
Type of unemployment scheme	Unemployment insurance	Unemployment insurance	Unemployment insurance
Type of data used	Administrative data from Continuous Wage and Benefit History (CWBH) Unemployment insurance records (p.19)	Administrative registers	Administrative registers
Target group	Not reported	Individuals aged 44 to 48 years as the reform affects individuals older than 42 and the group 42‐43 gets a short extension and therefore is a bad treatment group. (p. 195) The sample is restricted to males (p. 196)	Not reported
Target group ‐ eligibility, requirements for benefits	Not reported	An unemployed with sufficient amount of working experience. (p. 194)	Not reported
Benefit level/replacement rate	Not reported	Not reported	Before 1992: 65% of previous wage the first 6 months and 60% the last 6 months. After 1992: 60%/50% in the first/last 3 months. After 1995: Back to pre‐1992 level (p. 927)
Benefit level/replacement rate available after exhaustion	Not reported	Unemployment assistance ‐ depends on previous earnings and it is means tested. (p. 195)	Not reported
Is compulsory activation part of the system?	Not reported	Not reported	Not reported
Maximum duration of unemployment benefits	Not reported	Maximum length increased from 12 to 22 months for the treatment group ‐ remained constant for the control group (12 months). (p. 195)	Before 1992: 12 months. After 1992: 6 months. After 1995: 30 years=no change, 30‐45 years=8 months, >45 years=9 months (p. 927)
Destination	Job	No specific exit, though recalls are not considered (p.9)	Regular job or other reasons (e.g. subsidized jobs, retraining, school) (p. 928)
Sampling frequency	Weekly (p.20)	Daily (p. 195)	Monthly
Time interval the outcome measure is based on	Weekly (p.20)	Daily	Monthly
Sample size	(p.66)	In total: 4,049 spells, of which 2,922 are recorded during the pre‐reform period (p. 197)	1991/92: 10,790 observations. 1994/95: 18,603 observations (p. 927)
Are the labour market conditions described?	EB trigger dates described (p.65)	Not reported	Yes (p. 926)
Censoring level	(p.66)	Table 2 + 3 (p. 197)	Not reported
Is there correction for censoring? (Yes/No, ref)	Yes	Yes (p. 198)	Yes (p. 929)
**Author**	**Machikita, Kohara & Sasaki**	**Micklewright & Nagy**	**Nekoei & Weber**
Title	The Effect of Extended Unemployment Benefit on the Job Finding Hazards: A Quasi‐Experiment in Japan	Unemployment insurance and incentives in Hungary	Does Extending Unemployment Benefits Improve Job Quality?
Year	2013	1995	2015
Country	Japan	Hungary	Austria
Journal	IZA DP	EUI working papers in economics	IZA Discussion Papers
Time period covered by data	2005‐2006 (p.9‐10)	Spells starting in December 1992 and January 1993	1989‐2011 (p.6)
Type of unemployment scheme	Unemployment insurance	Unemployment insurance	Unemployment insurance
Type of data used	Administrative Data (p.9)	Administrative registers	Two sets of Administrative Data (The Austrian Social Security Database (ASSD); Austrian Unemployment Registers at the individual level (p.6)
Target group	Unemployment insurance recipients in the age 44‐46 (p.3)	No spells as the result of quit and receivement statutory severance pay (p. 7) Sample characteristics table 1 (p. 37)	Individuals who are eligible for Unemployment insurance, but do not take more than 28 days to claim Unemployment insurance ‐ and who are eligible for either 30 (39) weeks of Unemployment insurance if below (above) age 40. (p.6‐7)
Target group ‐ eligibility, requirements for benefits	Involuntary unemployed: 6 months of employment in the year prior to spell Voluntary unemployment: 1 year of employment in the year prior to spell. Waiting period of 90 days before being eligible (p.5)	At least 12 months of work is required in the previous 4 years in order to qualify for any benefits. (p. 4)	Minimum tenure of 28 weeks at pre‐unemployment job (p.6)
Benefit level/replacement rate	Depends on age at tenure in previous job: between 50‐80% of wage at previous job (p.4)	Before change: Period 1 (two‐thirds of the period) =70% of past earnings, period 2=50%. After the change: Period 1 (first quarter)=75%, period 2=60% (p. 5)	55 % of previous earnings. Maximum/minimum levels adjusted annually (p.6)
Benefit level/replacement rate available after exhaustion	Once the cumulative unemployment spells leaving the previous job exceed 360 days, the unemployed cannot receive benefits (p. 4)	Social Benefit= flat‐rate equal to cut‐off line (two‐thirds of minimum wage) (p. 6)	Means‐tested unemployment assistance (p.6)
Is compulsory activation part of the system?	Unemployed must go to public service employment office every four weeks to receive advice (p.5).	Not reported	Not reported
Maximum duration of unemployment benefits	Between 90‐360 days (p.4) Involuntary workers: Depends on age and job tenure. Voluntary workers: no discrimination by age, only job tenure. (p. 5)	Depends on the working experience. Before the change: Min. = 4½ months, Max. =18 months. After the change: Min. =3 months, Max. =12 months. (p. 4)	Depends on previous work experience and age. Baseline: 20 weeks 30 weeks if persons have been employed for 3 years out of 5 years prior to spell. Workers above 40 eligible for 39 weeks, if they have been in employment in 6 out of the last 10 years prior to spell (p.6)
Destination	Employment (p.9‐10)	Job, a government labour market programme, exhaustion of entitlement, or other reasons (p. 13) See Table 3 for potential exits (p.39)	Employment
Sampling frequency	Daily (p.3)	Monthly	Daily (p. 6)
Time interval the outcome measure is based on	Daily (p.11)	Monthly	Daily /weekly (Figure VII + VIII + Table 1)
Sample size	14,057 (p.3)	92 scheme=50,441 spells, 93 scheme=30,270 spells (p. 8)	For 10/5/2 years bandwidth: 1,589,180/827,736/335,141 spells
Are the labour market conditions described?	Not reported	A little (p. 1)	Not reported
Censoring level	Not reported	Table 3 (p. 39)	Spells censored at 2 years (p.9)
Is there correction for censoring? (Yes/No, ref)	Not reported	Yes	Yes
**Author**	**Newton.& Rosen**	**Puhani**	**Rebollo‐Sanz & García‐Pérez**
Title	Unemployment insurance, income taxation and duration of unemployment	Poland on the dole: The effect of reducing the unemployment benefit entitlement period during transition	Are Unemployment Benefits harmful to the stability of working careers? The case of Spain
Year	1979	2000	2015
Country	USA	Poland	Spain
Journal	Southern Economic Journal	Journal of Population Economics	Studies on the Spanish Economy
Time period covered by data	1974‐1976	1991/1994	1995‐2007 (p. 8)
Type of unemployment scheme	Unemployment insurance	Unemployment Benefit	Unemployment insurance (UIS)
Type of data used	Administrative Data ‐ Random samples drawn out from Georgia state's claim file (p.777)	Survey data from the Polish Labour Force Survey (p.38)	Administrative Data ‐ Longitudinal Working Lives Sample (LWLS) (p. 7)
Target group	Individuals who are drawing unemployment benefits from Georgia, and have filed a valid claim (p.777)	Currently unemployed aged 18‐55, who state that they are looking for a job (p.38, 42)	Spanish male workers aged 18‐55 years (p.8)
Target group ‐ eligibility, requirements for benefits	An individual is eligible, if the person has worked in a covered job for two or more quarters of the year previous to job termination. Also depends on the conditions under which the employment relation was terminated (p.776)	Unemployed has to register at labour office as unemployed (p.36)	All individuals (under age 64) who are involuntarily unemployed, provided they have been employed for the last 12 months over the previous 72 months prior to spell (p.4)
Benefit level/replacement rate	The replacement rate is a function of high quarter earnings ‐ the function is not fixed, but varies with earning‐levels (p.776)	Before reform: First 3 months: 70% of most recent wage 3rd‐9th month: rate declining to 50 % Thereafter: rate declining to 40 % After reform: Flat replacement rate at 36% (p.36)	First 6 months: 70% replacement rate Seventh month and onward: 60 % replacement rate (p.5)
Benefit level/replacement rate available after exhaustion	Not reported	Not reported	Unemployment Assistance (p.5) Unemployment Assistance benefits are available for people who have not been working long enough to qualify for the previously described unemployment benefits and for people who have exhausted them and have family responsibilities. (p. 5)
Is compulsory activation part of the system?	Not reported	Formally, individuals could be excluded from benefits, if they rejected more than two job offers/enrolment in active labour market schemes. But labour offices were very generous in this respect (p.36)	Not reported
Maximum duration of unemployment benefits	Maximum duration depends on weekly benefit amount and total earnings in the base year (p.776)	Before December 1991: Unlimited After December 1991: 12 months (with few exceptions) (p.36)	Entitlement depends on previous employment duration. Initial benefit period is 4 months, which can be extended in intervals of 2 months up to potentially 2 years (p.5)
Destination	Not reported	Not reported	Employment; Job‐to‐Job transition to permanent contract (JJ‐PC); Job‐to‐Job transition to temporary contract (JJ‐TC). (p.7)
Sampling frequency	Not reported	Not specified.	Daily? Data compiled annually (p.7) Data compiled annually, but seems to be measured daily (p. 7)
Time interval the outcome measure is based on	Weekly (p.779)	Monthly (p.40‐41)	Monthly (p.9)
Sample size	627 observations (p.777)	Men: 4.353 Women: 4.441 Total: 8794 (p.38)	Sample size, workers: 193.797 (p.9)
Are the labour market conditions described?	Not reported	Labour market developments/unemployment rates briefly described (p.36‐37)	No
Censoring level	Specific level not reported, censored spells excluded (p.777)	Not reported	Employment spells: 22,93% Unemployment spells: 6,81% (p.9)
Is there correction for censoring? (Yes/No, ref)	Not reported	Not reported	Yes (p.14)
**Author**	**Rothstein**	**Schmieder, von Wachter & Bender**	**Schmieder, von Wachter, Bender**
Title	Unemployment Insurance and Job Search in the Great Recession	The Effects of Extended Unemployment Insurance over the Business Cycle: Evidence from regression discontinuity estimates over 20 years	The Effect of Unemployment Benefits and Nonemployment Durations on Wages
Year	2011	2012	2016
Country	USA	Germany	Germany
Journal	Brookings Papers on Economic Activity	Quarterly Journal of Economics	American Economic Review
Time period covered by data	2004‐2011	Spells starting any time between July 1987 and March 1999 (follow until 2008)	Spells starting any time between 1987 and 1999 (follow until 2008)
Type of unemployment scheme	Unemployment insurance	Unemployment insurance	Unemployment insurance
Type of data used	Current Population Survey (CPS) (p.160)	Administrative data (social security records) (p.703)	Administrative registers
Target group	Unemployed workers, who were interviewed between May 2004‐January 2011, and matched to interviews the following 2 months (p.163‐64)	All nonemployment spells for persons aged 40‐49 years, starting between 1987 and 1999. (p.713) Restricted further to persons, who have been working at least 52 months in the last 7 years prior to spell, and who have not received Unemployment insurance benefits in that time period. (p.714)	Only fresh spells (of those aged 40‐46) with max. entitlement for their age group who involuntary quit (employed at least 36 months (44 months for the 44 age cut off) the last 7 years.
Target group ‐ eligibility, requirements for benefits	Unemployed, meaning people who are available to start work, and have actively looked for work at least once in the last 4 weeks. (p.163)	Loss of job through no fault of their own (p.711)	Loss of job through no fault of their own
Benefit level/replacement rate	Not reported	63% of previous net earnings for a person without children (p.711)	63% of previous net earnings for a person without children
Benefit level/replacement rate available after exhaustion	After 2008 13 weeks of EUC benefits were made available to anyone who exhausted regular benefits before march 29 2009. The program was subsequently expanded in November 2008. That expansion extended the original EUC (now called EUC tier I) benefits to 20 weeks and added a second tier of 13 weeks of benefits in states with unemployment rates above 6 per cent. A second expansion in November 2009 changed tier II benefits to 14 weeks and added tier III, 13 weeks of benefits in states with unemployment rates above 6 per cent, and tier IV, an additional 6 weeks in states with unemployment rates above 8.5 per cent Individuals in states qualifying for all four tiers were thus eligible for 53 weeks of EUC benefits. The first four columns of table 1 show the number of tiers and number of weeks available over time (p. 150).	Unemployment Assistance (UA): replacement net rate is 53%, but depends on spousal earnings also. Average replacement rates for women/men: 10%/35%. (p.713)	Unemployment Assistance (UA): replacement net rate is 53%, but depends on spousal earnings also. Average replacement rates for women/men: 10%/35%.
Is compulsory activation part of the system?	To be unemployed people have to have actively looked for work at least once in the last 4 weeks	Not reported	Not reported
Maximum duration of unemployment benefits	Potential duration up to 99 weeks in certain states (26 Unemployment insurance + 53 EUC + 20 EB) (p.153)	Duration tied to age and labour market history (p.711). Restrict analysis to those with max. Entitlement. At age cut 42 entitlement is increased from 12 months to 18 months	Duration tied to age and labour market history. Restrict analysis to those with max. Entitlement. At age cut 42 entitlement is increased from 12 months to 18 months (and at 44 from 18 to 22 months, do not use)
Destination	To employment; out of labour force (p.163)	Outcome measured: Nonemployment duration	Outcome measured: Nonemployment duration
Sampling frequency	Monthly (p.160)	Daily	Daily
Time interval the outcome measure is based on	Weekly (p.163)	Monthly (722pp.)	Monthly
Sample size	All unemployed workers: 95485 (Job losers), 77913 (job leavers, entrants and re‐entrants). Subsample with two or more follow‐up interviews: 77813 (Job losers), 61105 (Job leavers, entrants, and re‐entrants) (p.163)	Overall 1,990,812 (p.750). For bandwidth 0.2 years: 134,371 spells in total	437,899 spells for age cut 42
Are the labour market conditions described?	yes (p.145‐155)	Not reported	Not reported
Censoring level	censoring at 99 weeks (p.165)	All nonemployment durations capped after 36 months. (p.714)	All nonemployment durations capped after 36 months. (p.749)
Is there correction for censoring? (Yes/No, ref)	Yes (p.165)	Not reported	Not reported

**Table:** 

**Author**	**U.S. Department of Labor**	**Valletta**	**Van Ours & Vodopivec**
Title	The effect of the duration of unemployment benefits on work incentives: an analysis of four data sets	Recent extensions of U.S. unemployment benefits: search responses in alternative labour market states	Does reducing unemployment insurance generosity reduce job match quality
Year	1995	2014	2008
Country	USA	USA	Slovenia
Journal	Unemployment Insurance Occasional Paper	Federal Reserve Bank of San Francisco, Working Paper Series	Journal of Public Economics
Time period covered by data	1978‐1983	2000‐2011. (2000‐2004; 2007‐2011)	Mid‐1997 to end‐2001
Type of unemployment scheme	Unemployment insurance	Unemployment insurance	Unemployment insurance
Type of data used	Administrative registers	Administrative data (from U.S. Department of Labor) + CPS data (6)	Administrative data (p.687)
Target group	Table III.1 (p. 33)	Unemployed individuals aged 16 and over + individuals who are in their first or second month of unemployment (p.11‐12)	Individuals with 1‐20 years of work experience (p.687)
Target group ‐ eligibility, requirements for benefits	Not reported	Job loss through no fault of their own. Meeting state minimum requirements regarding work history and wages in the 12‐15 months preceding the job loss (p.4).	To qualify for benefits, workers must be enrolled in a social insurance program (all formal sector workers covered) and job loss must be involuntary (p.685)
Benefit level/replacement rate	Mean net replacement rate: men=0.50, women=0.79 (p. 33)	Not reported	Dependent on previous wage. First 3 months: 70%. After the first three months: 60% (p.685).
Benefit level/replacement rate available after exhaustion	Not reported	Not reported	Unemployment assistance: Means‐tested benefit (p.686)
Is compulsory activation part of the system?	Not reported	Availability for work + active job search, but rules varies across states (p.4)	Yes. Individuals are expected to be capable of, available for, and looking for work in order to maintain entitlement (p.685)
Maximum duration of unemployment benefits	26 weeks but the program FSB extends benefit duration up to 65 weeks and EB extends up to 39 weeks (p. 2)	Normal Unemployment insurance‐benefits: 26 weeks Maximum duration in time period covered by data: 72 weeks (00‐04) / 99 weeks (08‐12) (p.5‐6)	Dependent on work experience. Duration between 3‐24 months (p.685) Maximum duration before/after reform (months): 1‐2,5 years: 3/3; 2,5‐5 years: 6/3; 5‐10 years: 9/6 10‐15 years: 12/6; 15‐20 years: 18/9 (p.687)
Destination	Not reported	Exit to employment. Exit to not in the labour force (p.39).	Employment (p.688)
Sampling frequency	Not reported	Weekly and Monthly (p.)	Daily (p.688)
Time interval the outcome measure is based on	Not reported	Monthly (p.6)	Monthly (p.687)
Sample size	5,167 men, 2,902 women (p. 32)	2000‐2004: Unemployment insurance‐eligible: 43.167; Unemployment insurance‐ineligible: 40.129. 2007‐2011: Unemployment insurance‐eligible: 72,347; Unemployment insurance‐ineligible: 50.141 (p. 39)	In total 8627 men and 9074 women and wage equations: 4176 men and 4217 women
Are the labour market conditions described?	Not reported	Yes, see table 1 for information about the timeline for extended programs (p.39)	Not reported
Censoring level	Not reported	Table 2 (p.39)	Censoring at December 31, 2001. Specific levels not found (p.688)
Is there correction for censoring? (Yes/No, ref)	No		Yes

**Table:** 

**Author**	**Van Ours & Vodopivec**	**Winter‐Ember**	**Wolff**
Title	How Shortening the Potential Duration of Unemployment Benefits Affects the Duration of Unemployment: Evidence from a Natural Experiment	Potential Unemployment Benefit Duration and Spell Length: Lessons from a Quasi‐experiment in Austria	The Hungarian Unemployment Insurance Benefit System and Incentives to Return to Work
Year	2006	1998	1997
Country	Slovenia	Austria	Hungary
Journal	Journal of Labor Economics	Oxford Bulletin of Economics and Statistics	The William Davidson Institute
Time period covered by data	August 1, 1997‐ December 31, 1999	1986‐1991	December 1992 ‐ January 1993 (p. 5)
Type of unemployment scheme	Unemployment insurance	Unemployment insurance (UB)	Unemployment insurance
Type of data used	Administrative registers	Administrative data ‐ 2 per cent sample from Austrian social security records (p.3)	Administrative registers
Target group	Not reported	Treatment group: Workers aged 50‐65 years Control group: Workers below age 50 (p.37)	Table 3 p. 33
Target group ‐ eligibility, requirements for benefits	Has to register with an unemployment office and depends on work history (p. 355)	Dependent on previous work experience and age. Voluntary quitters subject for a four week waiting period, before being eligible (p.35)	The individuals are required to have worked for at least one year during the previous 4 years. Additionally the individuals were supposed to search actively after a job, to accepts suitable jobs and to co‐operate with the labour‐centre (p.2)
Benefit level/replacement rate	Earnings related (p. 354)	45% of gross monthly earnings + allowances for dependants (p.35)	Before the change: First phase = 70 %, second phase= 50 %. After the change: First phase=75 %, second phase= 65 % (p. 31)
Benefit level/replacement rate available after exhaustion	UA ‐ means‐tested (p. 355)	Unemployment Assistance: 92% of UB and is means‐tested (.35)	Social benefits: 80 % of the minimum pension (p. 3)
Is compulsory activation part of the system?	Not compulsory, but unemployed has the opportunity to participate in ALMP activities. (p. 355)	Unreasonable rejection of job offer can result in UB being withheld for four weeks (p.36)	Not compulsory (p. 4)
Maximum duration of unemployment benefits	Before the reform: 3 months with 0‐1.5 years of experience, 6 months with 1.5‐5, 9 months with 5‐10 years, 12 months with 10‐15 years and 18 months with 15‐20 years of work experience. After the reform: 3months with 0‐5, 6 months with 5‐15 and 9 months with 15‐20 (p. 361)	Minimum: 12 weeks. Maximum: 52 weeks (for individuals over age 50, who have worked for at least 48 weeks in the last 15 years) (p.35) Maximum after June 1st, 1988: 209 weeks (if individuals are over age 50; have worked for 780 in the last 25 years; live and work in specific counties; have not quitted voluntarily (p.36)	Ten different entitlement periods, depending on the employment record. Before the change: 135‐540 days. After the change: reduced by one third. (p. 2)
Destination	The study distinguishes between job finding and other exit destinations. (p. 358)	All exits. Exit into employment. Exit into recall. Exit into retirement. Exit out‐of‐labour‐force. (p.42)	Employment, subsidised employment, training + retraining and (early) retirement. (p. 5)
Sampling frequency	Daily	Daily (p.37)	Duration of spells measured in days (p. 5)
Time interval the outcome measure is based on	Monthly	Daily (p.37)	Monthly
Sample size	9,196 males, 10,853 females (p. 358)	77,837 unemployment spells (p.37)	54,911 male spells, 25,200 women spells. 37.4 % (=20,519 obs.) of men and 38.1 % (=9,591 obs.) of women are administered by the 1993 benefit provisions. (p. 5)
Are the labour market conditions described?	Since 1995, its unemployment rate has remained remarkably stable, at a level of 6% ‐ 7%. (p. 354)	Not reported	Not reported
Censoring level	Not reported	N censored: 7937 (10.2%) (p.40)	39‐49% (p. 32)
Is there correction for censoring? (Yes/No, ref)	Yes (p. 371)	Yes (p.37)	Yes (p. 10)

#### 14.1.2 Numeric data for studies with effect estimate

**Table:** 

**Author**	**Barbanchon**	**Caliendo, Tatsiramos, Uhlendorff**	**Card, Chetty & Weber**
**Year**	2016	2013	2007
**Country**	France	Germany	Austria
**Journal**	Labour Economics	Journal of Applied Econometrics	The Quarterly Journal of Economics
**Type of outcome**	Time‐to‐Event	Time‐to‐Event	Time‐to‐event
**Outcome (there may be more than one, record them all)**	Hazard ratio to job, survival probability (8 months) in job and wage	Hazard ratio to job	Hazard ratio to job and secondary outcomes (wage growth and job leaving hazard rate)
**Time Point (s) (record the exact time, there may be more than one, record them all)**	Overall and divided into three time intervals	Overall and per month	First 0‐20 weeks of unemployment
**Source (questionnaire, admin data, other(specify) or unclear)**	Administrative data	Administrative data	Administrative data
**Valid Ns (only applicable for continuous outcome data). Mention treatment and comparison.**	17,000 but reduces to 8,352 for bandwidth 4 months	2241 males (1147 with short duration and 1094 with long) and 2776 females (1442 with low duration and 1334 with long)	Total 650922 job losses, 66.4% eligible for extended benefits. 565835 (509355) used for analysis
**Method of estimation**	Cox proportional	Proportional hazard rate with piecewise‐constant baseline hazard and a bivariate (unemployment and employment) in JA	Cox hazard rate with unrestricted daily baseline hazard. Censored at 140 days (20 weeks). SE's clustered by person
**Statistics (risk ratio, odds ratio, standard error, 95 cf, DF, p‐value, chi2)**	Hazard ratio (SE)	Hazard ratio (SE) separated on gender	Hazard ratio (SE) of extended benefits, effect on first 20 weeks
**Page numbers and notes**	Table 2.	Bivariate model in JA. Table 5 for overall effect and table 6 for time‐varying (per month) effects. Both includes employment exit too, unclear how it should be interpreted. Use WP table 4. Dummy coefficients (T/C) interacted with duration (standard error), sample B (fresh spells), table 4 p. 22 in WP (+employment hazard table 5 p. 23). Negative coefficients as treated (entitlement of 18 months) are compared to controls (entitlement of 12 months).	Table 2 p. 1541 column 3
**Notes**	Use only bandwidth 4 month	Monthly, have daily data	Secondary outcomes available (log wage growth and change in job leaving hazard rate over first five years of next job)
**Author**	**Lalive**	**Lalive & Zweimüller**	**Lalive, Landais & Zweimuller**
**Year**	2007	2004	2015
**Country**	Austria	Austria	Austria
**Journal**	American Economic Review	Journal of Public Economics	American Economic Review
**Type of outcome**	Means	Time‐to‐event	Means
**Outcome (there may be more than one, record them all)**	Mean duration in weeks until job exit. Change in earnings	Hazard ratio to job	Unemployment duration (the duration of paid unemployment recorded in the Unemployment insurance administrative data) and non‐employment duration (duration between two employment spells)
**Time Point (s) (record the exact time, there may be more than one, record them all)**	Overall	Overall	NR
**Source (questionnaire, admin data, other(specify) or unclear)**	Administrative data	Administrative data	Administrative data
**Valid Ns (only applicable for continuous outcome data). Mention treatment and comparison.**	40,028 individuals	Treated spells: 7,431 steel workers + 14,479 non‐steel workers; non‐treated spells: 23,774 steel workers and 266,392 non‐steel workers. Strategy 3 (the one used in analysis): 233,223 spells	106.164 spells
**Method of estimation**	Regression	Diff‐in‐diff‐in‐diff Cox proportional hazard rate	Regression
**Statistics (risk ratio, odds ratio, standard error, 95 cf, DF, p‐value, chi2)**	Effect on mean duration until job exit in weeks (SE)	Coefficients and SE adjusted for region cluster	Regression coefficients on mean duration (SE clustered at the yearXregion level)
**Page numbers and notes**	Table 1	Table 3 page 2604	Table 2
**Notes**	Use measure C	Note 4 different strategies (samples) to choose between	Non‐employment duration means conditioning the sample to those who found a job
**Author**	**Nekoe & Weber**	**Schmieder, von Wachter, Bender**	**Schmieder, von Wachter, Bender2**
**Year**	2015	2012	2016
**Country**	Austria	Germany	Germany
**Journal**	IZA DP	Quarterly Journal of Economics	American Economic Review
**Type of outcome**	Time‐to‐Event	Duration mean	Duration mean
**Outcome (there may be more than one, record them all)**	Exit to job	The duration of nonemployment is measured as the time between the start of receiving Unemployment insurance benefits and the date of the next registered employment spell. Since some people take many years before returning to registered employment and others never do so, we cap nonemployment durations at 36 months and set the duration of all longer spells at this cap.	The duration of nonemployment is measured as the time between the start of receiving Unemployment insurance benefits and the date of the next registered employment spell. Since some people take many years before returning to registered employment and others never do so, we cap nonemployment durations at 36 months and set the duration of all longer spells at this cap.
**Time Point (s) (record the exact time, there may be more than one, record them all)**	First 30/39 weeks.	Overall	Overall
**Source (questionnaire, admin data, other(specify) or unclear)**	Administrative data	Administrative data	Administrative data
**Valid Ns (only applicable for continuous outcome data). Mention treatment and comparison.**	For 10/5/2 years bandwidth: 1,589,180/827,736/335,141	Overall 1,990,812 (p.750). For bandwidth 0.2 years and 42 age cut off (the only one we use): 45,301 spells	437,899 spells for age cut 42
**Method of estimation**	Unclear, it is hazard ratios but no information other than that is given	Regression (effect on duration)	Regression (effect on duration)
**Statistics (risk ratio, odds ratio, standard error, 95 cf, DF, p‐value, chi2)**	Regression discontinuity coefficients with SE (non‐employment duration (censored at 2 years) also available) for different duration intervals (within 30 weeks and 39 weeks)	Regression coefficients on mean non‐employment duration (SE) with different bandwidths (use bandwidth 0.2 years) and SD	Regression coefficients on mean non‐employment duration (SE)
**Page numbers and notes**	Table 2 (IZA, bandwidth 10 years) and Table A1.2 (dissertation, several bandwidths)	Table 2 p. 723 and table W‐1 in the online appendix	Table 1, p. 750
**Notes**		Table 4 p. 22 (+employment hazard table 5 p. 23)	
**Author**	**Van Ours & Vodopivec**		
**Year**	2008		
**Country**	Slovenia		
**Journal**	Journal of Public Economics		
**Type of outcome**	Probability that the job found is a permanent job /job loss within a year; Hazard ratio		
**Outcome (there may be more than one, record them all)**	Wage growth, permanent job, job loss within one year, exit rate from job		
**Time Point (s) (record the exact time, there may be more than one, record them all)**	Within a year		
**Source (questionnaire, admin data, other(specify) or unclear)**	Administrative data		
**Valid Ns (only applicable for continuous outcome data). Mention treatment and comparison.**	In total 8627 men and 9074 women and wage equations: 4176 men and 4217 women		
**Method of estimation**	Not mentioned other than linear probability model for finding and loosing job, simple log wage regression and proportional Hazard ratio model		
**Statistics (risk ratio, odds ratio, standard error, 95 cf, DF, p‐value, chi2)**	Secondary outcome (loose job) within a year Hazard ratio (t‐value)		
**Page numbers and notes**	Table 3: log wage model results (with absolute t‐values based on robust standard errors); table 4 probability (and t‐value) of finding a permanent job/loose it within a year; table 5 Hazard ratio of job separation		
**Notes**	Use only Hazard ratio		

#### 14.1.3 Numeric data for studies without effect estimate/too high risk of bias

**Table:** 

**Author**	**Addison & Portugal**	**Ahn & Ugidos‐Olazabal**	**Amarante, Arim & Dean**
**Year**	2008	1995	2013
**Country**	Portugal	Spain	Uruguay
**Journal**	Economic Letters	Oxford Bulletin of Economics and Statistics	Desarrollo y Sociedad
**Type of outcome**	Time‐to‐event	Time‐to‐event	Mean
**Outcome (there may be more than one, record them all)**	Hazard ratio to job	Hazard ratio to job	Mean unemployment duration
**Time Point (s) (record the exact time, there may be more than one, record them all)**	Not reported	Overall	Overall
**Source (questionnaire, Administrative data, other(specify) or unclear)**	the Portuguese Quarterly Employment Surveys for the period 1992(2) to 1997(4)	Questionnaire	Administrative data
**Notes**	Too high risk of bias	Too high risk of bias	Too high risk of bias

**Table:** 

**Author**	**Arntz, Simon & Wilke**	**Arranz, Bulló & Muro**	**Belzil**
**Year**	2014	2008	1995
**Country**	Germany	Spain	Canada
**Journal**	Empirical Economics	Instituto de Estudios Fiscales	The Review of Economics and Statistics
**Type of outcome**	Duration	Time‐to‐Event	Time‐to‐event
**Outcome (there may be more than one, record them all)**	Bounds for the destination‐specific (to employment) cumulative incidence curve (figure only)	Hazard ratio to job	Hazard ratio to job
**Time Point (s) (record the exact time, there may be more than one, record them all)**	Any time	Overall	Overall
**Source (questionnaire, Administrative data, other(specify) or unclear)**	Administrative data	Administrative data	Administrative data
**Notes**	Do not provide effect estimate or data that enables calculation of effect estimate	Too high risk of bias	Too high risk of bias
**Author**	**Bover, Arellano & Bentolila**	**Chang**	**de Groot & van der Klaauw**
**Year**	2002	2010	2014
**Country**	Spain	USA	Netherlands
**Journal**	The Economic Journal	Dissertation, The Johns Hopkins University	IZA DP
**Type of outcome**	Time‐to‐event	Time‐to‐event	Time‐to‐event
**Outcome (there may be more than one, record them all)**	Hazard ratio to job	Hazard ratio to job	Hazard ratio to job
**Time Point (s) (record the exact time, there may be more than one, record them all)**	Overall	Overall	Within 3,6,12 and 18 months
**Source (questionnaire, Administrative data, other(specify) or unclear)**	Questionnaire	Questionnaire	Administrative data
**Notes**	Too high risk of bias	Too high risk of bias	Too high risk of bias
**Author**	**Engberg**	**Farber & Valletta**	**Farber, Rothstein & Valletta**
**Year**	1990	2015	2015
**Country**	USA	USA	USA
**Journal**	Dissertation, University of Wisconsin‐Madison	Journal of Human Resources	American Economic Review
**Type of outcome**	Time‐to‐event	Probability of finding a job	Time‐to‐event
**Outcome (there may be more than one, record them all)**	Hazard ratio to job	Exit to job	Hazard ratio to job
**Time Point (s) (record the exact time, there may be more than one, record them all)**	Overall	From 6 months of unemployment and forward (the month they are observed, it is not a duration model)	From 3 months of unemployment and forward (restrict to those who have been unemployed for at least 3 months)
**Source (questionnaire, Administrative data, other(specify) or unclear)**	Questionnaire	Questionnaire (the Current Population Survey, or CPS)	Questionnaire (the Current Population Survey, or CPS)
**Notes**	Too high risk of bias	Too high risk of bias	Too high risk of bias
**Author**	**Ferrada**	**Figura & Barnichon**	**Fitzenberger & Wilke**
**Year**	2011	2014	2010
**Country**	Chile	USA	Germany
**Journal**	Dissertation, University of Chicago	Board of Governors of the Federal Reserve System (U.S.). Report.	Oxford Bulletin of Economics & Statistics
**Type of outcome**	Time‐to‐event	Time‐to‐Event	Duration mean
**Outcome (there may be more than one, record them all)**	Exit to job	Exit to job	Mean duration until job exit.
**Time Point (s) (record the exact time, there may be more than one, record them all)**	Overall	5 duration groups	Overall, divided into quantile
**Source (questionnaire, Administrative data, other(specify) or unclear)**	Questionnaire and administrative data	Questionnaire (the Current Population Survey, or CPS)	Administrative data
**Notes**	Too high risk of bias	Too high risk of bias	Too high risk of bias
**Author**	**He**	**Hunter**	**Landais**
**Year**	2013	1990	2015
**Country**	USA	USA	USA
**Journal**	Dissertation, Clemson University	Dissertation, University of Wisconsin‐Madison	American Economic Journal: Economic Policy
**Type of outcome**	Time‐to‐event	Time‐to‐event	Duration elasticity
**Outcome (there may be more than one, record them all)**	Hazard ratio to job	Hazard ratio to job	Non‐employment duration
**Time Point (s) (record the exact time, there may be more than one, record them all)**	NR	Overall	NR
**Source (questionnaire, Administrative data, other(specify) or unclear)**	Questionnaire	Questionnaire	Administrative data
**Notes**	Too high risk of bias	Too high risk of bias	Too high risk of bias
**Author**	**Lee & Wilke**	**Lubyova & van Ours**	**Machikita, Kohara & Sasaki**
**Year**	2009	1997	2013
**Country**	Germany	Slovakia	Japan
**Journal**	Journal of business and economic statistic	European Economic Review	IZA DP
**Type of outcome**	Survival probability	Time‐to‐Event	Time‐to‐event
**Outcome (there may be more than one, record them all)**	Bounds for survival probability in unemployment (figure only)	Hazard ratio to job	Hazard ratio to job
**Time Point (s) (record the exact time, there may be more than one, record them all)**	Any time	Every two months period and in addition an overall (I guess that is how it works, although probably not intended)	Overall
**Source (questionnaire, Administrative data, other(specify) or unclear)**	Administrative data	Administrative data	Administrative data
**Notes**	Do not provide effect estimate or data that enables calculation of effect estimate	Too high risk of bias	Too high risk of bias
**Author**	**Micklewright & Nagy**	**Newton & Rosen**	**Puhani**
**Year**	1995	1979	2000
**Country**	Hungary	USA	Poland
**Journal**	EUI working papers in economics	Southern Economic Journal	Journal of Population Economics
**Type of outcome**	Time‐to‐Event	Mean	Time‐to‐event
**Outcome (there may be more than one, record them all)**	Non‐parametric hazard rate for two groups (separate) with different entitlement, figures only	Duration	Exit to job
**Time Point (s) (record the exact time, there may be more than one, record them all)**	Any time	Overall	Three months intervals
**Source (questionnaire, Administrative data, other(specify) or unclear)**	Administrative data	Administrative data	Questionnaire with retrospective questions
**Notes**	Do not provide effect estimate or data that enables calculation of effect estimate	Too high risk of bias	Too high risk of bias
**Author**	**Rebollo‐Sanz & García‐Pérez**	**Rothstein**	**U.S. Department of Labor**
**Year**	2015	2011	1995
**Country**	Spain	USA	USA
**Journal**	Studies on the Spanish Economy	NBER Working Papers	Unemployment Insurance Occasional Paper
**Type of outcome**	Exit probability	Time‐to‐event	Time‐to‐event
**Outcome (there may be more than one, record them all)**	Probability of exit to job	Difference in hazard rates to job, in percentage points	Hazard ratio to job
**Time Point (s) (record the exact time, there may be more than one, record them all)**	In month 1, 4 8 and 12	Overall	Overall
**Source (questionnaire, Administrative data, other(specify) or unclear)**	Administrative data	Questionnaire (the Current Population Survey, or CPS)	Four data sets: 1) Continuous Wage and Benefit History (CWBH) administrative; 2)Job Search Assistance Research Project (JSARP) survey, 1979‐1981; 3) Federal Supplemental Benefit (FSB) follow‐up, survey, 1975‐1978; 4) Newton‐Rosen, admin, 1974‐1975. Only 2) is relevant
**Notes**	Too high risk of bias	Too high risk of bias	Too high risk of bias
**Author**	**Valletta**	**Van Ours & Vodopivec**	**Winter‐Ember**
**Year**	2014	2006	1998
**Country**	USA	Slovenia	Austria
**Journal**	Federal Reserve Bank of San Francisco, Working Paper Series	Journal of Labor Economics	Oxford Bulletin of Economics and Statistics
**Type of outcome**	Time‐to‐event	Time‐to‐Event	Time‐to‐Event
**Outcome (there may be more than one, record them all)**	Hazard ratio to job	Hazard ratio to job	Hazard ratio to job
**Time Point (s) (record the exact time, there may be more than one, record them all)**	Overall and separate for more/less than 26 weeks (where ordinary Unemployment insurance is extended for some)	Overall	Overall
**Source (questionnaire, Administrative data, other(specify) or unclear)**	Administrative data and questionnaire (the Current Population Survey, or CPS)	Administrative data	Administrative data
**Notes**	Too high risk of bias	Uses wrong work experience boundary	Too high risk of bias
**Author**	**Wolff**		
**Year**	1997		
**Country**	Hungary		
**Journal**	The William Davidson Institute		
**Type of outcome**	Time‐to‐Event		
**Outcome (there may be more than one, record them all)**	Hazard ratio to job		
**Time Point (s) (record the exact time, there may be more than one, record them all)**	Overall and in one month intervals		
**Source (questionnaire, Administrative data, other(specify) or unclear)**	Administrative data		
**Notes**	Too high risk of bias		

### 14.2 RISK OF BIAS

#### 14.2.1 Risk of bias assessment

**Table:** 

**Author**	**Addison & Portugal**	**Ahn & Ugidos‐Olazabal**	**Amarante, Arim & Dean**
**Year**	2008	1995	2013
**Country**	Portugal	Spain	Uruguay
**Journal**	Economic Letters	Oxford Bulletin of Economics and Statistics	Desarrollo y Sociedad
**Sequence generation**	High	High	High
**Allocation concealment**	High	High	High
**Blinding**	44	44	44
**Incomplete outcome data addressed (Judgement)**	Unclear	Unclear	Unclear
**Incomplete outcome data addressed (Description, quote from paper or describe key information)**	Nothing reported	Nothing reported	Age and date (before/after reform) restricted, otherwise nothing reported
**Free of selective reporting (Judgement)**	1	1	11
**Free of selective reporting (Description, quote from paper or describe key information)**			
**Free of other bias (Judgement)**	3	1	5
**Free of other bias (Description, quote from paper or describe key information)**	In table 1, age‐adjacent pair 44‐45 years old, control group (youngest) and treatment group (oldest) enter with exactly the same number of observations (1,036) and exactly the same transition rate (0.772). The simultaneous occurrence of these events (identical distribution of observations and identical frequency of transitions) is extremely unlikely and suggests an error ‐ unclear whether this (likely) error is present in regressions.		The reform also established a decreasing scheme for benefits instead of a flat and a lot of other changes; see notes. They analyse mean duration but do not take into consideration that the mean depends on the date of inflow as not all spells are complete
**A priori protocol**	No	No	No
**A priori analysis plan**	No	No	No
**Confounding (Judgement)**	5	5	5
**Confounding (Description, quote from paper or describe key information)**	All (except ethnicity) confounders controlled for, imbalances not shown. Restricts to adjacent age groups. Do not consider unobservables in any way or discuss selection. Inspection of Table 1 (showing number of spells and transitions separately for the relevant ages) suggests there may be some selection. Study authors argue similarity with Regression discontinuity design. As described in e.g. Lee & Lemiuex (2010) (Regression Discontinuity Designs in Economics. *Journal of Economic Literature, 48*, 281–355) age‐dependent rules do not lead to as‐good‐as‐random variation since individuals are able to anticipate change in rules. This study does not rule out anticipation effects. Therefore, an important concern remains whether transition rates drop just prior to reaching a cut off age leading to an overestimation of the impact of longer potential durations. Nothing is reported on density around cut‐off. In age‐adjacent pairs where longer potential benefit duration (PBD) is associated with longer non‐employment duration (5 out of 7 pairs), the treatment group (the oldest) is on average 47% larger than the control group (the youngest). In age‐adjacent pairs where longer PBD is NOT associated with longer non‐employment duration (2 out of 7 pairs), the treatment group is on average 20% smaller than the treatment group. This suggests that selection could be driving result. The study relies on a Difference‐in‐Difference approach, yet authors report nothing on the age‐differences in age‐adjacent pairs in control group. Common trends? Only control for elapsed duration.	Do not consider unobservables in any way, estimates the effect of being eligible to benefits or not and do not take into consideration that those eligible for unemployment benefits have different entitlement depending on job tenure (job tenure is controlled for as a continuous variable).	Age and gender considered. The only argument for applying a Regression discontinuity design is a possible age discontinuity before the reform, which is bigger after the reform.
**Method for identifying relevant confounders described by researchers. Yes/No ‐ if Yes describe the method used.**	Some discussion	Very little discussion	No
**Relevant confounders described (See relevant sheet and list confounders and note if they were considered, precise, imbalanced or adjusted)**	All except ethnicity and few more added. Do not show or discuss imbalances but in working paper results with stepwise adding confounders is shown	Do not consider ethnicity, labour market conditions and do not take into consideration that those eligible for unemployment benefits have different entitlement depending on job tenure (job tenure is controlled for as a continuous variable)	Age is the discontinuity. Separate results by gender, otherwise nothing considered.
**Method used for controlling for confounding (At design state)**	Age differences in entitlement, use adjacent age groups	None	Regression discontinuity design
**Method used for controlling for confounding (At analysis stage)**	Complementary log‐log hazard with binomial unobservable. heterogeneity	Cox proportional hazard rate with extensions (estimate the baseline hazard with unequal length of time intervals)	
**Notes**	The maximum duration of benefits is 10 months for those aged less than 25 years and 12 months for those aged between 25 and 29 years. It then rises in 3‐month intervals for each incremental 5 years of age up to a maximum of 30 months at 55 years of age. Use pairs of individuals aged 24 to 25 years, 29 to 30 years, 34 to 35 years, 39 to 40 years, 44 to 45 years, 49 to 50 years, and 54 to 55 years	Construct discrete time periods for unemployment duration. Each month up to the sixth month of unemployment is recorded as a specific time period. From the seventh month we grouped several months into one time period: 7‐9, 10‐12, 13‐15, 16‐21, 22‐33, 34‐45, 46‐57, 58‐69 and 70‐81 months.	The new regulations also attempt to coordinate Unemployment insurance with active labour market policies, and theoretically beneficiaries may lose their Unemployment insurance benefits if they do not participate in training courses offered by the Ministry of Labour. Another change is the introduction of compatibility between the unemployment insurance and holding other economic activity. Also, in the new regime, a worker can interrupt the benefits for a short time, in case he gets a temporary job, and then return to the insurance system. Finally, before February 2009 the benefit could only be claimed within 30 days after the last day of work, in the actual regime there are no restrictions.

**Table:** 

**Author**	**Arntz, Simon & Wilke**	**Arranz, Bulló & Muro**	**Barbanchon**
**Year**	2014	2008	2016
**Country**	Germany	Spain	France
**Journal**	Empirical Economics	Instituto de Estudios Fiscales	Labour Economics
**Sequence generation**	High	High	High
**Allocation concealment**	High	High	High
**Blinding**	44	44	44
**Incomplete outcome data addressed (Judgement)**	22	3	Unclear
**Incomplete outcome data addressed (Description, quote from paper or describe key information)**	Level of missing data (demographics) not mentioned. Two measures of unemployment are created (different approaches to handling gaps in the unemployment spells), censoring rates differ, shown before and after reform. Censoring rates (upper bound) for treated: 13% to 16%; censoring rates for control: 11% to 14%. No censoring for the lower bound unemployment measure by construction	Data discussed at page 14‐18. Missing data not discussed (those with missing data excluded). Censoring level 73‐77.5%	Restricted sample (to avoid confounding) reduces the sample from 32,000 to 17,000. Exclude 50+ of age, jobseekers subject to very specific Unemployment insurance rules and jobseekers in the least and most generous categories. In addition 33% has (by mistake I guess) no employment spell recorded in the register before claiming Unemployment insurance (not sure what they do about that, have they been excluded before the reporting of the above numbers or do they use the employment duration recorded in the unemployment register?) and 20% have inconsistent records (between employment and unemployment registers). Report that those with inconsistent records are excluded. Missing data and censoring not mentioned. Reducing bandwidth to 4, 2, 1 months decreases sample size to 8,352; 3,837 and 1,817
**Free of selective reporting (Judgement)**	11	1	4
**Free of selective reporting (Description, quote from paper or describe key information)**		Sensitivity analysis (p. 31). Unobserved heterogeneity considered (concluded that it shall not be included)	Several sensitivity analyses and results fully reported. However sensitivity analyses not performed for the outcome exit from non‐employment and fuzzy design analysis not performed for the main outcomes (exit from unemployment and non‐employment) but for unemployment and non‐employment duration. No graph of relevant outcome (non‐employment duration)
**Free of other bias (Judgement)**	11	3	5 for bandwidth all and 1 for bandwidth 1,2 and 4 months
**Free of other bias (Description, quote from paper or describe key information)**		Benefit level decreased at the same time	They exclude workers with 6 months employment duration, a typical temporary contract duration (the threshold is 8 months of work). So the bandwidth ‘All’ is two months at one side and four on the other?
**A priori protocol**	No	No	No
**A priori analysis plan**	No	No	No
**Confounding (Judgement)**	33	5	5 for bandwidth 1 and 2 months. 4 for bandwidth 4 months. Not relevant for bandwidth all.
**Confounding (Description, quote from paper or describe key information)**	Only ethnicity not considered, more confounders are added and some discussion of the identifying assumption (of using a DID approach). Although no figure showing trends is provided the discussion is followed by a further restriction on the time periods used for pre and post reform	All, except ethnicity (+ more). The reform also decreased the level of Unemployment insurance benefits. Control group lost their job in 1991 and the treated in 1993, thus cannot separate the effect of change in max duration from change in benefit level. Some imbalance on the remaining confounders too.	All confounders + more considered. Identification strategy, however, subject to risk of selection around cut off. Tests for selection on covariates not convincing. Discontinuity tests around threshold of all confounders and the forcing variable (employment duration) and with different bandwidth. Only for bandwidth less than two months (and excluding those with exactly 6 months employment duration) are the number of significant discontinuities low (2 of 15 on 5% level). However, statistical significance is not the main issue as number of observations goes from approximately 17,000 to 1,800; so rather the magnitude of coefficients is of importance (requires knowledge of covariate means, which are not reported, to judge). Sensitivity analyses alter the results to a large extent for bandwidth 1 and 2 months and to a much lesser extent for bandwidth 4 months.
**Method for identifying relevant confounders described by researchers. Yes/No ‐ if Yes describe the method used.**	Some discussion concerning the restrictions on the sample and the assumption that outcomes of participants and nonparticipants evolve over time in the same way (as a result restrict the pre reform period to 1995‐1996 and post reform period to 1999‐2000 to avoid other legislative changes and reducing bias from anticipation by allowing a gap until implementation, although only 3 months, implementation was April 1997))	Discusses the control variables used	Yes
**Relevant confounders described (See relevant sheet and list confounders and note if they were considered, precise, imbalanced or adjusted)**	Only include male workers with a former full time job in western Germany who have enough foregoing employment to be eligible to maximum entitlement (which again depends on age and was reformed in 1997). Treated are workers 42‐44 years of age and control are workers 36‐41 years of age. Thus there is age imbalance but no other major imbalances. Do not consider ethnicity but more is added. Labour market conditions are only partly considered (by the DID approach) and effects are shown separated by unemployment duration. Descriptives are shown separated by pre and post reform period	All, except ethnicity (+ more). Some imbalances.	Yes and more is added and discontinuity tests of all with different bandwidths.
**Method used for controlling for confounding (At design state)**	Legislative change which is age dependent.	Legislative changes and individual variation due to labour market history. Exclude the year of the reform as inflow increased (decreased) just before (after) the reform	Regression discontinuity design and restricts the sample on age and include only intermediate max entitlement groups
**Method used for controlling for confounding (At analysis stage)**	Difference‐in‐difference on cumulative incidence of distant and local employment	Regression. Discrete‐time hazard (complementary log‐log) with piecewise constant baseline hazard.	Cox proportional hazard rate
**Author**	**Belzil**	**Bover, Arellano & Bentolila**	**Caliendo, Tatsiramos, Uhlendorff**
**Year**	1995	2002	2013
**Country**	Canada	Spain	Germany
**Journal**	The Review of Economics and Statistics	The Economic Journal	Journal of Applied Econometrics
**Sequence generation**	High	High	High
**Allocation concealment**	High	High	High
**Blinding**	44	44	44
**Incomplete outcome data addressed (Judgement)**	Unclear	Unclear	3
**Incomplete outcome data addressed (Description, quote from paper or describe key information)**	Some censoring but level is not reported	Uses survey data, response rate and missing data not reported. Restricts the sample used. In the final sample the censor rate is 54% (on unemployment duration) Maximum entitlement is also a censored variable but the rate of censoring is not reported	Data discussed at page 9‐10 (working paper) and 610 (journal article). The sample in the journal article is the sample B from the working paper. Restricts to those who have worked at least 36 months during the past seven years (so all entitled to max benefit duration given age) and 44‐46 years of age. Missing data not discussed. Censoring level 25‐30% (the journal article says 25‐27%)
**Free of selective reporting (Judgement)**	33	11	1
**Free of selective reporting (Description, quote from paper or describe key information)**	Only the model coefficients of immediate interest (benefit level and duration) is reported		Sensitivity analysis, jointly estimating the unemployment and the employment hazards (p. 15). Among other things unobserved heterogeneity. “Modelling unobserved heterogeneity significantly improves the model fit”, “The effect of extended benefit duration does not differ qualitatively between the models with and without unobserved heterogeneity” (estimates not reported in working paper but in journal article). The effects differ somewhat in magnitude from the univariate model.
**Free of other bias (Judgement)**	Unclear	5	1
**Free of other bias (Description, quote from paper or describe key information)**	Restrict the sample to those who had not dropped out of the labour force by the end of the panel. Nothing further concerning those excluded is givengiven	They are comparing the (benefit covered) spell following the loss of a permanent job with subsequent (non‐covered) spells in between temporary jobs. But: The unemployed are asked each quarter whether they are receiving any unemployment benefits (without distinguishing between Unemployment insurance and Unemployment assistance). The length of benefit entitlement is a censored variable, since it is only observed if both benefits are exhausted before the end of the unemployment spell and the individual still remains in the sample at that time. Benefit duration is equal to one‐third of the accumulated job tenure over some years prior to unemployment (6 years and 4 years, before 1992), with a maximum duration of two years. Unemployment insurance generosity was reduced in 1992 and again in 1993. Entitlement to UA benefits depends on a mix of tenure, age and family characteristics, so estimates of benefit (UA) entitlement duration would be very noisy. UA was made more generous in 1992, but less generous in 1993. There is no information on benefit amounts. No information on elapsed duration or maximum potential duration.	The model in the journal article is a model of unemployment and employment transitions, and is estimated jointly allowing for correlated unobserved heterogeneity. Unclear how to interpret the results for unemployment hazard, so consider the model and results only from working paper in the following.
**A priori protocol**	No	No	No
**A priori analysis plan**	No	No	No
**Confounding (Judgement)**	5	55	2
**Confounding (Description, quote from paper or describe key information)**	Not all confounders considered and no imbalances presented or discussed. Simply state that as the model is designed as a simultaneous recursive system the issue of identification arises naturally and will require exclusion restrictions for some of the exogenous variables (they do exclude, or rather do not include, different variables in the unemployment and re‐employment equations but nothing is discussed). Individual variation in maximum entitlement not fully reported other than it is determined by work experience level and national and regional unemployment rates. There are also individual variations in benefit levels; the effect of this is identified in the same model. A reform (reducing maximum duration) is ignored (only show results comparing mean duration before and after)	They do not have information on maximum benefit entitlement but have to model it and this can only be done for those who exhaust their benefits. Thus maximum entitlement is a censored variable. To obtain a measure of the effect of duration entitlement they assume there are only two hazard rates, depending on whether workers receive benefits or not. Thus, for two workers with benefits at a given time, the one with shorter benefit entitlement has a greater hazard than the worker with longer benefit entitlement by assumption. Based on the assumption that there is an effect they calculate the median unemployment duration for given values of benefit entitlement.	All confounders and more considered. Almost no imbalances and several smoothness and density tests support the design
**Method for identifying relevant confounders described by researchers. Yes/No ‐ if Yes describe the method used.**	No		No, but selectivity is discussed
**Relevant confounders described (See relevant sheet and list confounders and note if they were considered, precise, imbalanced or adjusted)**	Do not consider education and ethnicity.		All (+ more) and almost no imbalances
**Method used for controlling for confounding (At design state)**	None. Maximum entitlement is determined by work experience level and national and regional unemployment rates but nothing further explained or reported except difference between unemployment rates were used before the reform and level of regional rates after		Individual variation in entitlement due to age. Regression discontinuity design
**Method used for controlling for confounding (At analysis stage)**	Linear simultaneous recursive regression of unemployment and re‐employment duration with pre unemployment job tenure and industrial classification excluded from the unemployment equation and unemployment insurance benefit level and maximum duration excluded from the re‐employment equation (pre unemployment weekly earnings are excluded as well)		Proportional hazard rate with piecewise‐constant baseline hazard. Bivariate used in journal article with discrete distribution of unobserved heterogeneity
**Notes**	Also offers a before‐after (BA) (reform of maximum entitlement) analysis where spells which overlap both periods are excluded. They state that the reform reduced the average period of initial entitlement but it may have increased for some individuals. Results reported only as an average BA effect and not separated by increase/decrease.		
**Author**	**Card, Chetty & Weber**	**Chang**	**de Groot & van der Klaauw**
**Year**	2007	2010	2014
**Country**	Austria	USA	Netherlands
**Journal**	The Quarterly Journal of Economics	Dissertation, The Johns Hopkins University	IZA DISCUSSION PAPERS
**Sequence generation**	High	High	High
**Allocation concealment**	High	High	High
**Blinding**	44	44	44
**Incomplete outcome data addressed (Judgement)**	3	Unclear	Unclear
**Incomplete outcome data addressed (Description, quote from paper or describe key information)**	Restrict sample to 20‐50 years of age, exclude voluntary quitters, exclude construction workers, only include those working at their previous firm for strictly between 1 and 5 years (in the past 5 years), final sample 650922 job losses (16% of individuals have more than one job loss). When adding covariates to model they lose 22% of the observations	Censoring rate 6% (from the full data set). Restriction on sample (exclude recalls, not entitled to at least 26 weeks of Unemployment insurance and 55+ aged) and missing data reduces the sample by 64%.	Restrict to individuals entering unemployment between July 2004 and December 2008 who had worked at least 4 of the last 5 years and at least 26 weeks of the last 36 weeks. Exclude workers with an employment history of 10, 11 and 12 years because of additional reform changes for these groups (p. 8). Censor at 3 years but rate not reported. Missing data not reported
**Free of selective reporting (Judgement)**	4	1	1
**Free of selective reporting (Description, quote from paper or describe key information)**	Do not report the results of the analysis restricting the sample to those who are not eligible to both severance payment and extended benefits, only report they obtain similar estimates (p. 1533). The result is reported in the working paper but only in a model with no controls. Show results for other robustness analyses. Authors do not comment on the finding that the coefficient changes from ‐0.093 to ‐0.064 by including basic controls. Report only that the coefficient is stable when including even more controls. Covariates should not change the estimate if the assumption for a valid Regression discontinuity is fulfilled ‐ yet it changes by around 30%. Which estimate would the inclusion of controls for the restricted working paper‐sample give rise to?		
**Free of other bias (Judgement)**	3	1	4
**Free of other bias (Description, quote from paper or describe key information)**	Regression discontinuity design. Those eligible for Unemployment insurance (worked 12 months during the last 2 years) have max duration of 20 weeks with less than 36 months of work during the last 5 years and those with more than 36 months of work have max duration of 30 weeks. ´There is a cut off of 36 months job tenure (same firm within the last 5 years) where the worker receives a severance payment, 20% of the sample is eligible for both severance payment and extended benefits and the coefficient on the restricted sample (excl. these 20%) is ‐0.084 compared to ‐0.093 for the full sample (reported in the working paper, from a model without any controls). To eliminate the double discontinuity problem they include a cubic polynomial in job tenure and a dummy for severance payment eligibility plus interactions. Do not consider bandwidth sensitivity, the bandwidth is two years above/below the threshold.		Max duration changes mostly for those with long employment histories but they do not have exact employment histories. Reform is in 2006 and they have exact employment history since 1999. Before that it is assumed that all have worked since the age of 18.
**A priori protocol**	No	No	No
**A priori analysis plan**	No	No	No
**Confounding (Judgement)**	3	5	5
**Confounding (Description, quote from paper or describe key information)**	Only show figures of lay off frequency and means of confounders around the severance payment cut off; do not show anything concerning the extended benefit cut off, only report that there are no problems and extended benefit status is as good as randomly assigned around the cut off. To eliminate the double discontinuity problem they include a cubic polynomial in job tenure and a dummy for severance payment eligibility plus interactions	Benefits are extended via program participation. The programs are not described other than they are not triggered by economic conditions such as the federal‐state Extended Benefits programs. Endogeneity of extended benefits is taken care of by the timing‐of‐events approach (see fx Abbring, J.H, and van den Berg, G.J. (2003). The non‐parametric identification of treatment effects in duration models, Econometrica, vol. 71, pp. 1491–518). However none of the conditions concerning the data generating process necessary for this approach to identify an effect is discussed or even mentioned (a reference is made to Abbring et al. (2005) “The Effect Of Unemployment Insurance Sanctions On The Transition Rate From Unemployment To Employment,” The Economic Journal, 2005, 115, 602‐630.)	All considered but imbalances not reported. Do not use employment history adjacent groups but one average treatment and one average control group. Assume the effect is linear and report per month effects (reduction range is ‐2 to +22). On page 28 it is stated: ‘Recall that for an entitlement period between ten and 17 months we do not have a proper control group.’ Cannot find where this is mentioned and seems like these entitlement periods are used in the analysis
**Method for identifying relevant confounders described by researchers. Yes/No ‐ if Yes describe the method used.**	Yes, a theoretical model and discussion	No	Some theoretical discussion
**Relevant confounders described (See relevant sheet and list confounders and note if they were considered, precise, imbalanced or adjusted)**	Yes and more is added. Do not show imbalances, only report that there is no significant jump in mean wages or any other covariate around the cut off (p. 1532).	All (and more) except unobservables	All considered but imbalances not reported, only averages of combined treated and control before respectively after the reform (control are those who are not affected by the reform, consists of those with an employment history of 9, 12, 18 and 24 years)).
**Method used for controlling for confounding (At design state)**	Regression discontinuity design. Those eligible for Unemployment insurance (worked 12 months during the last 2 years) have max duration of 20 weeks with less than 36 months of work during the last 5 years and those with more than 36 months of work have max duration of 30 weeks. Restrict sample to 20‐50 years of age, exclude voluntary quitters, exclude construction workers, only include those working at their previous firm for strictly between 1 and 5 years (in the past 5 years),	Not explicitly reported but it is the timing‐of‐events approach	Legislative changes (which varies a lot depending on employment history, from an increase of 2 months to a decrease of 22 months and some unaffected). Difference‐in‐difference
**Method used for controlling for confounding (At analysis stage)**	Cox proportional hazard rate	Mixed proportional hazard rate	Not relevant
**Author**	**Engberg**	**Farber & Valletta**	**Farber, Rothstein & Valletta**
**Year**	1990	2015	2015
**Country**	USA	USA	USA
**Journal**	Dissertation, University of Wisconsin‐Madison	Journal of Human Resources	American Economic Review
**Sequence generation**	High	High	High
**Allocation concealment**	High	High	High
**Blinding**	44	44	44
**Incomplete outcome data addressed (Judgement)**	Unclear	4	Unclear
**Incomplete outcome data addressed (Description, quote from paper or describe key information)**	Only restrictions on those included are reported: Individuals who were recalled to previous job or never found a job and were out of the labour force were eliminated from the sample.	Restrict the set of observations used to the first two of each set of four consecutive CPS (the Current Population Survey, or CPS) rotation groups (so that they have at least two subsequent matched observations). Report that there is a small number of missing values (no numbers are reported). Only includes 20‐64 aged Unemployment insurance eligible. Censoring rate more than 50%	
**Free of selective reporting (Judgement)**	1	11	1
**Free of selective reporting (Description, quote from paper or describe key information)**			
**Free of other bias (Judgement)**	1	5	5
**Free of other bias (Description, quote from paper or describe key information)**		Include 20‐64 aged without considering the impact of including workers planning to retire. The time (duration time) EB is available for some is identical to the time benefits have just exhausted for those where EB is not available. Sensitivity analysis on the sample of non‐eligible, finds a significant effect in the later period. The Unemployment insurance take up rate among eligible is around 50%. Entitlement (to ordinary Unemployment insurance) is set to 6 months regardless of working history (as they do not have access to work history information)	We use the reported duration of unemployment, together with state‐level maximum benefit durations, to assign Unemployment insurance availability to individuals. We assume that job losers are eligible for the full duration of benefits and that each draws benefits continuously from the date of job loss until benefit expiration or exit from unemployment (p 4)
**A priori protocol**	No	No	No
**A priori analysis plan**	No	No	No
**Confounding (Judgement)**	5	5	5
**Confounding (Description, quote from paper or describe key information)**	Interpret the (ordinary) duration dependence coefficient as the change in the HR when benefit exhaustion is approached, have no identification for exhaustion effects. Otherwise include remaining benefits as the value (or quantity) of remaining benefits which is a combination of the (individual) varying levels and (individual) remaining entitlements. Includes both those eligible to the Trade Adjustment Assistance program (TAA) and those not with a dummy to separate, but the assignment to this group is highly correlated with individual characteristics (p. 50 ff) and in addition TAA workers had higher average compensation and longer duration	The effects of extended Unemployment insurance are identified by exploiting differences in Unemployment insurance availability at the individual level (that is what they report, but the individual variation is only due to differences in residence state). Unemployment insurance extensions and rollbacks are triggered by deterioration or improvement in state labour market conditions. TEUC (2002–2004) and EUC (2008–forward) programs, the timing of the extended Unemployment insurance triggers and consequent maximum duration of Unemployment insurance eligibility varied substantially across states and over time. Estimate the effects in the two time periods separate.	Because most new spells of unemployment end before extended benefits could be an important factor, we restrict attention to individuals who have been unemployed for at least 3 full months (p 4).
**Method for identifying relevant confounders described by researchers. Yes/No ‐ if Yes describe the method used.**	Yes, theory and discussion (see in particular p. 22 ff.)	No	Not relevant
**Relevant confounders described (See relevant sheet and list confounders and note if they were considered, precise, imbalanced or adjusted)**	All but labour market conditions. No imbalances are discussed or shown	Yes and one more is added. Do not show imbalances	Not relevant
**Method used for controlling for confounding (At design state)**	None	State differences in extended benefits triggered by deteriorations in labour market conditions	Not relevant
**Method used for controlling for confounding (At analysis stage)**	Proportional hazard	Probit	Not relevant
**Notes**		The specification excludes any potential effects of EB availability on search behaviour for the short‐ term unemployed (include indicator variables for each of the first six months of unemployment and an extension dummy for those who have exhausted benefits and where EB is available). TEUC (2002–2004) and EUC (2008–forward) programs, the timing of the extended Unemployment insurance triggers and consequent maximum duration of Unemployment insurance eligibility varied substantially across states and over time.	
**Author**	**Ferrada**	**Figura & Barnichon**	**Fitzenberger & Wilke**
**Year**	2011	2014	2010
**Country**	Chile	USA	Germany
**Journal**	Dissertation, University of Chicago	Board of Governors of the Federal Reserve System (U.S.). Report.	Oxford Bulletin of Economics & Statistics
**Sequence generation**	High	High	High
**Allocation concealment**	High	High	High
**Blinding**	44	44	44
**Incomplete outcome data addressed (Judgement)**	44	Unclear	22
**Incomplete outcome data addressed (Description, quote from paper or describe key information)**	Uses matched survey and administrative register data. Match rate was 9.4% in 2002 and 64.9% in 2009 (p. 17)	Restrict sample to 20+ permanent job losers, otherwise nothing mentioned (use the Current Population Survey, or CPS, but do not report how the unemployment spells are formed)	Restrict sample to men of age 26‐49 whose unemployment spell starting in 1996‐1997 and begins with receiving unemployment benefits, who have not had a foregoing unemployment spell within a year, whose last employer was located in West Germany. Missing data not mentioned. Two unemployment measures, censoring rates are different 23% and 13% respectively)
**Free of selective reporting (Judgement)**	11	1	11
**Free of selective reporting (Description, quote from paper or describe key information)**			
**Free of other bias (Judgement)**	5	5	11
**Free of other bias (Description, quote from paper or describe key information)**	Individual savings accounts replaced government financed Unemployment insurance in 2002. Compulsory participation of new contracts whereas it was voluntary before 2002 with participation rates not exceeding 4%. The level (post 2002) is decreasing in duration from month one (pre 2002 it decreased after 3 and 6 months and max duration was 12 months). Benefits could be complemented with public transfers (also employer contribution, it is later termed a solidarity fond) for those involuntary separated from an indefinite contract, low savings and having contributed at least 12 months within the last 24 months. For those with indefinite contract max duration is 5 months and apparently varies with number of contributions (see page 23 where it is stated that there was no variation in max duration pre 2002) (with an increase to 7 months (or more?) in 2009) and 1 months for those on temporary contract and levels where lower (for all?) than pre 2002. Conditioning (to the solidarity fond) was reformed in 2009 and temporary contracts can now receive 4 (or more?) months of Unemployment insurance. Severance payments are also available but a bit unclear for how long. Both temporary and indefinite contracts are included although rules are very different and changes (in 2002 and 2009 and also an increase in nominal level in 1996) and max duration is in particular very different. Share with temporary contract is much higher post 2002 than pre. Unsure what the time period analysed is.	Include 20 or more aged without considering the impact of including workers planning to retire. The time (duration time) EB is available for some is identical to the time benefits have just exhausted for those where EB is not available. Sensitivity analysis on the sample of temporary laid off, finds similar effects as for permanent job losers. Do not mention or discuss the Unemployment insurance take up rate among eligible (is around 50% according to for example Farber and Valetta (2015). Entitlement (to ordinary Unemployment insurance) is set to 6 months regardless of working history (as they do not have access to work history information).	
**A priori protocol**	No	No	No
**A priori analysis plan**	No	No	No
**Confounding (Judgement)**	55	5	5
**Confounding (Description, quote from paper or describe key information)**	Uses a very messy reform, see the Other bias item. The identification strategy is not clearly explained, Cox proportional hazard rates are estimated in the old system and in the new system and the author states that when studying both systems together the effects can be estimated by assuming it is the remaining effect that other variables do not account for (p. 23)	All except ethnicity controlled for (+ more). State that the identification strategy is comparing the behaviour of individuals when EEB is available to the behaviour of similar individuals when EEB is unavailable. Unemployment insurance extensions are triggered by deterioration in state labour market conditions which then by construction are imbalanced. However they control for unemployment rate in an individual's labour market segment, defined as four broad occupation groups (production workers, service workers, sales workers or clerks, and professionals). Confounding due to the long time span (1976‐2013) is not considered	“…our analysis does not provide causal estimates but rather explores the association between benefits and unemployment duration as well as post unemployment earnings in the light of non‐stationary search theory.” (page 799)
**Method for identifying relevant confounders described by researchers. Yes/No ‐ if Yes describe the method used.**	None	A little discussion	Not relevant
**Relevant confounders described (See relevant sheet and list confounders and note if they were considered, precise, imbalanced or adjusted)**	All observables except ethnicity are controlled for. No imbalances shown or discussed.	All except ethnicity and more is added. Imbalance on education, gender and industry (and labour market conditions)	Not relevant
**Method used for controlling for confounding (At design state)**	A very messy reform	Restrict sample to job losers (of permanent jobs only). 20+ years of age. Exclude those temporary laid off	Not relevant
**Method used for controlling for confounding (At analysis stage)**	Cox proportional hazard model	Logistic regression	Not relevant
**Author**	**He**	**Hunter**	**Lalive**
**Year**	2013	1990	2007
**Country**	USA	USA	Austria
**Journal**	Dissertation, Clemson University	Dissertation, University of Wisconsin‐Madison	American Economic Review
**Sequence generation**	High	High	High
**Allocation concealment**	High	High	High
**Blinding**	44	44	44
**Incomplete outcome data addressed (Judgement)**	Unclear	Unclear	Unclear
**Incomplete outcome data addressed (Description, quote from paper or describe key information)**	Uses the whole available panel by merging observations from 8 interview months with 8 months gap in between (the Current Population Survey, or CPS). Unemployment insurance eligibility is based on former employment status (even though take up rates are lower than 50% and max duration depends on work history). Do not report attrition and missing data rates.	Nothing (except restrictions on those included from the larger sample) reported	5 percent is censored, otherwise nothing is reported. The relevant effect size for this review is based on the subsample who finds a job (81%)
**Free of selective reporting (Judgement)**	1	1	1
**Free of selective reporting (Description, quote from paper or describe key information)**			
**Free of other bias (Judgement)**	5	4	1
**Free of other bias (Description, quote from paper or describe key information)**	Include individuals who are employed in their first interviewed month (4 consecutive, after 8 months another 4 consecutive months) and also experienced unemployment in at least one of the following seven interviewed periods. Target those who leave employment (either EU or EN), and then measure the probability of these people finding a job. Compute the unemployment duration using the employment status from each interview month. For some (proportion not reported) only the range of unemployment duration can be identified. Unemployment insurance eligibility is based on former employment status (even though take up rates are lower than 50% and max duration depends on work history). Includes 16 to 65 years old without considering retirement issues. Do not consider exhaustion effects for those who do not have access to extensions of benefits	The data relies on retrospective questions, some years back. Potential Unemployment insurance duration is state average, only positive for those (self‐) reporting receiving benefits (those misreporting typically have short spells)	
**A priori protocol**	No	No	No
**A priori analysis plan**	No	No	No
**Confounding (Judgement)**	5	5	4
**Confounding (Description, quote from paper or describe key information)**	Consider all but do not show/mention imbalances. Include individuals up to 65 years of age without considering retirement issues. Do not use (or have access to) work history, so assume everyone who (involuntary) lost job is assumed to be eligible for max duration. Do not consider the low take up rates. Do not have exact information on unemployment durations. Assume: 1) If an individual reported unemployed/employed in both month 4 and month 13, I assume he/she is unemployed/employed during the 8 non‐interview months; 2) If their employment status changed from unemployed in month 4 to employed in month 13, I assume that one finding a job in any month between 5th ~ 13th month are independent events.	Difference in potential eligibility duration (the division into high/low) not commented/explained. Is probably difference between state averages which captures many other causes and individual variation (for example due to work experience and it varies between states (app. A‐2)) is not considered	The relevant outcome is based on the subsample of job seekers who find a job at the end of the unemployment spell; a potentially selective subsample (81% of the total sample) and extension of benefits have an impact on the share leaving for employment, especially in regions with large extensions and especially for women. No other confounders than age, gender and work history (and this confounder only through inclusion restriction) are considered and age‐bandwidth or anything else concerning the Regression discontinuity design is not discussed. Wide band used and only linear trend (potentially different at both sides of the threshold)
**Method for identifying relevant confounders described by researchers. Yes/No ‐ if Yes describe the method used.**	A little discussion concerning marriage and labour market state	Yes, some discussion	No
**Relevant confounders described (See relevant sheet and list confounders and note if they were considered, precise, imbalanced or adjusted)**	Yes and more is added. Do not show or discuss imbalances	All considered and more is added. No imbalances shown or discussed.	Analysis separated by gender, restricts sample on age (age Regression discontinuity design) and those with continuous work history. No other confounders are considered. Wide band used (three years under and above the threshold) and allows for potentially different linear trends in age on both sides of the 50‐year‐old threshold
**Method used for controlling for confounding (At design state)**	None	None	Regression discontinuity design. Only includes non‐steel workers 46 to 53 years old (and 11 months) at the beginning of the unemployment spell and with a continuous work history and only those who finds a job (81%).
**Method used for controlling for confounding (At analysis stage)**	Hazard ratio	Proportional hazard	Duration until exit to job (weeks) is the effect they report based on regression
**Notes**			Job seekers who become unemployed at 50 years of age or older and satisfy a previous work requirement are eligible for 52 weeks of extended benefits rather than 39 weeks. Job seekers who enter unemployment at 50 years of age or older, who had been living in certain regions of Austria for at least six months, and who satisfy a previous work requirement. Those individuals were eligible for 209 weeks of benefits instead of 39 weeks
**Author**	**Lalive & Zweimüller**	**Lalive, Landais & Zweimuller**	**Landais**
**Year**	2004	2015	2015
**Country**	Austria	Austria	USA
**Journal**	Journal of Public Economics	American Economic Review	American Economic Journal: Economic Policy
**Sequence generation**	High	High	High
**Allocation concealment**	High	High	High
**Blinding**	44	44	44
**Incomplete outcome data addressed (Judgement)**	1	Unclear	Unclear
**Incomplete outcome data addressed (Description, quote from paper or describe key information)**	Restrict sample to 45‐54 years of age, men only, only those with a continuous work history since 1972. Missing data is not mentioned. 1.9% censoring	Restrictions on sample: exclude all workers employed or reemployed in the steel sector. Mainly focus on unemployed men aged 50 to 54 because they cannot go directly from unemployment to early retirement (in AER paper: As a consequence, in our baseline sample, we focus attention to workers with age between 46 and 54 at the start of a spell). Exclude from the analysis the set of treated regions that were excluded after the 1991‐reform (change to original 1988 reform). Missing data not reported and apparently there is no censoring (spells start 1988‐1997 and can be followed to 2010), however, exclude those who did not find a job from the relevant estimate (for this review)	
**Free of selective reporting (Judgement)**	1	1	4
**Free of selective reporting (Description, quote from paper or describe key information)**			In the estimation, they also considered models where they include covariates as additional regressors, results not shown. Why only use Louisiana and not all 5 states (they have an explanation for Georgia in the appendix but not for the rest)
**Free of other bias (Judgement)**	1	3	Unclear
**Free of other bias (Description, quote from paper or describe key information)**		In a robustness analysis, they address the two main potential confounders for the results. First, they provide evidence that the results are unlikely to be driven by region‐specific shocks contemporaneous with the REBP program. Second, they show that the results are unlikely to be confounded by selection, i.e. a change in unobserved characteristics of non‐eligible workers contemporaneous with the REBP program. It is unclear what the counterfactual is as included workers are not restricted on work history, so unclear if they are entitled to less than 30, 39 or 52 weeks (depending on age and period)	The kink is exactly at the exhaustion point. They can only follow the spells till the exhaustion point. They estimate an average effect on the hazard which requires that the heterogeneity effect is additively separable, meaning that the unobserved heterogeneity only acts as a shifter, independently of max duration
**A priori protocol**	No	No	No
**A priori analysis plan**	No	No	No
**Confounding (Judgement)**	2	4	5
**Confounding (Description, quote from paper or describe key information)**	All + more confounders considered and identifying assumptions discussed. Imbalances not shown or discussed on all confounders	All confounders considered and controlled for and potential remaining confounding considered and discussed. However, the relevant outcome is based on the subsample of job seekers who find a job at the end of the unemployment spell, a potentially selective subsample (approximately 84% of the sample used for unemployment duration) and extension of benefits have an impact on the share leaving for employment, according to Lalive, 2007. In addition, include workers whose work history is not sufficient to maximum entitlement, thus the control entitlement is not either 30, 39 or 52 weeks, but unclear	Rests on untestable strong assumptions about differentiability and unobserved heterogeneity: the estimates are the effect of a marginal increase in max duration for the max duration and the assignment variable (previous earnings) fixed at their kink point value (which in the case of max duration is the exhaustion point) integrated on the distribution of the unobservable. This can be thought of as an average treatment effect (ATE) weighted by the ex‐ante probability of being at the kink given heterogeneity type. Requires strong assumptions: the direct marginal effect of the assignment variable on the outcome should be smooth; the derivative of the conditional probability density function (of the outcome) is continuous for all epsilon (epsilon is unobservable heterogeneity) at the kink so that density of the unobserved heterogeneity evolves smoothly with the assignment variable at the kink. The two conditions are needed because a marginal increase in the assignment variable induces an effect on the outcome through max duration (because of the deterministic relationship between max duration and the assignment variable) but also through the direct effect of the assignment variable on the outcome and through the change in the distribution of the unobserved heterogeneity. Only if the latter two effects are smooth and cancel out by differencing on both sides of the kink can the change in the derivative of the conditional expectation function at the kink isolate the causal effect of max duration on the outcome.
**Method for identifying relevant confounders described by researchers. Yes/No ‐ if Yes describe the method used.**	Yes, identifying assumptions discussed	Yes. Discussion of potential confounding and selection, however not based on the subsample of those finding a job that is relevant for this review. The reform had an impact on the share leaving for employment (see Lalive, 2007)	State that: covariates are generally not needed for consistency in estimating the average (unconditional) treatment effect
**Relevant confounders described (See relevant sheet and list confounders and note if they were considered, precise, imbalanced or adjusted)**	Yes and more is added. Considers imbalances in unemployment outcomes across treated (age and control regions), (and between steel workers and non‐steel workers leading to the second strategy, between a subgroup of treated regions and control regions leading to the third strategy and between part of the treated regions and the rest of the treated regions leading to the fourth strategy). Otherwise do not show imbalances	Yes and more is added. No important imbalances on the total sample but the subsample of those finding a job (which is the basis for the relevant estimate for this review) is not shown	Only: to assess the validity of the smooth density assumption, it is useful to check whether pre‐determined covariates have a c.d.f that is twice continuously differentiable with respect to the assignment variable. Estimating changes in the slope of the conditional expectation function of some pre‐determined covariates like age, education or gender given the assignment variable
**Method used for controlling for confounding (At design state)**	Legislative changes (50+ workers living in regions with a large steel sector) and difference‐in‐difference‐in‐difference (age‐specific and other time trends) and analysing further 3 different subgroups (non‐steel workers, specific treated regions with favourable labour market conditions and part of the treated regions serving as controls due to later changes to the reform).	Legislative changes and difference‐in‐difference. Unemployment insurance extensions was enacted only in a subset of regions and for a subset of workers (depending on work history and age)	Regression kink
**Method used for controlling for confounding (At analysis stage)**	Cox proportional hazard rate	Regression	Not relevant
**Notes**	Maximum duration for treated (50+ and living in specific regions) is 209 weeks. In non‐treated regions max duration is 39 weeks for 40‐49 year old workers and 52 weeks for 50+ year old workers	Maximum duration for treated (50+ and living in specific regions) is 209 weeks. In non‐treated regions max duration is 39 weeks for 40‐49 year old workers and 52 weeks for 50+ year old workers	
**Author**	**Lee & Wilke**	**Lubyova & van Ours**	**Machikita, Kohara & Sasaki**
**Year**	2009	1997	2013
**Country**	Germany	Slovakia	Japan
**Journal**	Journal of business and economic statistic	European Economic Review	IZA DISCUSSION PAPERS
**Sequence generation**	High	High	High
**Allocation concealment**	High	High	High
**Blinding**	44	44	44
**Incomplete outcome data addressed (Judgement)**	22	Unclear	Unclear
**Incomplete outcome data addressed (Description, quote from paper or describe key information)**	Missing data not mentioned. Two measures of unemployment, censoring rates differ. Censoring rates for treated: 16% and 23%; censoring rates for control: 11% and 19%	There is censoring (of those exhausting their benefits) but the level is not reported. Nothing further (other missing data) is reported	Exclude the following types of individuals among all those eligible for Unemployment insurance except: (1) Individuals who are over 65 years old, (2) Seasonal workers, (3) Day workers, and (4) Unemployed job‐seekers who remain unemployed for more than 365 days. Otherwise nothing is reported.
**Free of selective reporting (Judgement)**	11	1	11
**Free of selective reporting (Description, quote from paper or describe key information)**			
**Free of other bias (Judgement)**	22	1	55
**Free of other bias (Description, quote from paper or describe key information)**	Report results for different combinations of pre and post reform years and results are somewhat different for low wage workers		Exclude unemployed job‐seekers who remain unemployed for more than 365 days. Cannot observe the exits from unemployment if job‐seekers get a position with a firm that is not involved in the Japanese Unemployment insurance system
**A priori protocol**	No	No	No
**A priori analysis plan**	No	No	No
**Confounding (Judgement)**	44	5	5
**Confounding (Description, quote from paper or describe key information)**	Not all confounders considered and no discussion of the identifying assumption (of using a DID approach)	Legislative changes in January (1992 an increase and 1995 a decrease in max duration) and uses inflow data from first quarter in the two subsequent years (1991/1992 and 1994/1995); i.e. immediately after the change and almost a year before the change. Do not consider the possibility that unemployment may have been postponed/delayed due to the changes. Compares hazard rates for two subsequent years separated into two‐monthly duration intervals and in addition include an inflow year dummy; this is too many year dummies. There are two analyses, 1991/1992 where max duration was shortened and 1994/1995 where max duration was raised; nevertheless the sign of the year dummy is positive in both analyses and the majority of duration interval coefficients (measuring change in max duration effects) are negative in both analyses. Do not discuss imbalances in confounders and do not discuss unobservables. Strange division of several confounders	They apply a difference‐in‐difference approach. Max duration jumps at age 45 for involuntary unemployed and do not jump with age for voluntary unemployed. It also depends on experience, but they estimate effects separated by threshold experience groups. They implicitly assume that the age trend for involuntary and voluntary unemployed is the same in absence of an age discontinuity for the involuntary unemployed. However they do not seem to realise that and further the validity of the assumption is questionable. Besides, only for experience group 1‐4 years of employment is max duration the same for workers involuntary and voluntary unemployed below 45 years of age, for the remaining two experience groups there is also a difference between max duration for workers below 45 years of age depending on being involuntary or voluntary unemployed.
**Method for identifying relevant confounders described by researchers. Yes/No ‐ if Yes describe the method used.**	Some discussion concerning the restrictions on the sample but do not discuss the assumption that outcomes of participants and nonparticipants evolve over time in the same way or provide a figure.	No	Not relevant
**Relevant confounders described (See relevant sheet and list confounders and note if they were considered, precise, imbalanced or adjusted)**	Only include male workers (not from the agriculture sector and no recalls to former employer) who have enough foregoing employment to be eligible to maximum entitlement (which again depends on age and was reformed in 1984). Treated are workers 44‐48 years of age and control are workers 36‐41 years of age. Thus there is age imbalance and other than that only the share of high wage workers are shown (no imbalances). Do not consider education and ethnicity. Labour market conditions are only partly considered (by the DID approach) and effects are shown separated by unemployment duration. Descriptives are shown separated by pre and post reform period and only shown for married men as this is the population used for most of the analyses.	Yes and more is added. Do not show imbalances. There may be a problem with collinarity. Compares hazard rates for two subsequent years separated into two‐monthly duration intervals and in addition include an inflow year dummy; this is too many year dummies.	Not relevant
**Method used for controlling for confounding (At design state)**	Legislative change which is age dependent.	Legislative changes	Exploits the sharp discontinuities with respect to three types of individual characteristic: the reason for displacement from the previous job, age at displacement, and job tenure at the previous job.
**Method used for controlling for confounding (At analysis stage)**	Difference‐in‐difference on survival probability	Proportional hazard rate with piecewise‐constant baseline hazard.	Not relevant
**Notes**	Do not provide useable effect estimate	January 1992. The eligibility period was halved from 12 to 6 months and the replacement ratios were slightly reduced. January 1995: the eligibility period was prolonged up to 9 months according to the age of unemployed. For younger than 30 years the entitlement period did not change, for workers aged between 30 and 45 the entitlement period was lengthened to 8 months, for workers older than 45 years it was lengthened to 9 months.	Select these 4 groups (among others, see table 1): the first group who experienced displacement due to exogenous reasons at the age of 45 (180–270 days in this sample); the second group who experienced displacement due to exogenous reasons at the age of 44 (90–240 days in this sample); the third group who left their previous job voluntarily at the age of 45 (90–120 days in this sample); the fourth group who left their previous job voluntarily at the age of 44 (90–120 days in this sample).
**Author**	**Micklewright & Nagy**	**Nekoei & Weber**	**Newton & Rosen**
**Year**	1995	2015	1979
**Country**	Hungary	Austria	USA
**Journal**	Unemployment insurance working papers in economics	IZA DISCUSSION PAPERS	Southern Economic Journal
**Sequence generation**	High	High	High
**Allocation concealment**	High	High	High
**Blinding**	44	44	44
**Incomplete outcome data addressed (Judgement)**	11	Unclear	2
**Incomplete outcome data addressed (Description, quote from paper or describe key information)**	Restrict the sample to new spells starting in the month before or after the reform. Quitters and those receiving statutory severance payment prior to UI are not eligible. Censoring rate 4%.	Those above 40 years of age are eligible for an extension of 9 weeks (from 30 to 39) if they have been employed for 6 years during the last 10 years and are laid off (under 40 and worked for 3 of the last 5 years are eligible for 30 weeks). They restrict the sample to those eligible (to 39 weeks if they were above 40) aged 30‐50. Otherwise nothing reported	Exclude 5% (receiving Unemployment insurance from more than one state, invalid claim and spell continued beyond the date when data was gathered)
**Free of selective reporting (Judgement)**	1	4	1
**Free of selective reporting (Description, quote from paper or describe key information)**		They refer to an online Appendix, where several robustness checks are presented, including different bandwidths. However, the online appendix available does not contain the tables referred to in the paper. Found the relevant information in the Dissertation of Nekoei (Harvard University, 2014)	
**Free of other bias (Judgement)**	1	5 for results with 10 years bandwidth and 4 for the rest	2
**Free of other bias (Description, quote from paper or describe key information)**		Ignore the other major reform in 1988: Maximum duration for those aged 50 or above and living in specific regions is extended to 209 weeks. The results shown are for very wide bandwidths (10 years) including those aged 50. Non‐employment calculations exclude those unemployed for more than 2 years. Not reported what the censoring level (if any) is for the hazard rates, nor the method used for estimating HRs. Finding a job within 30 weeks includes the exhaustion effect for the non‐treated and finding job within 39 weeks includes exhaustion effect of treated as well.	Exclude those whose spell continues beyond the date when the data was gathered (1%)
**A priori protocol**	No	No	No
**A priori analysis plan**	No	No	No
**Confounding (Judgement)**	4	4	5
**Confounding (Description, quote from paper or describe key information)**	Some imbalances and ethnicity not considered. Figures of hazard rates before and after the reform divided by gender and work history. Do not control for any other observable confounders	Use pre reform data to show there is no discontinuity before the reform. Cannot see why this is useful, there is no reason to strategically timing the layoff date before the reform so showing that there is no discontinuity just confirms that? It is not helpful in judging if there is strategic timing after the reform. Only shows that there is not something else going on around 40 years of age. Possible discontinuity wrt tenure and experience around cut off. Show smoothness results for all predicted outcomes around cut‐off and for different bandwidths (‘problem’ with the wage outcomes)	Only control for gender, race and average unemployment rate. Include opportunity cost of unemployment and an interaction term of opportunity cost and maximum duration. Do not discuss exogeneity/endogeneity of the maximum duration, only state that maximum duration depends upon the weekly benefit amount and total earnings in the base year. Change in the schedule relating maximum weekly benefit amount to high quarter earnings (unclear what that means) and the authors suggest this variation will help isolating the impact of increases in the opportunity cost of unemployment on duration of unemployment (p. 777)
**Method for identifying relevant confounders described by researchers. Yes/No ‐ if Yes describe the method used.**	Discuss if there is a difference in how quickly job losers claim benefits before and after the reform and if inflows were different. Job losers claimed benefits a little faster before than after the reform. Concerning inflows the evidence is inconclusive and it is noted that there was uncertainty surrounding the date of introduction of the new benefit scheme.	No	No
**Relevant confounders described (See relevant sheet and list confounders and note if they were considered, precise, imbalanced or adjusted)**	All, except ethnicity considered and more is added. Some imbalance on geographical location (indirectly a measure of labour market state) and level of benefits (which was also a part of the reform), Small imbalance on work history. Otherwise no imbalances.	Control for gender, marital status, ethnicity, education, tenure, experience, month and week of layoff, industry, firm characteristics. Selection on observables (discontinuity regression) shown for all, except education, month and week of layoff, industry and firm characteristics. Potential discontinuity wrt tenure and experience	Only gender and race (white/black) and average state‐wide unemployment rate which does not vary much over the period. No imbalances are shown or discussed
**Method used for controlling for confounding (At design state)**	Legislative changes. Regression discontinuity design. Existing UI claims are ‘grandfathered’ and they compare exit rates of claims starting in the month before and the month after the legislative change	Regression discontinuity design. Age threshold. After 1989 those above 40 years of age are eligible for an extension of 9 weeks (from 30 to 39) if they have been employed for 6 years during the last 10 years and are laid off. Use only age and not date of reform.	None
**Method used for controlling for confounding (At analysis stage)**	Figures of hazard rates before and after the reform	Hazard rates are estimated but no information given.	Maximum likelihood regression (assuming normality)
**Notes**	Do not provide useable effect estimate		
**Author**	**Puhani**	**Rebollo‐Sanz & García‐Pérez**	**Rothstein**
**Year**	2000	2015	2011
**Country**	Poland	Spain	USA
**Journal**	Journal of Population Economics	Studies on the Spanish Economy	NBER Working Papers
**Sequence generation**	High	High	High
**Allocation concealment**	High	High	High
**Blinding**	44	44	44
**Incomplete outcome data addressed (Judgement)**	1	33	5
**Incomplete outcome data addressed (Description, quote from paper or describe key information)**		The authors right‐censor any observed spells of unemployment longer than or equal to 36 months and any observed spell of employment longer than or equal to 120 months.. Censor rate is 6.81 for unemployment and 22.93 for employment.	Include all job losers and assume all are entitled to max duration. Mobility and non‐response leads to attrition of 10% of the unemployed each month (p 17 in NBER). Restrict the set of observations used to the first three of each set of four consecutive CPS (the Current Population Survey, or CPS) rotation groups (so that they have at least three subsequent matched observations) and treat and individual observed for three periods as two distinct observations. No other information given on for example age restrictions. Censoring rate approximately 60%
**Free of selective reporting (Judgement)**	1	11	11
**Free of selective reporting (Description, quote from paper or describe key information)**			
**Free of other bias (Judgement)**	3	11	5
**Free of other bias (Description, quote from paper or describe key information)**	The data relies on retrospective questions, some years back.		Include 20 and more aged without considering the impact of including workers planning to retire. The time (duration time) extensions are available for some is identical to the time benefits have just exhausted for those where extensions are not available. Assume none of the extensions are anticipated by newly unemployed, implying they cannot have an impact from the start of the spell. Do not mention or discuss the Unemployment insurance take up rate among eligible (is around 50% according to for example Farber and Valetta (2015). Entitlement (to ordinary Unemployment insurance) is set to 6 months regardless of working history (as they do not have access to work history information).
**A priori protocol**	No	No	No
**A priori analysis plan**	No	No	No
**Confounding (Judgement)**	5	5	5
**Confounding (Description, quote from paper or describe key information)**	Uses a legislative change in the Unemployment insurance system and employ a Difference‐in‐difference model with non‐Unemployment insurance receivers as control. Apparently school leavers (who have a 3 month waiting period) and those qualified for Unemployment benefit until retirement are included in the model but unclear how they are handled and some guessing is involved concerning who are school leavers and qualified to Unemployment benefit until retirement). No discussion of the identifying assumption (that outcomes of participants and nonparticipants evolve over time in the same way)		Three identification strategies (and one for exhaustion effects). Unemployment insurance extensions are triggered by deterioration in state labour market conditions which then by construction are imbalanced. However they control for labour demand conditions and in one strategy use non‐eligible (defined as quitters) as control. However there may be non‐eligible among the treated too (as they assume all involuntary job losers are entitled to full duration of Unemployment insurance and also take up Unemployment insurance)
**Method for identifying relevant confounders described by researchers. Yes/No ‐ if Yes describe the method used.**	No	Not relevant	Not relevant
**Relevant confounders described (See relevant sheet and list confounders and note if they were considered, precise, imbalanced or adjusted)**	All except ethnicity and more is added. No imbalances are shown or discussed (although separate analyses on gender) and apparently school leavers and those qualified for Unemployment benefit until retirement are included but unclear how they are handled	Not relevant	Not relevant
**Method used for controlling for confounding (At design state)**	None	Entitlement depends on reason for unemployment and duration of employment and the way they identify the incentive effect of entitlement length is: They refer to the “timing‐of‐events approach” as ‘This approach is able to solve the endogeneity problem caused by selective treatment by exploiting the variation in the timing of each transition’ (p. 12) (however this approach, among other things, assumes the timing is random and not foreseen…….!) Further they state (p. 12): Handling the selection problems outlined above requires the control of observable and unobservable individual differences and an allowance for the correlation between different unemployment spells and employment processes. This is accomplished by the simultaneous estimation of employment and unemployment spells with correlated unobservable characteristics. The non‐random selection process due to observed characteristics is controlled by the inclusion of covariates that comprise current and past individual and job characteristics. To account for selectivity in the level of unobserved characteristics, we specify unobserved heterogeneity specific to each transition, thereby allowing for correlation between the different states of the individual's history due to his time‐invariant unobserved characteristics. By combining such a design with our precise data, the effect of interest can be separated from selectivity issues.’ To further convince the reader they add as arguments: Additional sources of identification include the existence of repeated employment and unemployment spells …… and, more importantly, the abundance of exogenous time‐varying covariates' (p. 13)	Not relevant
**Method used for controlling for confounding (At analysis stage)**	Logit type’ hazard	Not relevant	Not relevant
**Author**	**Schmieder, von Wachter & Bender**	**Schmieder, von Wachter & Bender**	**U.S. Department of Labor**
**Year**	2012	2016	1995
**Country**	Germany	Germany	USA
**Journal**	Quarterly Journal of Economics	American Economic Review	Unemployment Insurance Occasional Paper
**Sequence generation**	High	High	High
**Allocation concealment**	High	High	High
**Blinding**	44	44	44
**Incomplete outcome data addressed (Judgement)**	Unclear	Unclear	2
**Incomplete outcome data addressed (Description, quote from paper or describe key information)**	Data discussed at page 7‐8, descriptive statistics in table 2. Missing data not mentioned. Censoring level not mentioned but taken care of by this strategy: The duration of non‐employment is measured as the time between the start of receiving Unemployment insurance benefits and the date of the next registered employment spell. Since some people take many years before returning to registered employment and others never do so, we cap non‐employment durations at 36 months and set the duration of all longer spells at this cap.. Only fresh spells with max. entitlement for their age group (the 2009 paper)	Data discussed at page 745‐746, no descriptive statistics, they refer to Schmieder et al., 2012. Missing data level not mentioned. Censoring level not mentioned. Only fresh spells (of those aged 40‐46) with max. entitlement for their age group who involuntary quit (employed at least 36 months (44 months for the 44 age cut off) the last 7 years. OBS this is different than in the 2012 paper where the level is set to 52 months the last 7 years and age restriction is 40‐49, although they analyse the 3 age cut offs separately but do not pool any age cut offs as in the current version (pool 42 and 44 cut off and do not show 44 separately).	Missing data: 8% for males and 7% for females. Total censoring 13% (13.28 for men and 14.07 for women)
**Free of selective reporting (Judgement)**	4	4	1
**Free of selective reporting (Description, quote from paper or describe key information)**	Furthermore we estimated our main specifications controlling for observables, and again obtained virtually the same coefficients (page 25 in the 2010 paper and 720 in 2012 article). Do not show the results and do not report which observables.	Refer to results concerning smoothness of predetermined variables around the 44 age threshold but results not shown and the main analysis do not include the 44 age threshold separately; only pooled with the 42 age threshold	
**Free of other bias (Judgement)**	5 for age cut offs 44 and 49. Cut off 42: 4	Unclear and 5 for the pooled analysis	1
**Free of other bias (Description, quote from paper or describe key information)**	Report results for different bandwidths (2 years, 1 year, 0.5 year and 0.2 year); results vary somewhat, with 30% for age cut off 42 and especially for age cut offs 44 and 49 where the effect almost doubles from widest to the narrowest bandwidth.	The bandwidth is reported to be 2 years but magnitude of coefficients are much larger than the results reported in the 2012 paper, especially the pooled analysis where the separate coefficients from the 2012 paper are 0.78 and 0.41 and the pooled analysis of the current paper produces a coefficient of 0.72 (and 42 age cut off coefficient of 0.95 compared to the 2012 result of 0.78) In the online app. bandwidth 1 year and 0.5 year for the pooled analysis (only) is shown; magnitude varies by 64% from widest to narrowest bandwidth and excluding observation within one month of the threshold increases the variation to 91%	
**A priori protocol**	No	No	No
**A priori analysis plan**	No	No	No
**Confounding (Judgement)**	4	4	5
**Confounding (Description, quote from paper or describe key information)**	The authors test the Regression discontinuity assumption by testing whether observable characteristics (Education, gender, foreign citizen, pre wage, tenure in last job, tenure in occupation, tenure in industry and experience) vary continuously at the points of discontinuity. Further they look at the smoothness of the density around the cut‐offs and discuss the results. They estimated the main specifications controlling for observables, and state they obtained virtually the same coefficients (page 25 in the 2010 paper and 720 in 2012 article). Do not show the results and do not report which observables.	The authors test the Regression discontinuity assumption by testing whether observable characteristics (Education, gender, foreign citizen, pre wage, tenure in last job, UR (not reported what that is) at start of unempl. county UR (not reported what that is) at start of unempl. and experience) vary continuously at the points of discontinuity. Do not consider tenure in occupation and tenure in industry as in the 2012 paper. Further they look at the smoothness of the density around the cut‐offs but refer to the 2012 paper for discussion (in the 2012 paper the restrictions on included participants are different and they do not pool the two age thresholds). They estimate the main specifications controlling for observables. Do not show the results and do not report which observables. Sensitivity analyses only reported for the pooled analysis	Do not explain what cause different max entitlement, nor show the max and min; only the mean is reported
**Method for identifying relevant confounders described by researchers. Yes/No ‐ if Yes describe the method used.**	No	No	No
**Relevant confounders described (See relevant sheet and list confounders and note if they were considered, precise, imbalanced or adjusted)**	Age and unobservables. “It turns out that for most of the outcomes we consider, in particular unemployment and non‐employment durations, other variables in our dataset have little explanatory power (partly because we estimate our model on a relatively homogenous sample of workers) The efficiency gain from this is very small, so that we prefer to present the raw estimates without controlling for additional variables.” (page 9 in the 2009 paper). Furthermore we estimated our main specifications controlling for observables, and again obtained virtually the same coefficients (page 25 in the 2010 paper and 720 in 2012 article). Do not show the results and do not report which observables.	Age and unobservables. While these findings point to a minor violation of the Regression discontinuity identification assumptions, these should have a relatively small impact on the overall results. In fact, neither trimming observations close to the eligibility thresholds nor directly controlling for observable characteristics affects our results. To ensure that our results are not affected by sorting around the threshold and by particular implementation choices of the Regression discontinuity estimator, we performed multiple robustness checks that are summarized in the online Appendix” (page 747). Do not show the results with observables and do not report which observables. Sensitivity analyses only reported for the pooled analysis.	All except education and unobservables is considered. No imbalances shown or discussed. What causes the different max entitlements is not reported (could be strictly Extended benefits or work history dependent)
**Method used for controlling for confounding (At design state)**	Individual variation in entitlement due to age	Individual variation in entitlement due to age	None
**Method used for controlling for confounding (At analysis stage)**	Regression	Regression	Cox proportional hazard rate
**Notes**			Four data sets: 1) Continuous Wage and Benefit History (CWBH) administrative; 2)Job Search Assistance Research Project (JSARP) survey, 1979‐1981; 3) Federal Supplemental Benefit (FSB) follow‐up, survey, 1975‐1978; 4) Newton‐Rosen, admin, 1974‐1975. Only 2) is relevant for this review
**Author**	**Valletta**	**Van Ours & Vodopivec**	**Van Ours & Vodopivec2**
**Year**	2014	2008	2006
**Country**	USA	Slovenia	Slovenia
**Journal**	Federal Reserve Bank of San Francisco, Working Paper Series	Journal of Public Economics	Journal of Labor Economics
**Sequence generation**	High	High	High
**Allocation concealment**	High	High	High
**Blinding**	44	44	44
**Incomplete outcome data addressed (Judgement)**	5	Unclear (4 for wage)	3
**Incomplete outcome data addressed (Description, quote from paper or describe key information)**	Restrict the set of observations used to the first two of each set of four consecutive CPS (the Current Population Survey, or CPS) rotation groups (so that they have at least two subsequent matched observations). Report that there is a small number of missing values (no numbers are reported). Only includes Unemployment insurance eligible but of all ages (16‐ (more than 65)). Censoring rate more than 50%	17701 found post‐unemployment jobs, have complete wage data for 8393 (missing data level 53%) and nothing further is discussed	Level of incomplete data not reported (p.358). Censoring level not reported.
**Free of selective reporting (Judgement)**	11	1	2
**Free of selective reporting (Description, quote from paper or describe key information)**		Sensitivity analysis and their results for the wage equation are shown. Both results for overall effect on probability and HR and divided on magnitude of change are shown	Sensitivity analysis (p. 374). Among other things unobserved heterogeneity. “Hardly affect the other parameter estimates” (estimates available on request)
**Free of other bias (Judgement)**	5	1	1
**Free of other bias (Description, quote from paper or describe key information)**	Include 16‐ aged without considering the impact of including workers planning to retire (and the very young who probably do not have 26 weeks of Unemployment insurance). The time (duration time) extensions are available for some is identical to the time benefits have just exhausted for those where extension are not available. Sensitivity analysis on the sample of non‐eligible, finds a significant effect in the later period. The Unemployment insurance take up rate among eligible is around 50%. Entitlement (to ordinary Unemployment insurance) is set to 6 months regardless of working history (as they do not have access to work history information)		
**A priori protocol**	No	No	No
**A priori analysis plan**	No	No	No
**Confounding (Judgement)**	4	5 for work experience more than 5 years. 4 for work experience 2.5‐5 years	5
**Confounding (Description, quote from paper or describe key information)**	The effects of extended Unemployment insurance are identified by exploiting differences in Unemployment insurance availability at the state level. Unemployment insurance extensions and rollbacks are triggered by deterioration or improvement in state labour market conditions. TEUC (2002–2004) and EUC (2008–forward) programs, the timing of the extended Unemployment insurance triggers and consequent maximum duration of Unemployment insurance eligibility varied substantially across states and over time. Estimate the effects in the two time periods separate.	The treated differs on work experience, varies between 2.5 years to 20 years (2.5‐5; 5‐10; 10‐15 and 15‐20). The control groups work experience is between 1 year and 2.5 years. The reform also had several other measures aimed at speeding up reemployment (see p. 686)	All confounders except ethnicity are considered (+ more). No imbalances shown or discussed except work experience. The reform also had several other measures aimed at speeding up reemployment (see p. 355). NOTE in this paper an incorrect boundary for no change is used.
**Method for identifying relevant confounders described by researchers. Yes/No ‐ if Yes describe the method used.**	No	Yes	No
**Relevant confounders described (See relevant sheet and list confounders and note if they were considered, precise, imbalanced or adjusted)**	Yes and one more is added. Do not show imbalances	All except ethnicity and unemployment duration, more is added. The treated differs on work experience, varies between 2.5 years to 20 years and is divided into sub groups dependent on their maximum entitlement (2.5‐5; 5‐10; 10‐15 and 15‐20 years). The control groups work experience is between 1 year and 2.5 years. The group of workers with 1‐2.5 years of work experience is probably insufficiently comparable to the group of workers with more than 5 years of work experience.	All, except ethnicity (+ more). No imbalances shown but there is an imbalance (on work experience and age) due to work experience requirements
**Method used for controlling for confounding (At design state)**	State differences in extended benefits triggered by deteriorations in labour market conditions	Difference‐in‐difference, legislative changes, reform in October 1998, uses data of periods of one year, starting 2‐3 month before and after the reform was introduced. Individual variation in work history.	Legislative changes and individual variation in entitlement due to labour market history
**Method used for controlling for confounding (At analysis stage)**	Logit	Proportional hazard rate with piecewise‐constant baseline hazard.	Difference‐in‐difference Regression. Proportional hazard rate with piecewise‐constant baseline hazard
**Notes**	The specification is also split on 26 weeks of unemployment and not surprisingly only shows effects of EB availability for those who have exhausted ordinary (incl. former extension) benefits. TEUC (2002–2004) and EUC (2008–forward) programs, the timing of the extended Unemployment insurance triggers and consequent maximum duration of Unemployment insurance eligibility varied substantially across states and over time.

**Table:** 

**Author**	**Winter‐Ember**	**Wolff**
**Year**	1998	1997
**Country**	Austria	Hungary
**Journal**	Oxford Bulletin of Economics and Statistics	The William Davidson Institute
**Sequence generation**	High	High
**Allocation concealment**	High	High
**Blinding**	44	44
**Incomplete outcome data addressed (Judgement)**	22	2
**Incomplete outcome data addressed (Description, quote from paper or describe key information)**	17% (22%) of spells for men (women) are censored. Otherwise nothing reported	Restrict sample to inflows in December 1992 and January 1993. On recall is excluded and men 55+ years of age and women 50+ are excluded. Censoring rate is 39% for men and 48.4% for women. Missing data not mentioned
**Free of selective reporting (Judgement)**	11	1
**Free of selective reporting (Description, quote from paper or describe key information)**		Show results of several specifications
**Free of other bias (Judgement)**	44	3
**Free of other bias (Description, quote from paper or describe key information)**	Only observe work experience from 1972 (should be 1961 for complete data) ‐ assume work requirement fulfilled if fulfilled in the observed period. Not specified what happens to those above age 50 (or below) not fulfilling work requirement. Uncertainty regarding eligibility.	Benefit level decreased at the same time
**A priori protocol**	No	No
**A priori analysis plan**	No	No
**Confounding (Judgement)**	5	5
**Confounding (Description, quote from paper or describe key information)**	‘Extension has led to sharp increase in unemployment entry in concerned countries (p.35). Policy motivated by problems regarding privatization of nationalized firms. Counties selected on basis of labour market conditions and lobbying. 28 of 90 counties selected. Differential trends in treatment and control groups. Compares those below and above 50 years of age (large age span in both groups) in treatment and control regions. Do not control for changes in labour market conditions (in exit to employment) or policy endogeneity. Not specified what happens to those above age 50 (or below) not fulfilling work requirement. Uncertainty regarding eligibility. Lalive (2007) says potential benefit duration (PBD) changes from 39 to 52 weeks at age 50 in non‐participating regions (control group). Lalive covers same reform/period/country. Lalive shows e.g. policy endogeneity matters.	It is effectively a Regression discontinuity design but they do not consider it to be so. In a footnote (7) they refer to arguments in Micklewright & Nagy (1995) that there is no selectivity due to the reform (no data, just arguments). Consider all confounders (except ethnicity and add more) but do not show imbalances on the restricted sample used for analysis (only for the full sample where there are some important imbalances, see p. 6). The reform also decreased the level of Unemployment insurance benefits. Treated are inflows in January 1993 and control is inflows in December 1992
**Method for identifying relevant confounders described by researchers. Yes/No ‐ if Yes describe the method used.**	Some discussion.	Some (p. 5)
**Relevant confounders described (See relevant sheet and list confounders and note if they were considered, precise, imbalanced or adjusted)**	No imbalances are shown. Education missing. Only initial labour market conditions and age. More is added.	Consider all confounders (except ethnicity and adds more) but do not show imbalances on the restricted sample used for analysis (only for the full sample)
**Method used for controlling for confounding (At design state)**	PBD was extended for workers above age 50 ‐ from 52 to 209 weeks ‐ in specific regions only. Compares those below and above 50 years of age in treatment and control regions. Difference‐in‐difference‐in‐difference.	Legislative changes and individual variation due to labour market history. Use the month before and after reform.
**Method used for controlling for confounding (At analysis stage)**	Proportional hazard rate	Proportional hazard rate with piecewise‐constant baseline hazard.
**Notes**		Can only follow individuals till Unemployment insurance expire

## Footnotes

1 The replacement rate is the ratio of the unemployment benefit to that of previous earnings.

2 For a 40‐year‐old single worker without children and with a 22‐year employment record.

3 The maximum duration was also around six months in the Czech Republic, the Slovak Republic, and the United Kingdom.

4 An alternative could be attaching behavioural conditions in terms of required job search and required acceptance of job offers to benefit receipt.

5 Extensions of benefits are included as well as long as there is a comparison of short versus longer entitlement periods.

6 The number of unfilled jobs expressed as a proportion of the labour force.

7 Extensions of benefits are included as well as long as there is a comparison of short versus longer entitlement periods.

8 Members of the review team at SFI Campbell were: the research assistants Julie Kaas Seerup, Ulrik Højmark Pedersen and Bjørn Christian Viinholt Nielsen.

9 This risk of bias model was introduced by Prof. Reeves at a workshop on risk of bias in non‐randomised studies at SFI Campbell, February 2011. The model is a further development of work carried out in the Cochrane Non‐Randomised Studies Method Group (NRSMG).

10 See next page for an explanation of the terms selection bias and confounding.

11 The reason for this is that unemployment implies a loss of skills or that long periods of unemployment lead to a loss of self‐confidence. This “genuine” duration dependence should not be confused with sorting which is another mechanism.

12 The following description of hazard rates is based on Jenkins (2005) and van den Berg (2001).

13 The following description of estimation is based on Lancaster, 1990.

14 One study did not report hazard ratios but provided results measured as the difference in mean duration

15 One additional study (Winter‐Ember, 1998) analysed unemployed in Austria in the same period but the study was coded with too high risk of bias and was excluded from the data synthesis.

16 Number of vacant jobs per unemployed

17 The study authors use quotations marks.

18 Avoiding overlap of data samples, 15 additional studies could have been used in the data synthesis.

19 Members of the review team at SFI Campbell were: the research assistants Julie Kaas Seerup, Ulrik Højmark Pedersen and Bjørn Christian Viinholt Nielsen.

20 This risk of bias model was introduced by Prof. Reeves at a workshop on risk of bias in non‐randomised studies at SFI Campbell, February 2011. The model is a further development of work carried out in the Cochrane Non‐Randomised Studies Method Group (NRSMG).

21 See user guide for unobservables
